# Developing HaloTag and SNAP‐Tag Chemical Inducers of Dimerization to Probe Receptor Oligomerization and Downstream Signaling

**DOI:** 10.1002/anie.202506830

**Published:** 2025-07-24

**Authors:** Michael Trumpp, Blaise Gatin‐Fraudet, Kjell Bruckmann, Wiktor Burdzinski, Kilian Roßmann, Joshua Levitz, Petra Knaus, Jerome Jatzlau, Johannes Broichhagen

**Affiliations:** ^1^ Institute of Chemistry and Biochemistry – Biochemistry Freie Universität Berlin Berlin Germany; ^2^ International Max Planck Research School for Biology and Computation Berlin Germany; ^3^ Leibniz‐Forschungsinstitut für Molekulare Pharmakologie Berlin Germany; ^4^ Berlin School for Regenerative Therapies Berlin Germany; ^5^ Department of Biochemistry Weill Cornell Medicine New York NY USA

**Keywords:** BMP/TGFβ and TrkB signaling, Chemical inducers of dimerization, FRET sensor, HaloTag, Protein proximity, Receptor oligomerization, SNAP‐Tag

## Abstract

Controlling protein–protein interactions is critical for dissecting signaling pathways, especially those initiated by ligand‐receptor interactions, which alter receptor oligomerization and drive downstream signaling cascades. Traditional methods for driving protein–protein complexes use antibodies that face limitations in terms of stoichiometry, geometric rigidity, and antibody specificity. Chemical inducers of dimerization (CIDs) for fusion proteins such as HaloTag (Halo) and SNAP‐Tags (SNAP) offer precise and covalent control of protein proximities, overcoming limitations of antibody‐dependent methods. In this study, we expand the toolkit of Halo and SNAP CIDs with (1) benzylguanine (BG) and HaloTag ligand (HTL) crosslinkers featuring varying polyethylene glycol linker lengths and update this kit with (2) a FRET‐based dimerizing sensor to induce and verify protein proximity. Here we establish our CIDs on extracellularly Halo‐ and SNAP‐tagged TGFβ, BMP, neurotrophic factor, and metabotropic glutamate receptors, thereby elucidating the signaling potential of ligand‐independent dimerization in a heteromeric fashion.

## Graphical abstract

Controlling protein interactions is key to studying signaling. Antibody methods have limits, but chemical inducers of dimerization (CIDs) using HaloTags and SNAP‐Tags offer precise control. We present CIDs to covalently link heteromers with varied linkers and a FRET‐based sensor. Applied to different tagged cell surface receptors, this allows study of proximity‐induced, ligand‐independent signaling, including an inherent optical readout.

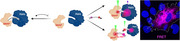

## Introduction

To induce, control, stabilize, and measure protein–protein proximity are pivotal prerequisites to understand and unravel protein–protein interactions and their mechanisms. This is particularly important in signal transduction, where extracellular ligands bind to receptors and can trigger dimerization or oligomerization and, in turn, initiate downstream cascades. In this context, applying methods for inducing and measuring protein–protein proximity is critical for deciphering the cellular effects of receptor quaternary structure changes. Common techniques, such as using antibodies to bring together two proteins of interest (POIs), pulling down potential interaction partners,^[^ ^1^ ^]^ or detecting proximities via patch/FRAP^[^ ^2^ ^]^ or proximity ligation assays^[^ ^3^, ^4^ ^]^ are widely used. However, these approaches are often limited by factors such as undefined stoichiometries, rigid geometrics, and the limited availability of specific antibodies. Chemical inducers of dimerization (CID) are an attractive alternative and were first implemented 30 years ago by Schreiber and Crabtree using synthetic ligands for dimerization of an artificial myristylated receptor.^[^ ^5^ ^]^ Since then, a diverse range of CID systems has been developed, spanning from caged systems enabling spatiotemporal control of dimerization to systems designed for reversible dimerization.^[^ ^6^, ^7^ ^]^ To use CIDs in a broader range of applications and in a general mode that is not limited to only one specific protein pair, they can be combined with the use of self‐labeling tags, for instance hAGT and DHFR,^[^ ^8^ ^]^ SNAP and FKBP,^[^ ^9^ ^]^ the S‐CROSS^[^ ^10^ ^]^ system, and recently, between Halo or SNAP. To date, only a limited number of CIDs have been developed for Halo and SNAP, despite their combined advantages of being highly selective, forming covalent bonds with their cargoes, and offering the fastest kinetics on the market.^[^ ^11^, ^12^ ^]^ To homodimerize SNAP‐Tags, a chemical genetic system consisting of an incorporated BG at the 5′‐hydroxyl group of double‐stranded DNA molecules (DNA‐BG)_2_ (20‐mer) was developed.^[^ ^13^ ^]^ The most notable CIDs, designed to induce dimerization of intracellular proteins fused with Halo and SNAP, are the HaXS molecules,^[^ ^14^ ^]^ later optimized to be photo‐cleavable.^[^ ^15^ ^]^ Recently, a more modular approach was reported, integrating a hetero‐bifunctional cyanine fluorophore with a Halo or SNAP substrate at one side and a functional group for attachment of a molecule of interest to the other, allowing, in principle, the generation of a CID for Halo and SNAP with an integrated cyanine fluorophore.^[^ ^16^ ^]^

To expand those Halo and SNAP CIDs, we developed A) a series of nonfluorescent crosslinkers featuring three distinct polyethylene glycol (PEG) linker lengths, enabling the induction of protein proximities at varying distances; and B) an intramolecular Förster resonance energy transfer (FRET)‐based sensor to confirm induced protein proximity based on a PEG‐coupled Janelia Fluor 549 (JF_549_) donor and 646 (JF_646_) acceptor pair. In the following study, we established and used our dimerizer molecules on Halo‐ and SNAP‐tagged bone morphogenetic protein (BMP) receptors, transforming growth factor β (TGFβ) receptors, neurotrophic factor receptor TrkB and, as a constitutive dimer, the metabotropic glutamate receptor 2 (mGluR2).^[^ ^17^, ^18^, ^19^ ^]^ These receptor classes activate signaling through distinct oligomerization modes. BMP and TGFβ binding to hetero‐tetrameric receptor complexes, consisting of two type I and two type II receptors, induces downstream SMAD phosphorylation.^[^ ^20^ ^]^ Brain‐derived neurotrophic factor (BDNF), the high‐affinity ligand for the TrkB receptor tyrosine kinases, is thought to induce homodimerization to mediate ERK phosphorylation.^[^ ^21^ ^]^ Anecdotally, evolution came up with natural inducers of dimerization long time ago, since BDNF and BMPs/TGFβs are dimers themselves. For a small subset of BMP/TGFβ type I and type II receptor pairs, distinct oligomerization modes have been described, such as (1) BMP‐induced signaling complex (BISC) for BMP2‐ALK3/BMPR2,^[^ ^22^ ^]^ (2) preformed ALK3/BMPR2 heterotetramers, and (3) preformed TGFβR2 or ALK5 homodimers.^[^ ^23^ ^]^ However, the mode of oligomerization remains to be shown for the vast majority of receptor combinations in the absence or presence of ligands. Our developed Halo‐SNAP CIDs will allow to address these questions and, moreover, present broadly applicable tools to induce and investigate protein–protein proximities.

## Results and Discussion

### Inducing Proximity of Halo‐ and SNAP‐Tagged Receptors by Nonfluorescent Dimerizers

**Figure 1:**
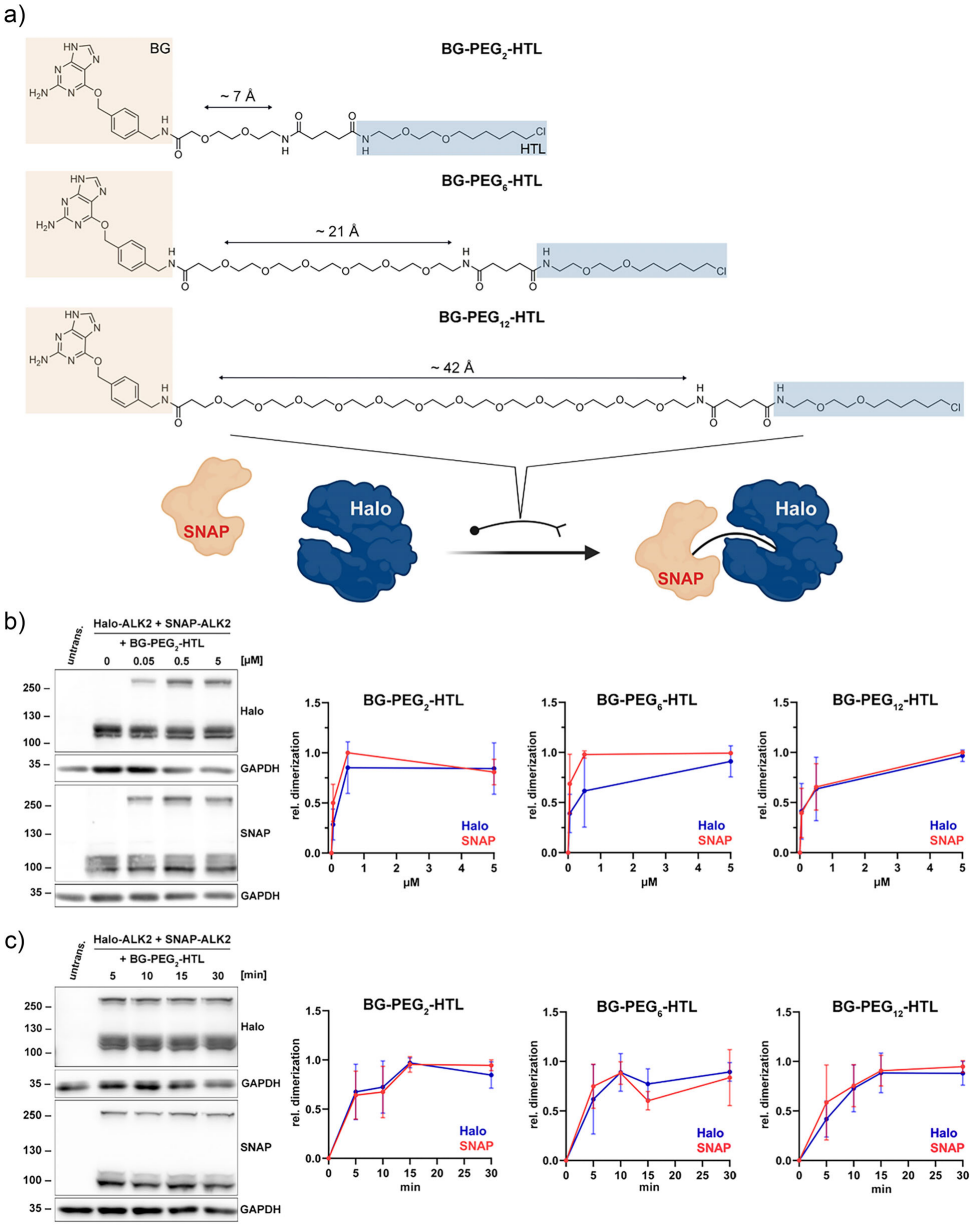
Establishment of BG‐PEG*_n_*‐HTL dimerizer molecule application conditions. a) Structural overview of the Halo‐SNAP dimerizer set (BG‐PEG_2_‐HTL, BG‐PEG_6_‐HTL, and BG‐PEG_12_‐HTL) with their approximate lengths. Benzylguanine (BG) groups are highlighted in beige, Halo‐tag ligand (HTL) in blue. Sketch visualizes the binding of SNAP‐tag to BG group and Halo‐Tag to HTL of the dimerizing molecules, bridging the two enzyme tags covalently. b) and c) (left) Immunoblot of HEK293T cells transiently expressing Halo‐ALK2 and SNAP‐ALK2 stimulated with BG‐PEG_2_‐HTL concentrations from 0.05 to 5 µM for 15 min (b), or 0.5 µM from 5 to 30 min (c). GAPDH serves as loading reference. Heterodimerized receptors exhibit a weight of ∼220 kDa with the corresponding monomeric Halo‐ (120 kDa) or SNAP‐tagged (100 kDa) receptors. (b and c) (right) Densitometric quantification of Halo/SNAP dimer bands relative to GAPDH levels, *n* = 3 independent experiments.

To bring stoichiometrically defined Halo‐ and SNAP‐tagged receptor pairs into proximity and to investigate the influence of receptor vicinity on the induction of downstream signaling, we synthesized HaloTag and SNAP‐Tag dimerizer molecules with linker lengths that vary from ∼7 to ∼42 Å (BG‐PEG_2_‐HTL, BG‐PEG_6_‐HTL, and BG‐PEG_12_‐HTL) (Figure 1a; see Supporting Information). First, we validated the general binding abilities of the various dimerizer molecules by incubating them with recombinantly expressed SNAP‐Halo fusion proteins, followed by mass spectrometry analysis to determine full labeling (Figure S1A,B). Next, immunoblot studies using HEK293T and COS7 cells transiently expressing BMP receptors Halo‐ALK2 and/or SNAP‐ALK2 confirmed the formation of protein dimers only in the presence of both Halo and SNAP, independent of the cell type (Figure S1C,D). The covalent bond formed by the dimerizers between these two fusion proteins is maintained under denaturing conditions, allowing the investigation of heterodimer formation (e.g., Halo‐ALK2/SNAP‐ALK2 ∼250 kDa) using Western blot studies. To define optimal application conditions for the dimerizer variants, HEK293T cells transiently expressing Halo‐ALK2 and SNAP‐ALK2 were stimulated with increasing concentrations (0, 0.05, 0.5, and 5 µM) of each dimerizer. Regardless of the PEG linker length, all dimerizers achieved saturated dimerization at a concentration of 0.5 µM, in line with the previously reported working range for Halo/SNAP CIDs^[^ ^14^ ^]^ (Figures 1b and S2A). Furthermore, we conducted kinetic studies for all dimerizer variants. In these experiments, 0.5 µM of each dimerizer was applied for 5, 10, 15, or 30 min to HEK293T cells transiently expressing Halo‐ALK2 and SNAP‐ALK2. The highest dimerization rates for all dimerizer variants were observed after 10–15 min (Figures 1c and S2B). Next, we investigated the cell permeability properties of the dimerizers. Using the same cell expression conditions, we first depleted the cell surface Halo‐receptor pool using an excess of a cell‐impermeable dye (Alexa 660‐HTL), followed by incubation with the dimerizer variants. Our results demonstrated that the dimerizers can penetrate the cell membrane and facilitate receptor dimer formation exclusively within cells. However, for tagged BMPRs, this occurred at a significantly lower efficiency compared to dimerization of the cell surface pool (Figure S2C).

### Ligand Versus Dimerizer‐Induced Receptor Signaling

**Figure 2:**
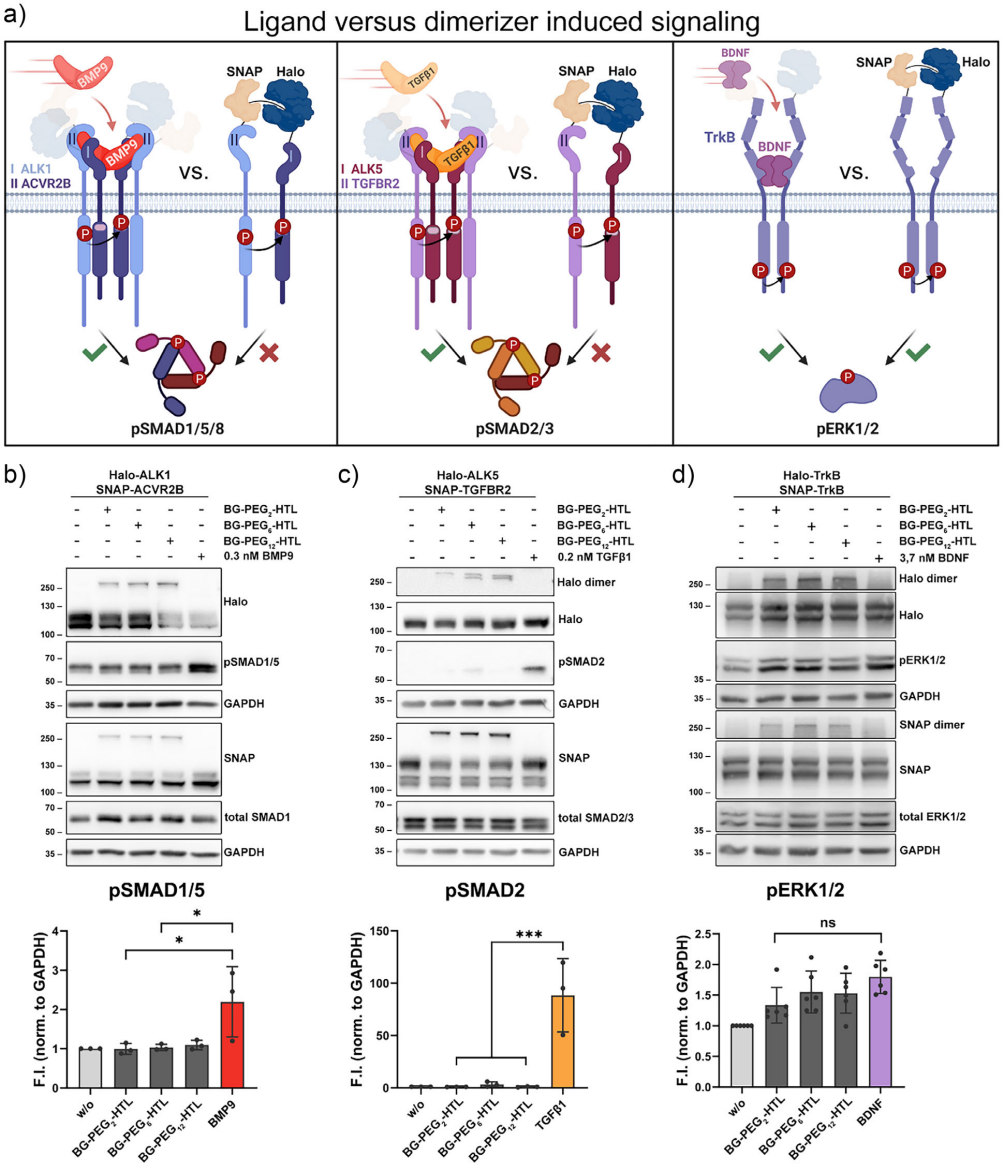
Inducing receptor activation by forced receptor proximity. a) Summary scheme of ligand versus dimerizer‐induced downstream signaling of the BMP, TGFβ, and neurotrophic factor‐binding receptor family classes. Whereas BMP9 and TGFβ1 assemble heterotetrameric receptor complexes, BDNF induces homodimerization of TrkB. The dimerizing molecules are used for either a hetero‐oligomeric receptor complex or a homo‐dimeric receptor complex to study potential impact in downstream signal transmission. b) Immunoblot analysis of HEK293T cells transiently expressing Halo‐ALK1 and SNAP‐ACVR2B stimulated with respective dimerizer or 0.3 nM BMP9 with pSMAD1/5 protein levels as a downstream signaling readout. Total SMAD1 serves as control and GAPDH as loading reference. c) Immunoblot analysis of HEK293T cells transiently expressing Halo‐ALK5 and SNAP‐TGFBR2 stimulated with respective dimerizer or 0.2 nM TGFβ with pSMAD2 protein levels as downstream signaling readout, total SMAD2/3 as control and GAPDH as loading reference. d) Immunoblot analysis of HEK293T cells transiently expressing Halo‐TrkB and SNAP‐TrkB stimulated for 30 min with respective dimerizer or 3.7 nM BDNF with pERK1/2 protein levels as downstream signaling readout, total ERK1/2 as control and GAPDH as loading reference. (b–d) Below, densitometric quantification of pSMAD1/5, pSMAD2, and pERK1/2 levels for unstimulated, stimulated with respective dimerizer or ligand relative to GAPDH are presented as fold induction (F.I.), *n* = 3 independent experiments. Significance was calculated within respective groups using one‐way ANOVA and Tukey's multiple comparisons test, *p < 0.05, ***p < 0.001, ns = not significant.

After optimizing the working conditions for the dimerizing molecules, we investigated whether enforced receptor proximity could trigger downstream signaling responses comparable to those induced by their respective high‐affinity ligands. To test this, we used the dimerizer variants to induce proximity between type I and type II receptors from the BMP (ALK1 and ACVR2B) and TGFβ (ALK5 and TGFBR2) receptor superfamilies, as well as a member of the neurotrophic factor receptor family (TrkB). We then compared the resulting signaling responses to those elicited by their known high‐affinity ligands (Figure 2a–d). We quantified receptor dimerization and assessed phosphorylation levels of BMP, TGFβ, and BDNF‐sensitive SMAD1/5, SMAD2/3, and ERK1/2, respectively (Figure 2b–d). The forced proximity of BMP receptor family members Halo‐ALK1 and SNAP‐ACVR2B showed no pSMAD1/5 induction, while stimulation with their high‐affinity ligand BMP9 led to elevated pSMAD1/5 levels (Figures 2b and S3A). Similarly, no pSMAD2/3 induction by forced dimerization of Halo‐ALK5 and SNAP‐TGFBR2 was achieved, while stimulation with their high‐affinity ligand TGFβ1 led to strongly elevated pSMAD2/3 levels (Figures 2c and S3B). In contrast, forced dimerization of Halo‐TrkB and SNAP‐TrkB resulted in elevated pERK1/2 levels, comparable to stimulation with the high‐affinity ligand BDNF (Figures 2d and S3C). Consequently, we investigated if 15 min predimerization affects the signaling competence of the different ligands. Whereas BDNF and dimerizer stimulation led to increased phosphorylation of ERK1/2, this was not enhanced if TrkB receptors were predimerized before BDNF stimulation. Similarly, no changes in signaling competence were observed for TGFβ1 and BMP9 (Figure S4). Overall, this highlights the usability of the dimerizer to induce receptor proximity and downstream signaling of complexes that signal as dimers (TrkB), while those requiring a hetero‐tetrameric complex of two type I and two type II receptors (BMP and TGFβ receptors) are not inducible by the used dimerizers.

### Fluorescent Dimerizer MT36—A FRET Sensor to Induce and Confirm Halo‐ and SNAP‐Tag Proximity

**Figure 3:**
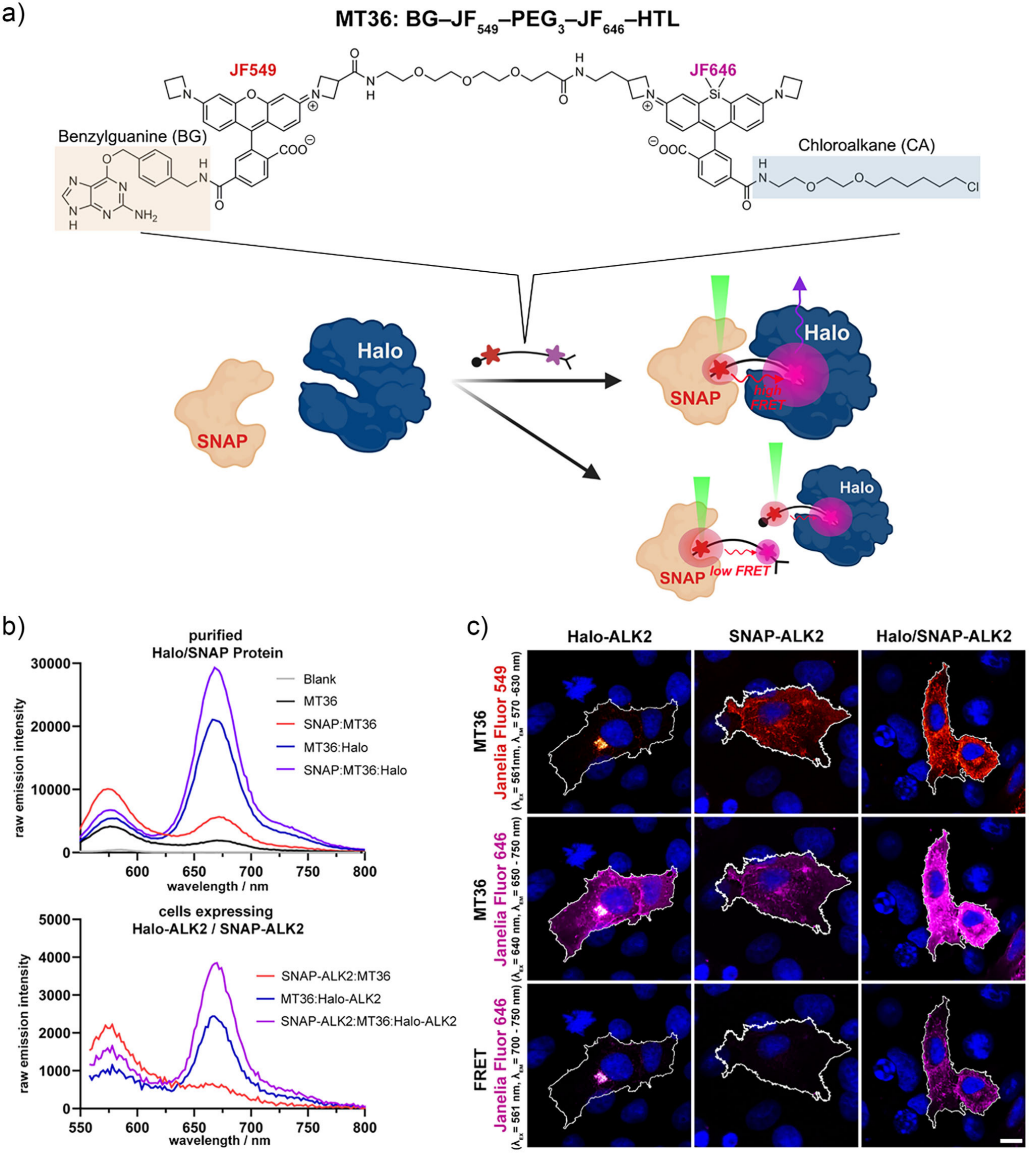
MT36: A fluorescent dimerizer tool for inducing and confirming protein proximity. a) Structure and working principle of fluorescent dimerizer MT36 (HTL–JF_549_–PEG_3_–JF_646_–BG). If Halo and SNAP are both bound, MT36 is in a fully active state. By exciting the JF_549_, energy is transferred via FRET to JF_646_, which in turn emits infrared. This emission is lower if only one site of MT36 is bound to either Halo or SNAP. b) Emission FRET spectra measured in raw emission intensity (*λ*_ex_ = 550 nm, *λ*_em_ = 560–800 nm) of unbound, partially, and fully bound MT36 dimerizer to soluble purified Halo and/or SNAP proteins (up), or cells transiently expressing Halo‐ALK2 and/or SNAP‐ALK2 (below). Raw emission intensity values were measured as triplicates and for cell measurement as quadruplicates. c) Images are acquired in red channel for JF_549_ emission (*λ*_ex_ = 561 nm, *λ*_em_ = 570–630 nm), in infrared channel for JF_646_ emission (*λ*_ex_ = 640 nm, *λ*_em_ = 650–750 nm), or FRET channel (excitation of JF_549_, *λ*_ex_ = 561 nm, *λ*_em_ = 700–750 nm). Scale bar ≙ 10 µm.

In order to resolve with subcellular resolution where two POIs come together, we designed a FRET‐based dimerizer. We synthesized MT36, which is composed of a BG attached to JF_549_ scaffold that is further derivatized to be linked via a PEG_3_ chain to a JF_646_ backbone, ultimately attached to HTL (Figure 3a; see Supporting Information). This cell‐permeable molecule is capable of binding and dimerizing Halo and SNAP in different cellular contexts (Figure S5A–C) and enables visualization of their proximity via FRET. Given the fluorogenicity of both red and far‐red fluorophores, MT36 should achieve maximal fluorescent output only when covalently bound to both SNAP and Halo. Accordingly, once JF_549_ is excited, FRET is supposed to be maximally induced, allowing JF_646_ for far‐red emission. On the flip side, when only one site of MT36 is bound to either the Halo or SNAP, JF_646_ emission should be markedly reduced. We tested and confirmed each of these hypotheses by incubating MT36 with recombinantly expressed and purified Halo and SNAP proteins^[^ ^24^ ^]^ (Figure 3b, upper panel) and, additionally, on HEK293T cells transiently overexpressing Halo‐ALK2 and SNAP‐ALK2 (Figure 3b, lower panel). We then measured the emission profiles of both dyes (*λ*_Ex_ = 500 nm, *λ*_Em_ = 550–800 nm) with a plate reader of unbound, partially bound, and fully bound MT36 dimerizer. The highest emission intensity at 670 nm was observed only if both Halo and SNAP were bound, while single‐bound states showed lower emission intensities (Figure 3b). Whereas 670 nm emission for the single‐bound Halo state was lower, the relative increase to 560 nm reflected a partially activated state enabling FRET (Figure 3b). In contrast, when SNAP alone is bound, its highest emission intensity can be observed at 560 nm (Figure 3b). As anticipated, the overall signal intensity measured in cells incubated with MT36 is lower compared to that of Halo and SNAP incubated with MT36 in vitro. Nevertheless, this demonstrates the capability to assess states of covalently dimerized tags by means of FRET (Figure 3b). To gain a better understanding of this behavior, we determined the Förster radius of JF_549_ and JF_646_ to be *R*_0_ = 5.85 nm (Figure S6A), which places MT36 with a maximal distance of approximately 2 nm (by PEG_3_ spacing) in ideal proximity when dyes are fully zwitterionic. We further tried to estimate FRET efficiency upon labeling and titrated Halo to SNAP:MT36, recording emission spectra (Figure S6B). This allowed us to integrate the area under curve for donor and acceptor, which may serve as a proxy for a degree of dimerization (Figure S6C,D). Fluorescence lifetime microscopy (FLIM) served as a proxy to determine the average efficiency of SNAP:MT36:Halo, which we found to be *E*_FRET_ = 23% (Figure S6C,D, and see Supporting Information for discussion). Importantly, a similar effect could be observed testing MT36 by more sensitive confocal microscopy. To visualize the different fluorescence intensities of the MT36‐bound states, we used COS7 cells transiently expressing Halo‐ALK2, SNAP‐ALK2, or both together and excited and measured the red and far‐red channels JF_549_ and JF_646_ alone, or excited JF_549_ and measured the JF_646_ emission as the FRET channel (*λ*_Ex_ = 549 nm, *λ*_Em_ = 700–750 nm). By this we were able to resemble the trends of beforehand measured FRET emission spectra, using confocal microscopy (Figures 3c and S5D). To investigate the influence of the Halo and SNAP on the FRET sensor functionality, we switched the substrate for each tag (HTL and BG) to the opposite side of the molecule by means of organic synthesis, generating MT37, which is composed of an HTL attached to JF_549_ coupled via PEG_3_ to JF_646_ attached to BG, which, however, performed more poorly compared to MT36 (Figures S7A and SI). In particular, it eliminates the ability to distinguish between the state where only Halo is bound and the state where both the Halo and SNAP are bound. As a result, it makes this configuration unsuitable as a sensor for confirming the proximity of Halo and SNAP (Figure S7B,C).

### MT36 Confirms Constitutive Dimers of mGluR2 with Higher FRET Intensities

Next, we tested how the MT36 FRET sensor performs with mGluR2, a metabotropic glutamate receptor (mGluR), which forms constitutive dimers, independent of the presence of ligand.^[^ ^25^, ^26^ ^]^ As described above, COS7 cells transiently expressing Halo‐mGluR2 and/or SNAP‐mGluR2 were incubated with MT36, or JF_549_‐BG and JF_646_‐HTL as controls and analyzed via confocal microscopy (Figure S8A–C). As expected, the highest FRET intensities were measured upon double‐expression of Halo/SNAP mGluR2. Further, the expression of only Halo‐mGluR2 exhibited a lower FRET emission, recapitulating the above‐described difference between single and double‐bound states (Figure 3b). Similarly, FRET signals were detected when Halo/SNAP mGluR2 double‐expressing cells were incubated with single dyes, confirming the capability of mGluR2 to form constitutive dimers (Figure S8B). However, the FRET intensity of single dyes is weaker compared to double‐bound MT36 (Figure S8B).

### Inducing and Verifying TrkB Proximity with MT36 Leads to Activation of Downstream Signal Transduction

**Figure 4:**
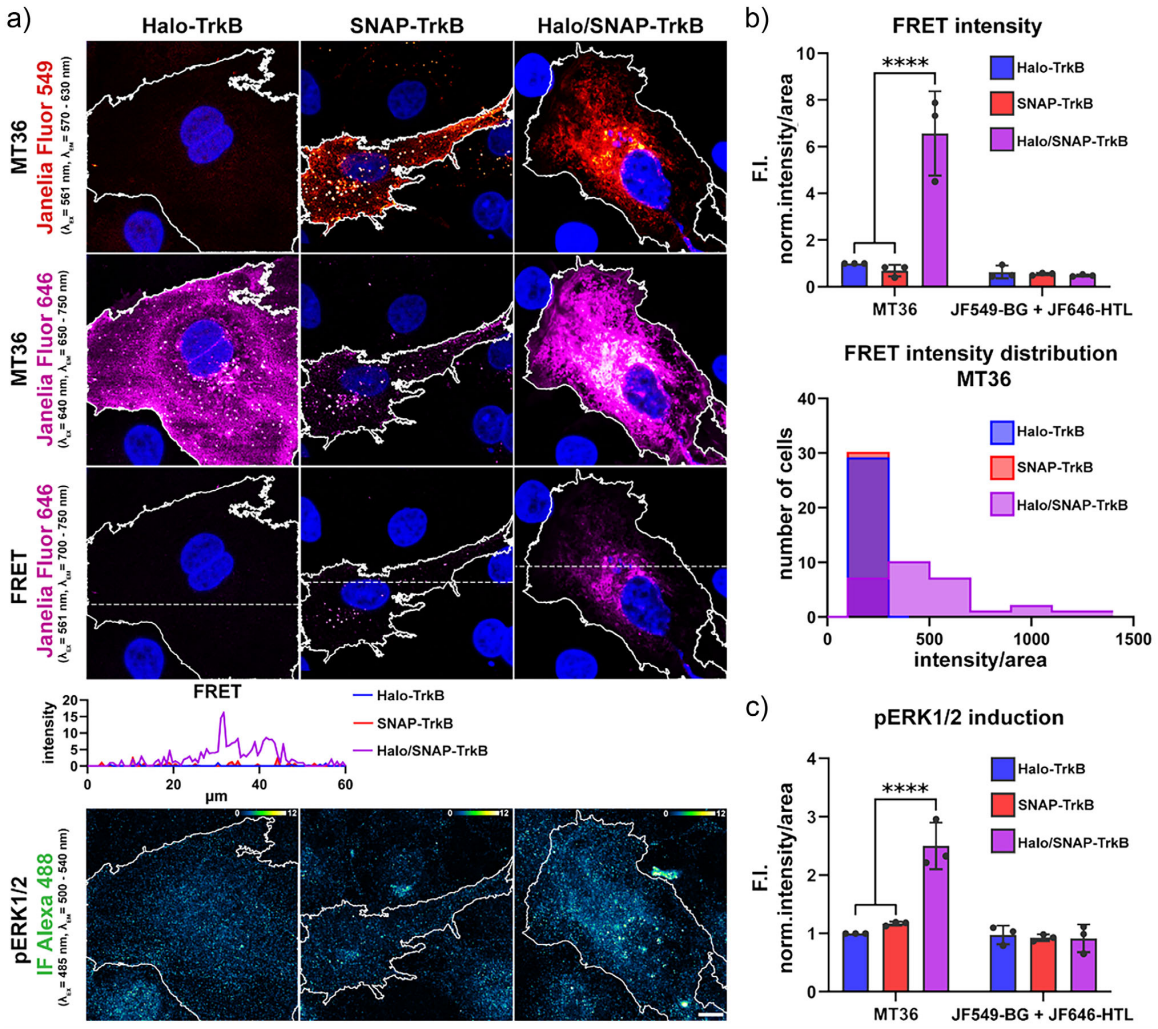
FRET‐based validation of TrkB dimerization and pERK1/2 induction. a) Representative confocal microscopy images of COS7 cells transiently expressing Halo‐TrkB and/or SNAP‐TrkB incubated with MT36 and DAPI. Images are acquired in red channel for JF_549_ emission (*λ*_ex_ = 561 nm, *λ*_em_ = 570–630 nm), in infrared channel for JF_646_ emission (*λ*_ex_ = 640 nm, *λ*_em_ = 650–750 nm) or FRET channel (excitation of JF_549_, *λ*_ex_ = 561 nm, *λ*_em_ = 700–750 nm). A line scan was performed to assess fluorescence intensity within the FRET channel, and its path is highlighted as dashed lines. Scale bar ≙ 10 µm. b upper and c) Mean fluorescence FRET and pERK1/2 intensities per cell area were normalized to Halo‐TrkB. Data shown as fold induction (F.I.). b lower) Grouped area normalized mean FRET intensity values of each cell (*n* = 3 with 10 cells each). Significance was calculated within respective groups using two‐way ANOVA and Šídák's multiple comparisons test, ****p < 0.0001. *n* = 3 independent experiments with 10 cells per condition.

Having confirmed the ability of MT36 to induce proximity, we revisited the activation of TrkB using confocal microscopy to visualize receptor activation. Here, COS7 cells transiently expressing Halo‐TrkB, SNAP‐TrkB, or both together were incubated with MT36, or as control, with JF_549_‐BG, JF_646_‐HTL (Figures 4 and S9). To confirm the induced receptor proximity, we excited and imaged the single channels for JF_549_ and JF_646_ or excited JF_549_ and imaged JF_646_ as the FRET channel. Additionally, to assess the resulting receptor activation, we performed immunofluorescence (IF) staining of pERK1/2 (Figures 4a and S9A). Using this approach, we can clearly differentiate between the single and double bound states of the dimerizer, with the brightest signal observed in the FRET channels of cells expressing both Halo‐TrkB and SNAP‐TrkB incubated with MT36 (Figure 4a). Further, the single dye conditions show no specific FRET signal, which is in line with TrkB not forming constitutive receptor dimers (Figure S9B). In addition, we quantified the mean fluorescence intensities per cell of all channels by assessing the raw integrated density of each cell followed by cell area normalization. To compare the dimerization‐dependent effect on FRET emission and pERK1/2 intensity, averaged intensity/cell values were normalized to the Halo‐only receptor condition (Figure 4b,c). The quantification confirms the highest FRET intensities for MT36‐treated cells expressing both Halo‐TrkB and SNAP‐TrkB. Interestingly, a low FRET signal is observed for Halo‐TrkB or SNAP‐TrkB only expressing cells, suggesting that FRET signals are only efficiently emitted in the MT36 double‐bound state, thereby confirming the induced proximity of the TrkB by MT36 (Figure 4b), recapitulating the results obtained for ALK2 and mGluR2 (Figures 3c and S8). Further, by grouping the area‐normalized mean fluorescence intensity values of each cell, we could display the distribution of single cell FRET emission values of the different bound states (Halo, SNAP, or double bound) underlining the elevated FRET intensities upon double binding by both Halo and SNAP (Figure 4b). In concert with this, we quantified the pERK1/2 fluorescence signal, which was significantly higher in the MT36 double‐bound condition (Figure 4c). This highlights that MT36 can dimerize Halo‐ and SNAP‐tagged TrkB receptors, thereby inducing downstream ERK1/2 phosphorylation, while its FRET abilities allow us to measure and confirm the induced proximity.

### A Dual in‐One Toolkit to Induce Signal Transduction and FRET Assaying

In recent years, Halo and SNAP emerged as popular fusion proteins, being implemented as state‐of‐the‐art tools in a plethora of microscopy studies. The covalent labeling in a 1:1 ratio to their respective substrate enabled us previously to specifically label two different BMP receptor species with organic dyes to investigate receptor complexes using, e.g., STED super‐resolution microscopy.^[^ ^19^ ^]^ Besides labeling receptor populations, we wanted to go a step further and bring pairs of respectively labeled receptors in proximity to study oligomerization modes and resulting downstream signaling. For this, we synthesized three small molecules composed of Halo and SNAP substrates, linked by varying numbers of PEG molecules, resulting in differing dimerizer lengths.

The tools available to bring receptors into close proximity are diverse; however, their application is often constrained to a single context due to their reliance on highly specific protein modifications or the use of targeted antibodies.^[^ ^5^, ^23^, ^27^, ^28^ ^]^ In contrast, using CIDs for Halo‐ and SNAP‐tagged POIs allows for a much broader application range. Here, we applied our Halo‐SNAP CIDs to different receptor classes, such as TrkB receptors and type I/II receptors from the TGFβ and BMP receptor families. Whereas the chemical dimerization of TrkB recapitulates BDNF‐induced phosphorylation of ERK1/2, heterodimerization of one type I and one type II TGFβ/BMP receptor is not sufficient to mimic ligand‐induced activation of SMADs. This is in line with a series of reports showing that ligand‐independent forced proximity of receptor pairs was not sufficient for downstream pathway activation.^[^ ^13^, ^29^, ^30^ ^]^ This highlights that chemical‐induced proximity is sufficient to allow TrkB trans‐activation and downstream pathway activation. In contrast, activation of the BMP/TGFβ signaling cascade requires a ligand to bind two type I and two type II receptors, assembling them into a tetrameric receptor complex. ^[^ ^31^ ^]^ Within the tetramer, the type II receptors activate the type I receptor kinase by transphosphorylation of the GS‐rich motif, allowing phosphorylation of downstream mediators.^[^ ^32^ ^]^ Within this complex, the type I and type II receptors are facing each other in adjacent positions, while the two type I or two type II receptors are in opposite positions.^[^ ^31^ ^]^ Dimerization of constitutively active type I BMP/TGFβ receptors via optogenetic domains or homodimeric dimerization via antibodies was shown to be sufficient to foster downstream signaling.^[^ ^28^, ^33^ ^]^ Here, we show that by bringing single type I and type II receptors artificially in proximity no signal transduction occurs, highlighting additional requirements for BMP/TGFβ signaling. This is in concert with a previous study that illustrates the intricate placement of ALK2 (type I) and BMPR2 (type II) receptor kinases needed for receptor activation.^[^ ^34^ ^]^ Further, it was shown that the relative receptor positioning and spacing within a tetrameric complex differ in dependence on the BMP or TGFβ suggesting the potential for ligand‐specific downstream signaling.^[^ ^35^, ^36^, ^37^ ^]^ In addition, it could be speculated that the ligand induces a structural rearrangement of the receptor complexes allowing for type I receptor activation.

Apart from above‐mentioned HaXS molecules, dimerization has also been achieved genetically by using splitFAST systems^[^ ^38^ ^]^ and green light‐induced dimerization of bacterial vitamin B12 binding domain,^[^ ^39^ ^]^ as well as using light‐oxygen‐voltage (LOV)‐sensing domains for dimerization and activation of fibroblast growth factor receptor 1 (FGFR1), epidermal growth factor receptor (EGFR) and rearranged during transfection (RET).^[^ ^40^ ^]^ The toolbox of published FRET sensors includes a wide range of different working principles based on, e.g., distance change (cleavage‐based, conformational change‐based, or mechanical force‐based), fluorescence property change (pH change‐ or oxidation‐based), or even multicolor FRET pairs.^[^ ^41^ ^]^ However, many of them were developed for one specific protein pair limiting them to a single application or involving various cloning efforts to allow their application in other projects.^[^ ^42^ ^]^ By making use of the Halo and SNAP‐tag system, our developed dimerizing FRET sensor is not limited to a single application but can be used for all Halo and SNAP‐tagged protein combinations. The compound MT36 (BG–JF_549_–PEG_3_–JF_646_–HTL) is combining the advantage of the covalent binding and high target specificity of the Halo and SNAP‐tag,^[^ ^43^ ^]^ with the high photostability and brightness of the rhodamine‐based Janelia Fluor (JF) dyes.^[^ ^44^ ^]^ With no significant difference of the nonfluorescent dimerizer for TrkB activation, and no structural available data for TrkB by, for instance, cryoEM, we chose a PEG_3_ linker for the main reason that its maximal theoretical distance between the covalently attached carbons to the protein labeling site is about the same as for BG–PEG_6_–HTL. Further shortening of the distance might be caused by the dye–protein interactions, restraining degrees of freedom; however, it is still longer than the successfully used BG–PEG_2_–HTL molecule. Apart from the linkerology, the JF_549_–BG and JF_646_–HTL dye pair used in our dimerizer show their emission maximum only when both corresponding substrates are covalently bound to their respective tags. This phenomenon is observable due to their fluorogenicity, as the dyes exist in equilibrium between an open (fluorescent), zwitterionic, and closed lactone form (nonfluorescent).^[^ ^45^, ^46^ ^]^ This mechanism is not yet fully understood, but it suffices to say that once conjugated to Halo or SNAP, the protein surface interaction with the fluorophore favors the open fluorescent conformation.^[^ ^45^, ^47^ ^]^ This is supported by our observation of higher emission values when Halo‐tag is bound directly to either JF_549_ or JF_646_ compared to the SNAP‐tag bound state, backed up by other reports that describe fluorescent signals significantly higher for Halo.^[^ ^24^, ^43^ ^]^ Cohesively, for rhodamines it is proposed that the planar zwitterionic form interacts energetically more favorably with the Halo surface, featuring the fluorescent state of the fluorophore more beneficial compared to SNAP.^[^ ^48^ ^]^ Switching Halo and SNAP substrates of the MT36 to HTL–JF_549_–PEG_3_–JF_646_–BG (MT37) results in an overall lower emission intensity as well as FRET sensitivity and bound state selectivity. These results indicate that Halo should be selected for the acceptor fluorophore in a FRET pair of rhodamine dyes with SNAP. Whereas the MT36 was cell‐permeable, sulfonation of red and far‐red rhodamines is already commonly used to prevent intracellular staining.^[^ ^49^ ^]^ This could be a potential direction to further refine our MT36 dimerizing FRET sensor, adapting it specifically for extracellular dimerization detection and targeted staining.

## Conclusion

In summary, we presented in this study a set of three dimerizer molecules (BG–PEG_2_–HTL, BG–PEG_6_–HTL, and BG–PEG_12_–HTL) to stoichiometrically bring into proximity and covalently link defined Halo‐ and SNAP‐tagged receptor pairs with free‐to‐choose varying linker lengths. We established and tested these on BMP and TGFβ receptors as well as on the neurotrophic factor receptor TrkB. While the BMP or TGFβ pathway could not be activated by simply forcing single type I and type II receptors into a heterodimer, two TrkB receptors were activated by chemically inducing proximity independent of linker length. Further, we developed the dimerizing FRET sensor MT36, which senses via FRET readout the different bound states and forced proximity of Halo‐ and SNAP‐tagged serine/threonine proteases and G protein‐coupled receptors. Collectively these tools allow us to investigate ligand‐independent receptor proximity‐induced signal transduction down to the single‐cell level.

## Materials and Methods

All methods, including synthesis and characterization, in vitro, and *in cellulo* experiments, can be found in the Supporting Information.

## Conflict of Interests

The authors declare no conflict of interest.

## Supporting information



Supporting Information

## Data Availability

The data that support the findings of this study are available in the Supporting Information of this article.

## References

[1] I. M. Evans , K. Paliashvili , Methods Mol. Biol. 2022, 2475, 125–132. 35451753 10.1007/978-1-0716-2217-9_8

[2] S. S. Szilágyi , O. Gutman , Y. I. Henis , Methods Mol. Biol. 2022, 2488, 23–34. 35347680 10.1007/978-1-0716-2277-3_3

[3] M. S. Alam , Curr. Protoc. Immunol. 2018, 123, e58. 30238640 10.1002/cpim.58 PMC6205916

[4] P. L. Mendez , L. Obendorf , P. Knaus , J. Vis. Exp. 2021, 10.3791/62608 34605801

[5] D. M. Spencer , T. J. Wandless , S. L. Schreiber , G. R. Crabtree , Science 1993, 262, 1019–1024. 7694365 10.1126/science.7694365

[6] S. Voß , L. Klewer , Y.‐W. Wu , Curr. Opin. Chem. Biol. 2015, 28, 194–201. 26431673 10.1016/j.cbpa.2015.09.003

[7] C. Aonbangkhen , H. Zhang , D. Z. Wu , M. A. Lampson , D. M. Chenoweth , J. Am. Chem. Soc. 2018, 140, 11926–11930. 30196699 10.1021/jacs.8b07753 PMC6499933

[8] S. Gendreizig , M. Kindermann , K. Johnsson , J. Am. Chem. Soc. 2003, 125, 14970–14971. 14653715 10.1021/ja037883p

[9] S. Feng , V. Laketa , F. Stein , A. Rutkowska , A. MacNamara , S. Depner , U. Klingmüller , J. Saez‐Rodriguez , C. Schultz , Angew. Chem. Int. Ed. 2014, 53, 6720–6723. 10.1002/anie.201402294 24841150

[10] A. Gautier , E. Nakata , G. Lukinavicius , K.‐T. Tan , K. Johnsson , J. Am. Chem. Soc. 2009, 131, 17954–17962. 19916541 10.1021/ja907818q

[11] G. V. Los , L. P. Encell , M. G. McDougall , D. D. Hartzell , N. Karassina , C. Zimprich , M. G. Wood , R. Learish , R. F. Ohana , M. Urh , D. Simpson , J. Mendez , K. Zimmerman , P. Otto , G. Vidugiris , J. Zhu , A. Darzins , D. H. Klaubert , R. F. Bulleit , K. V. Wood , ACS Chem. Biol. 2008, 3, 373–382. 18533659 10.1021/cb800025k

[12] A. Keppler , S. Gendreizig , T. Gronemeyer , H. Pick , H. Vogel , K. Johnsson , Nat. Biotechnol. 2003, 21, 86–89. 12469133 10.1038/nbt765

[13] S. I. Liang , B. van Lengerich , K. Eichel , M. Cha , D. M. Patterson , T.‐Y. Yoon , M. von Zastrow , N. Jura , Z. J. Gartner , Cell Rep. 2018, 22, 2593–2600. 29514089 10.1016/j.celrep.2018.02.031 PMC5916813

[14] D. Erhart , M. Zimmermann , O. Jacques , M. B. Wittwer , B. Ernst , E. Constable , M. Zvelebil , F. Beaufils , M. P. Wymann , Chem. Biol. 2013, 20, 549–557. 23601644 10.1016/j.chembiol.2013.03.010

[15] M. Zimmermann , R. Cal , E. Janett , V. Hoffmann , C. G. Bochet , E. Constable , F. Beaufils , M. P. Wymann , Angew. Chem. Int. Ed. 2014, 53, 4717–4720. 10.1002/anie.201310969 PMC4499241 24677313

[16] C. Maller , F. Schedel , M. Köhn , J. Org. Chem. 2024, 89, 3844–3856. 38413005 10.1021/acs.joc.3c02673 PMC10949230

[17] S. D. Skaper , Methods Mol. Biol. 2018, 1727, 1–17. 29222769 10.1007/978-1-4939-7571-6_1

[18] A. Ellaithy , J. Gonzalez‐Maeso , D. A. Logothetis , J. Levitz , Trends Biochem. Sci. 2020, 45, 1049–1064. 32861513 10.1016/j.tibs.2020.07.008 PMC7642020

[19] J. Jatzlau , W. Burdzinski , M. Trumpp , L. Obendorf , K. Roßmann , K. Ravn , M. Hyvönen , F. Bottanelli , J. Broichhagen , P. Knaus , Commun. Biol. 2023, 6, 34. 36635368 10.1038/s42003-022-04388-4 PMC9837045

[20] D. Yadin , P. Knaus , T. D. Mueller , Cytokine Growth Factor Rev. 2016, 27, 13–34. 26690041 10.1016/j.cytogfr.2015.11.005

[21] M. A. Lemmon , J. Schlessinger , Cell 2010, 141, 1117–1134. 20602996 10.1016/j.cell.2010.06.011 PMC2914105

[22] L. Gilboa , A. Nohe , T. Geissendörfer , W. Sebald , Y. I. Henis , P. Knaus , Mol. Biol. Cell 2000, 11, 1023–1035. 10712517 10.1091/mbc.11.3.1023 PMC14828

[23] A. Guzman , M. Zelman‐ Femiak , J. H. Boergermann , S. Paschkowsky , P. A. Kreuzaler , P. Fratzl , G. S. Harms , P. Knaus , J. Biol. Chem. 2012, 287, 39492–39504. 22961979 10.1074/jbc.M112.387639 PMC3501045

[24] M. Trumpp , A. Oliveras , H. Gonschior , J. Ast , D. J. Hodson , P. Knaus , M. Lehmann , M. Birol , J. Broichhagen , Chem. Commun. (Camb.) 2022, 58, 13724–13727. 36427021 10.1039/d2cc04823j PMC9745879

[25] J. Levitz , C. Habrian , S. Bharill , Z. Fu , R. Vafabakhsh , E. Y. Isacoff , Neuron 2016, 92, 143–159. 27641494 10.1016/j.neuron.2016.08.036 PMC5053906

[26] J. Lee , H. Munguba , V. A. Gutzeit , D. R. Singh , M. Kristt , J. S. Dittman , J. Levitz , Cell Rep. 2020, 31, 107605. 32375054 10.1016/j.celrep.2020.107605 PMC7271767

[27] K. Luo , H. F. Lodish , EMBO J. 1997, 16, 1970–1981. 9155023 10.1093/emboj/16.8.1970 PMC1169800

[28] A. Ramachandran , P. Vizán , D. Das , P. Chakravarty , J. Vogt , K. W. Rogers , P. Müller , A. P. Hinck , G. P. Sapkota , C. S. Hill , eLife 2018, 7, e31756. 29376829 10.7554/eLife.31756 PMC5832415

[29] L. Opalinski , A. Sokolowska‐Wedzina , M. Szczepara , M. Zakrzewska , J. Otlewski , Sci. Rep. 2017, 7, 7121. 28769084 10.1038/s41598-017-07479-z PMC5540934

[30] G. Jiang , T. Hunter , Curr. Biol. 1999, 9, R568–R571. 10469554 10.1016/s0960-9822(99)80357-1

[31] T. D. Mueller , J. Nickel , FEBS Lett. 2012, 586, 1846–1859. 22710174 10.1016/j.febslet.2012.02.043

[32] B. Bragdon , O. Moseychuk , S. Saldanha , D. King , J. Julian , A. Nohe , Cell. Signal. 2011, 23, 609–620. 20959140 10.1016/j.cellsig.2010.10.003

[33] S. Aykul , L. Huang , L. Wang , N. M. Das , S. Reisman , Y. Ray , Q. Zhang , N. Rothman , K. C. Nannuru , V. Kamat , S. Brydges , L. Troncone , L. Johnsen , P. B. Yu , S. Fazio , J. Lees‐Shepard , K. Schutz , A. J. Murphy , A. N. Economides , V. Idone , S. J. Hatsell , J. Clin. Invest. 2022, 132, e153792. 35511419 10.1172/JCI153792 PMC9197526

[34] C. Agnew , P. Ayaz , R. Kashima , H. S. Loving , P. Ghatpande , J. E. Kung , E. S. Underbakke , Y. Shan , D. E. Shaw , A. Hata , N. Jura , Nat. Commun. 2021, 12, 4950. 34400635 10.1038/s41467-021-25248-5 PMC8368100

[35] E. J. Goebel , K. N. Hart , J. C. McCoy , T. B. Thompson , Exp. Biol. Med. (Maywood) 2019, 244, 1530–1546. 31594405 10.1177/1535370219880894 PMC6920667

[36] S. A. Townson , E. Martinez‐Hackert , C. Greppi , P. Lowden , D. Sako , J. Liu , J. A. Ucran , K. Liharska , K. W. Underwood , J. Seehra , R. Kumar , A. V. Grinberg , J. Biol. Chem. 2012, 287, 27313–27325. 22718755 10.1074/jbc.M112.377960 PMC3431715

[37] S. Radaev , Z. Zou , T. Huang , E. M. Lafer , A. P. Hinck , P. D. Sun , J. Biol. Chem. 2010, 285, 14806–14814. 20207738 10.1074/jbc.M109.079921 PMC2863181

[38] S. Bottone , O. Joliot , Z. V. Cakil , L. El Hajji , L.‐M. Rakotoarison , G. Boncompain , F. Perez , A. Gautier , Nat. Methods 2023, 20, 1553–1562. 37640938 10.1038/s41592-023-01988-8

[39] S. Kainrath , M. Stadler , E. Reichhart , M. Distel , H. Janovjak , Angew. Chem. Int. Ed. 2017, 56, 4608–4611. 10.1002/anie.201611998 PMC5396336 28319307

[40] M. Grusch , K. Schelch , R. Riedler , E. Reichhart , C. Differ , W. Berger , Á.l. Inglés‐Prieto , H. Janovjak , EMBO J. 2014, 33, 1713–1726. 24986882 10.15252/embj.201387695 PMC4194103

[41] L. Liu , F. He , Y. Yu , Y. Wang , Front. Bioeng. Biotechnol. 2020, 8, 595497. 33240867 10.3389/fbioe.2020.595497 PMC7680962

[42] N. Soleja , M. Mohsin , Biotechnol. Adv. 2024, 77, 108466. 39419421 10.1016/j.biotechadv.2024.108466

[43] R. S. Erdmann , S. W. Baguley , J. H. Richens , R. F. Wissner , Z. Xi , E. S. Allgeyer , S. Zhong , A. D. Thompson , N. Lowe , R. Butler , J. Bewersdorf , J. E. Rothman , D. St Johnston , A. Schepartz , D. Toomre , Cell Chem. Biol. 2019, 26, 584–592. 30745239 10.1016/j.chembiol.2019.01.003 PMC6474801

[44] J. B. Grimm , B. P. English , J. Chen , J. P. Slaughter , Z. Zhang , A. Revyakin , R. Patel , J. J. Macklin , D. Normanno , R. H. Singer , T. Lionnet , L. D. Lavis , Nat. Methods 2015, 12, 244–250, 3 p following 244–250. 25599551 10.1038/nmeth.3256 PMC4344395

[45] C. A. Hoelzel , X. Zhang , Chembiochem 2020, 21, 935–1946. 10.1002/cbic.202000037 PMC7367766 32180315

[46] L. D. Lavis , Annu. Rev. Biochem. 2017, 86, 825–843. 28399656 10.1146/annurev-biochem-061516-044839

[47] M. S. Frei , M. Tarnawski , M. J. Roberti , B. Koch , J. Hiblot , K. Johnsson , Nat. Methods 2022, 19, 65–70. 34916672 10.1038/s41592-021-01341-x PMC8748199

[48] J. Wilhelm , S. Kühn , M. Tarnawski , G. Gotthard , J. Tünnermann , T. Tänzer , J. Karpenko , N. Mertes , L. Xue , U. Uhrig , J. Reinstein , J. Hiblot , K. Johnsson , Biochemistry 2021, 60, 2560–2575. 34339177 10.1021/acs.biochem.1c00258 PMC8388125

[49] R. Birke , J. Ast , D. A. Roosen , J. Lee , K. Roßmann , C. Huhn , B. Mathes , M. Lisurek , D. Bushiri , H. Sun , B. Jones , M. Lehmann , J. Levitz , V. Haucke , D. J. Hodson , J. Broichhagen , Org. Biomol. Chem. 2022, 20, 5967–5980. 35188523 10.1039/d1ob02216d PMC9346974

