# Supplementary material for: Developing HaloTag and SNAP‐Tag Chemical Inducers of Dimerization to Probe Receptor Oligomerization and Downstream Signaling
Source: Angew Chem Int Ed Engl. 2025 Jul 24;64(35):e202506830. doi: 10.1002/anie.202506830 (PMC12377427; doi:10.1002/anie.202506830)
Supplement: Supplementary file 1 — Supporting Information [file ANIE-64-e202506830-s001.pdf]

Developing HaloTag and SNAP-Tag Chemical Inducers of Dimerization to Probe Receptor Oligomerization and Downstream Signaling

Michael Trumpp[1,2], Blaise Gatin-Fraudet[3], Kjell Bruckmann[1], Wiktor Burdzinski[1,5], Kilian Roßmann[3], Joshua Levitz[5], Petra Knaus[1], Jerome Jatzlau[1,*] & Johannes Broichhagen[3,*]

Supplementary Figures

Figure S1: Functional testing of binding and dimerization capabilities of BG-PEG$_n$-HTL

Figure S2: Investigation of BG-PEG$_n$-HTL application and cell permeability

Figure S3: Full Immunoblots for Figure 2 B – D

Figure S4: Pre-dimerization of BMP/TGFβ and TrkB receptors does not enhance ligand-induced signaling.

Figure S5: Investigation of MT36 FRET efficiency

Figure S6: Investigation of MT36 application and cell permeability

Figure S7: MT37 – a fluorescent dimerizer tool inducing but not confirming protein proximity

Figure S8: FRET based validation of mGluR2 dimerization

Figure S9: Complementary control condition for FRET based validation of TrkB dimerization and pERK1/2 induction.

Supplementary Table

Table S1: Overexpression DNA constructs

Synthesis

Scheme S1 Synthesis pathway of BG-PEG2-HTL, BG-PEG6-HTL, BG-PEG12-HTL

Scheme S2 Synthesis pathway of compounds 3 to 8

Scheme S3 Synthesis pathway of MT36 & MT37

NMR and mass spectrometry

Methods

LCMS and HPLC

Mass spectrometry

Cell culture

Western Blotting and SDS-PAGE

Emission and FRET profiles in vitro and in cellulo

Confocal and lifetime microscopy

Statistical analysis

Supplementary Figures

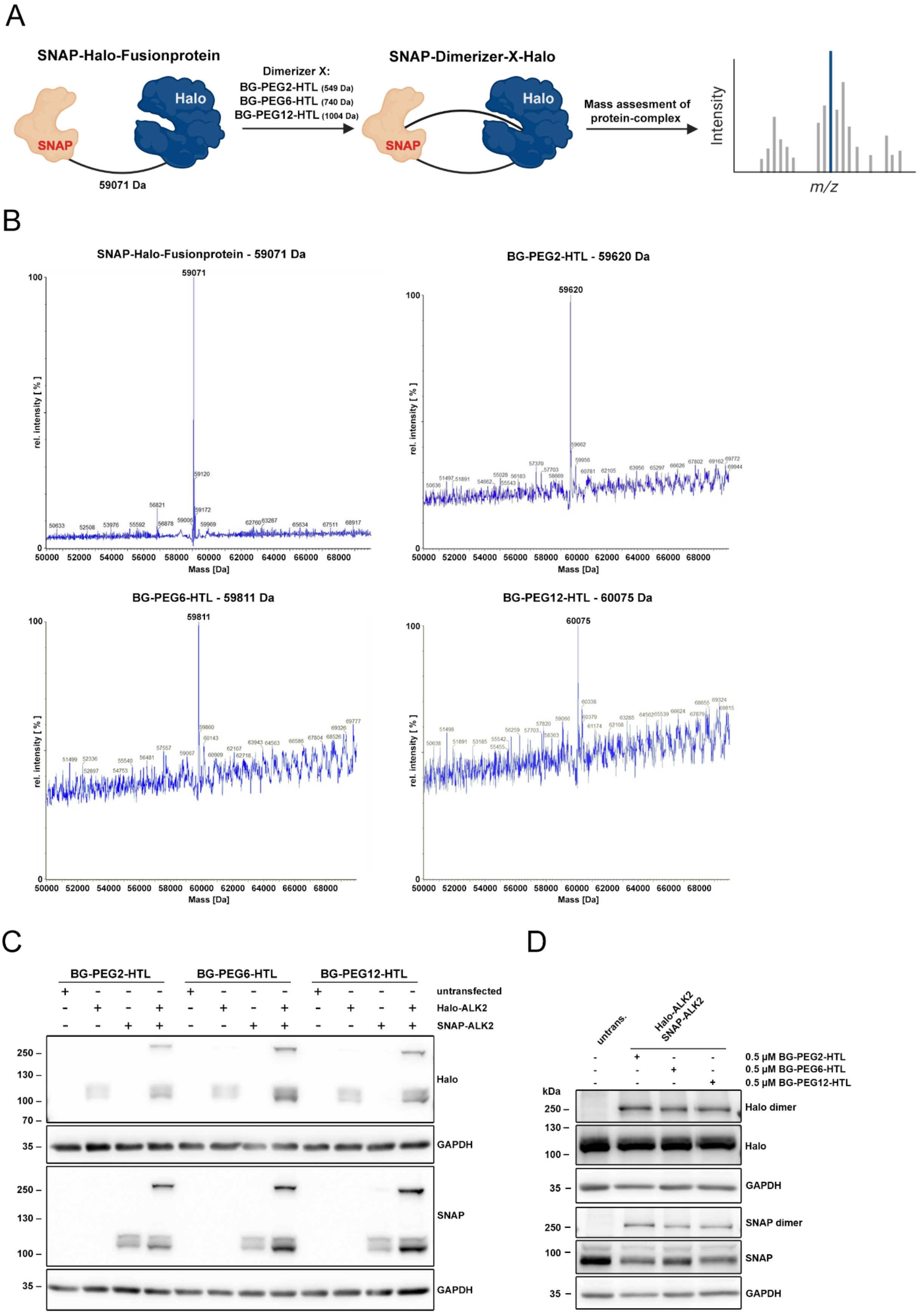

Figure S1: Functional testing of binding and dimerization capabilities of BG-PEG$_n$-HTL. **(A)** Scheme illustrating the method of SNAP-Halo-fusion protein (59071 Da) binding to_BG-PEG2-HTL (549 Da), BG-PEG6-HTL (740 Da), BG-PEG12-HTL (1004 Da). Dimerizer molecules were incubated with a SNAP-Halo-fusion protein followed by mass assessment of the SNAP-Halo-dimerizer complex using mass spectrometry. (**B**) Measured masses of SNAP-Halo Fusion Protein incubated with or without BG-PEG$_n$-HTL. (**C**) Immunoblot analysis of HEK293T cells transiently expressing Halo-ALK2, SNAP-ALK2 or both simultaneously stimulated with BG-PEG$_n$-HTL. GAPDH serves as loading reference. (**D**) Immunoblot analysis of COS7cells transiently expressing Halo-ALK2 and/or SNAP-ALK2 simultaneously stimulated with BG-PEGn-HTL. GAPDH serves as loading reference.

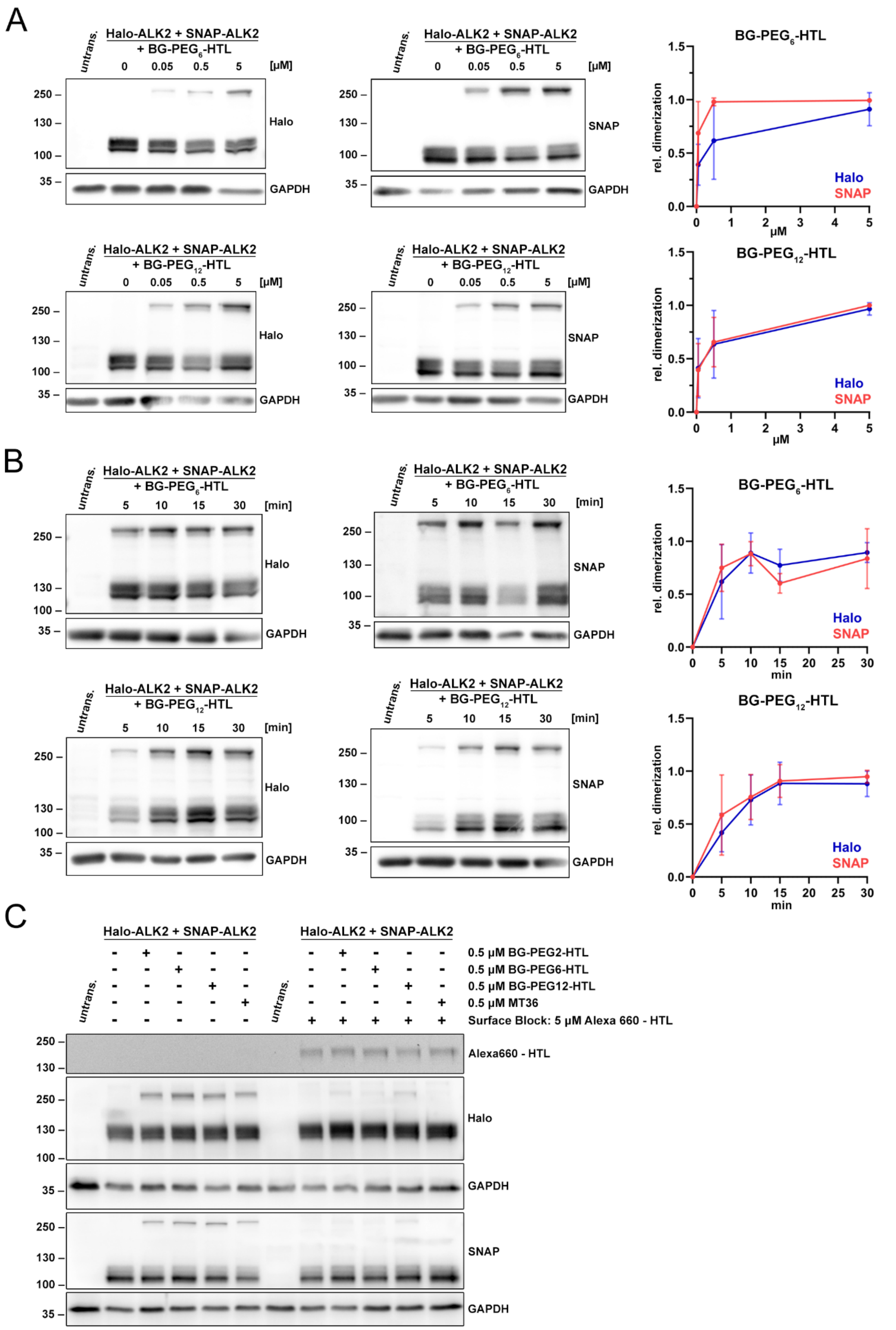

A
B
C
untrans.
Halo-ALK2 + SNAP-ALK2
+ BG-PEG6-HTL
+ BG-PEG12-HTL
0 0.05 0.5 5 [µM]
5 10 15 30 [min]
250 –
130 –
100 –
35 –
Halo
SNAP
GAPDH
BG-PEG6-HTL
BG-PEG12-HTL
rel. dimerization
µM
min
Halo
SNAP
0.5 µM BG-PEG2-HTL
0.5 µM BG-PEG6-HTL
0.5 µM BG-PEG12-HTL
0.5 µM MT36
Surface Block: 5 µM Alexa 660 - HTL
Alexa660 - HTL

Figure S2: Investigation of BG-PEG$_n$-HTL application and cell permeability. (**A**, **B**) (left) Immunoblot analysis of HEK293T cells transiently expressing Halo-ALK2 and SNAP-ALK2 stimulated with different BG-PEG6-HTL and BG-PEG12-HTL concentrations from 0.05 µM to 5 µM for 15 mins (**A**), or 0.5 µM from 5 to 30 min (**B**) with GAPDH as loading reference. (**A**, **B**) (right) Densitometric quantification of Halo/SNAP dimer bands relative to GAPDH levels, n = 3 independent experiments. (**C**) Investigation of permeability properties for BG-PEG$_n$-HTL and MT36. Fluorescence and Immunoblot analysis of HEK293T cells transiently expressing Halo-ALK2 and SNAP-ALK2 incubated first without and with 5 µM impermeable HTL-Alexa660 to block the Halo surface pool, followed by incubation with 0.5 µM BG-PEG$_n$-HTL and MT36 respectively.

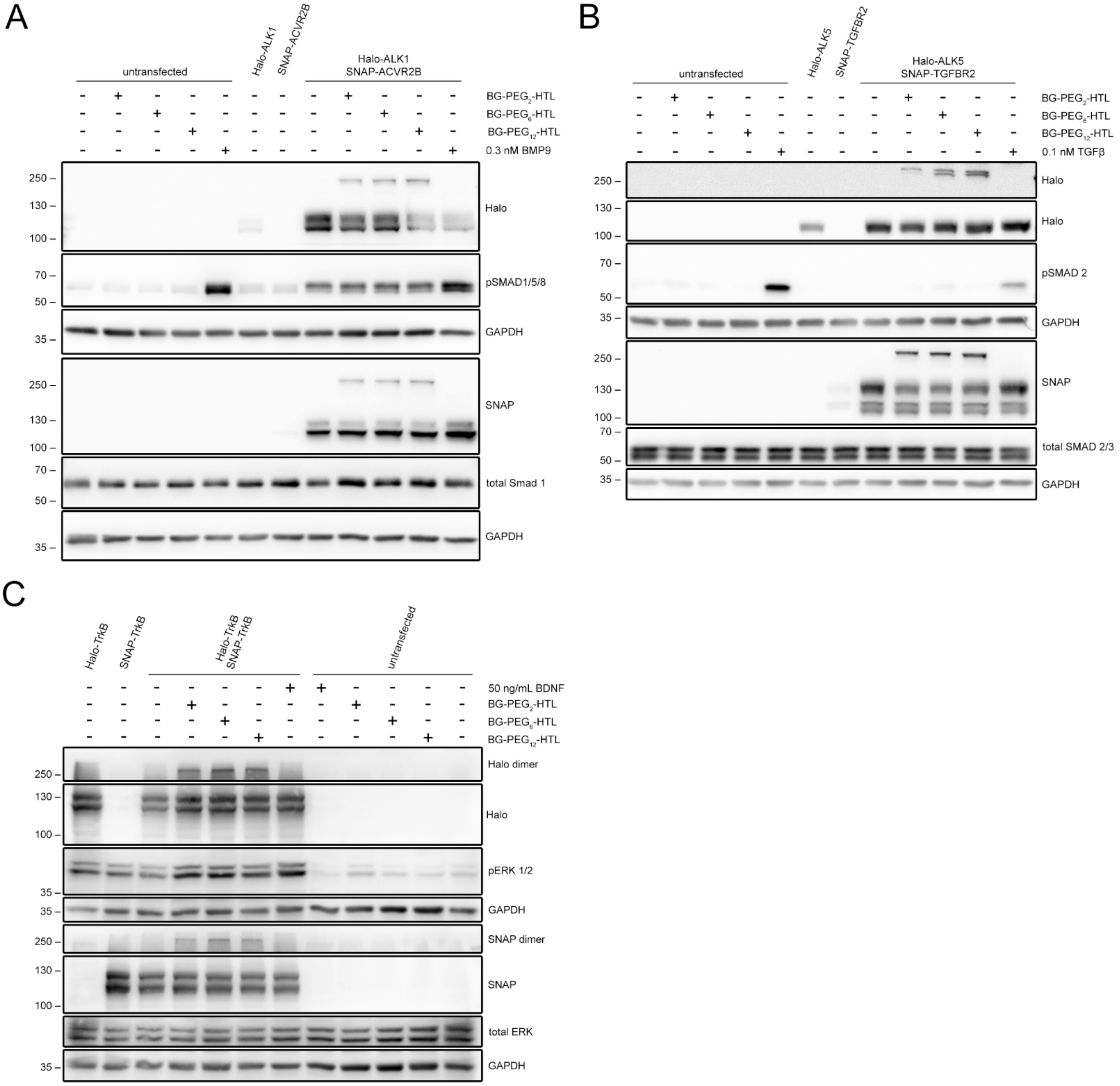

Figure S3: Full Immunoblots for Figure 2 B – D. (**A**) Immunoblot analysis of HEK293T cells transiently expressing Halo-ALK1, SNAP-ACVR2B or both stimulated with respective dimerizer or 0.3 nM BMP9 with pSMAD1/5 protein levels as a downstream signaling redout and total SMAD1 as control and GAPDH as loading reference. (**B**) Immunoblot analysis of HEK293T cells transiently expressing Halo-ALK5, SNAP-TGFBR2 or both stimulated with respective dimerizer or 0.2 nM TGFβ with pSMAD2/3 protein levels as downstream signaling readout and total SMAD1 as control and GAPDH as loading reference. (**C**) Immunoblot analysis of HEK293T cells transiently expressing Halo-TrkB, SNAP-TrkB or both stimulated with respective dimerizer or 50ng/mL BDNF with pERK1/2 protein levels as downstream signaling readout and total ERK1/2 as control and GAPDH as loading reference. In total three independent experiments for condition were performed and one exemplary Immunoblot is shown.

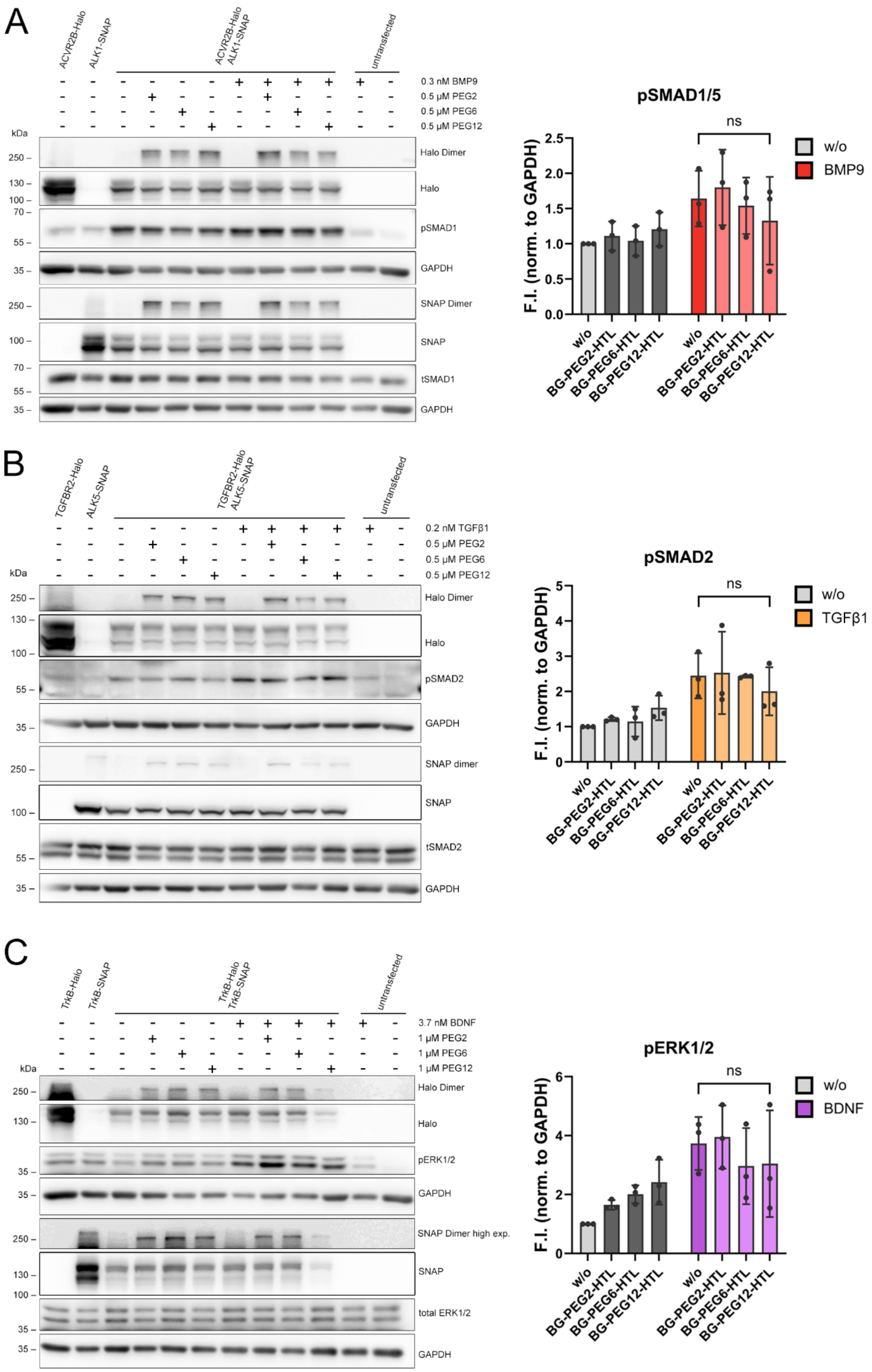

A
ACVR2B-Halo
ALK1-SNAP
ACVR2B-Halo ALK1-SNAP
untransfected
0.3 nM BMP9
0.5 µM PEG2
0.5 µM PEG6
0.5 µM PEG12
kDa
Halo Dimer
Halo
pSMAD1
GAPDH
SNAP Dimer
SNAP
tSMAD1
GAPDH
pSMAD1/5
F.I. (norm. to GAPDH)
ns
w/o
BMP9
BG-PEG2-HTL
BG-PEG6-HTL
BG-PEG12-HTL
B
TGFBR2-Halo
ALK5-SNAP
TGFBR2-Halo ALK5-SNAP
untransfected
0.2 nM TGFβ1
0.5 µM PEG2
0.5 µM PEG6
0.5 µM PEG12
Halo Dimer
Halo
pSMAD2
GAPDH
SNAP dimer
SNAP
tSMAD2
GAPDH
pSMAD2
TGFβ1
C
TrkB-Halo
TrkB-SNAP
TrkB-Halo TrkB-SNAP
untransfected
3.7 nM BDNF
1 µM PEG2
1 µM PEG6
1 µM PEG12
Halo Dimer
Halo
pERK1/2
GAPDH
SNAP Dimer high exp.
SNAP
total ERK1/2
GAPDH
pERK1/2
BDNF

Figure S4: Pre-dimerization of BMP/TGFβ and TrkB receptors does not enhance ligand-induced signaling. (A – C) left, HEK293T cells transiently expressing indicated receptor constructs pre-dimerized with respective dimerizer for 15 minutes at 4°C followed by addition of 0.3 nM BMP9 (**A**), 0.2 nM TGFβ1 **(B)**, 3,7 nM BDNF **(C)** at 37°C for 30 minutes. Immunoblot showing pSMAD1/5 and total SMAD1 **(A)**, pSMAD2 and total SMAD2 **(B)**, pERK1/2 and total ERK1/2 **(C)** as well as GAPDH as loading reference. **(A – C)** right, densitometric quantification of pSMAD1/5, pSMAD2/3 and pERK1/2 levels for unstimulated, stimulated with respective dimerizer or ligand relative to GAPDH are presented as fold induction (F.I.), n = 3 independent experiments. Significance was calculated within respective groups using two-way ANOVA and Dunnett's multiple comparisons test, ns = not significant.

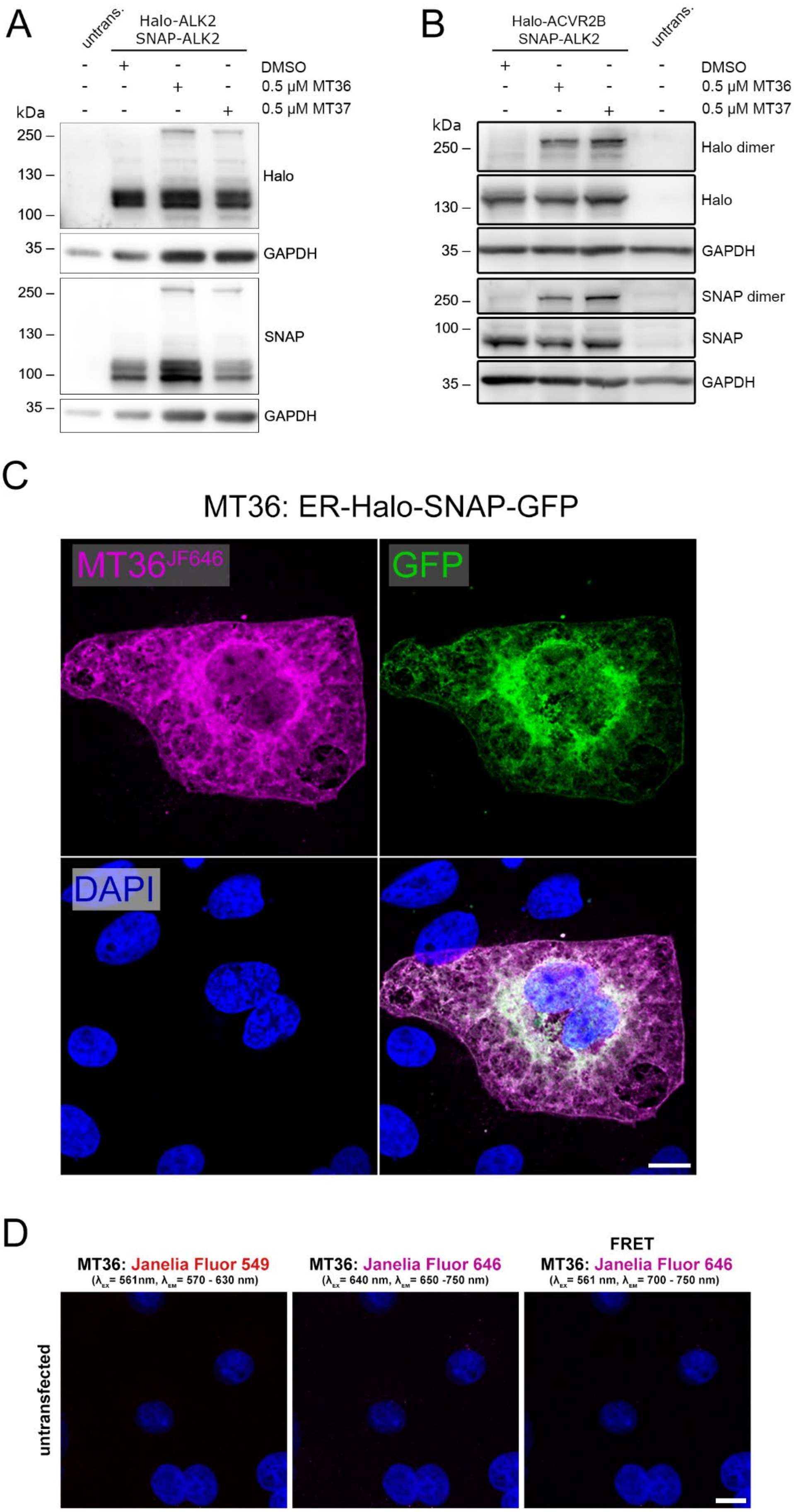
A
untrans.
Halo-ALK2
SNAP-ALK2
DMSO
0.5 µM MT36
0.5 µM MT37
kDa
250 –
130 –
100 –
Halo
35 –
GAPDH
SNAP
B
Halo-ACVR2B
SNAP-ALK2
Halo dimer
Halo
GAPDH
SNAP dimer
SNAP
C
MT36: ER-Halo-SNAP-GFP
MT36JF646
GFP
DAPI
D
MT36: Janelia Fluor 549
(λEX = 561nm, λEM = 570 - 630 nm)
MT36: Janelia Fluor 646
(λEX = 640 nm, λEM = 650 -750 nm)
FRET
MT36: Janelia Fluor 646
(λEX = 561 nm, λEM = 700 - 750 nm)
untransfected

Figure S5: Investigation of MT36 application and cell permeability. (**A**, **B**) Immunoblot analysis of HEK293T (A) and COS7 cells (B) transiently expressing Halo-ALK2/Halo-ACVR2B and SNAP-ALK2 stimulated with DMSO, 0.5 µM MT36 or MT37 and GAPDH as loading reference. (**C**) Representative confocal microscopy images of COS7 cells transiently expressing endoplasmic reticulum (ER) residing Halo-SNAP-GFP protein, incubated with MT36 at 4°C, showing specific intracellular binding capabilities of MT36. Scale bar ≙ 10 µm. (**D**) Complementary representative confocal microscopy images of non-transfected COS7-cells incubated with MT36 and DAPI staining of Fig. 3C. Scale bar ≙ 10 µm.

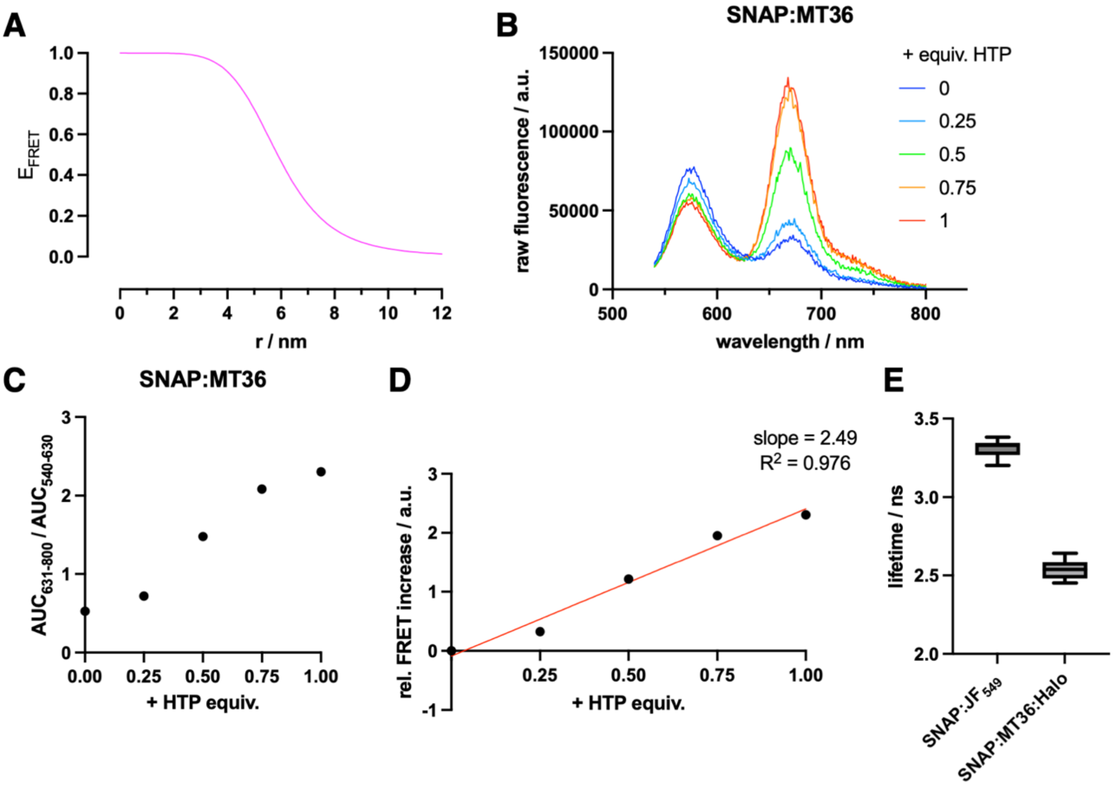

Figure S6: Investigation of MT36 FRET efficiency. (**A**) FRET efficiency plotted against distance r. To obtain the Förster radius between JF_{549} and JF_{646} by

$$R_0 \ = \ 0.211 \sqrt[6]{\kappa^2 n^{-4} Q_D J(\lambda)}$$

and

$$J(\lambda) \ = \int_o^\infty F_D(\lambda)\varepsilon_A(\lambda)\lambda^4 \, d\,\lambda \,/ \int_o^\infty F_D(\lambda) d\,\lambda$$

with $\kappa^2 \ = \ 0.66$ (assuming rotational freedom), $n \ = \ 1.33$ (refractive index of water), $Q_D \ = \ 0.81$, $\varepsilon_A \ = \ 140{,}000\, M^{-1}cm^{-2}$ (values from Grimm et al., Nat. Meth. 2015) and $J(\lambda)$ = 2.68 ×10^{15} $M^{-1}cm^{-1}nm^4$ to obtain

$$R_0 \ = \ 5.85\, nm$$

and plotting FRET efficiency by

$$E_{FRET} \ = \ \frac{1}{1 \ + \left(\frac{r}{R_0}\right)^6}$$

(**B**) Emission scans of SNAP:MT36 by adding 0, 0.25, 0.5 0.75 and 1.0 equivalents of HaloTag protein. (**C**) Ratio of area under curve for the donor (540 - 630 nm) and of the acceptor (631 - 800 nm) from (B), and free SNAP:MT36 subtracted for linear fitting (**D**) to give a 'degree of dimerization'. (**E**) Fluorescence lifetime measurement of SNAP:JF_{549} and SNAP:MT36:Halo was performed to calculate

$$E_{av.FRET} \ = \ 1 \ - \ \frac{\tau_{D-A}}{\tau_D} = 1 \ - \ \frac{2.538\, ns}{3.306\, ns} \ = \ 23\%$$

See also Material and Methods for a brief discussion.

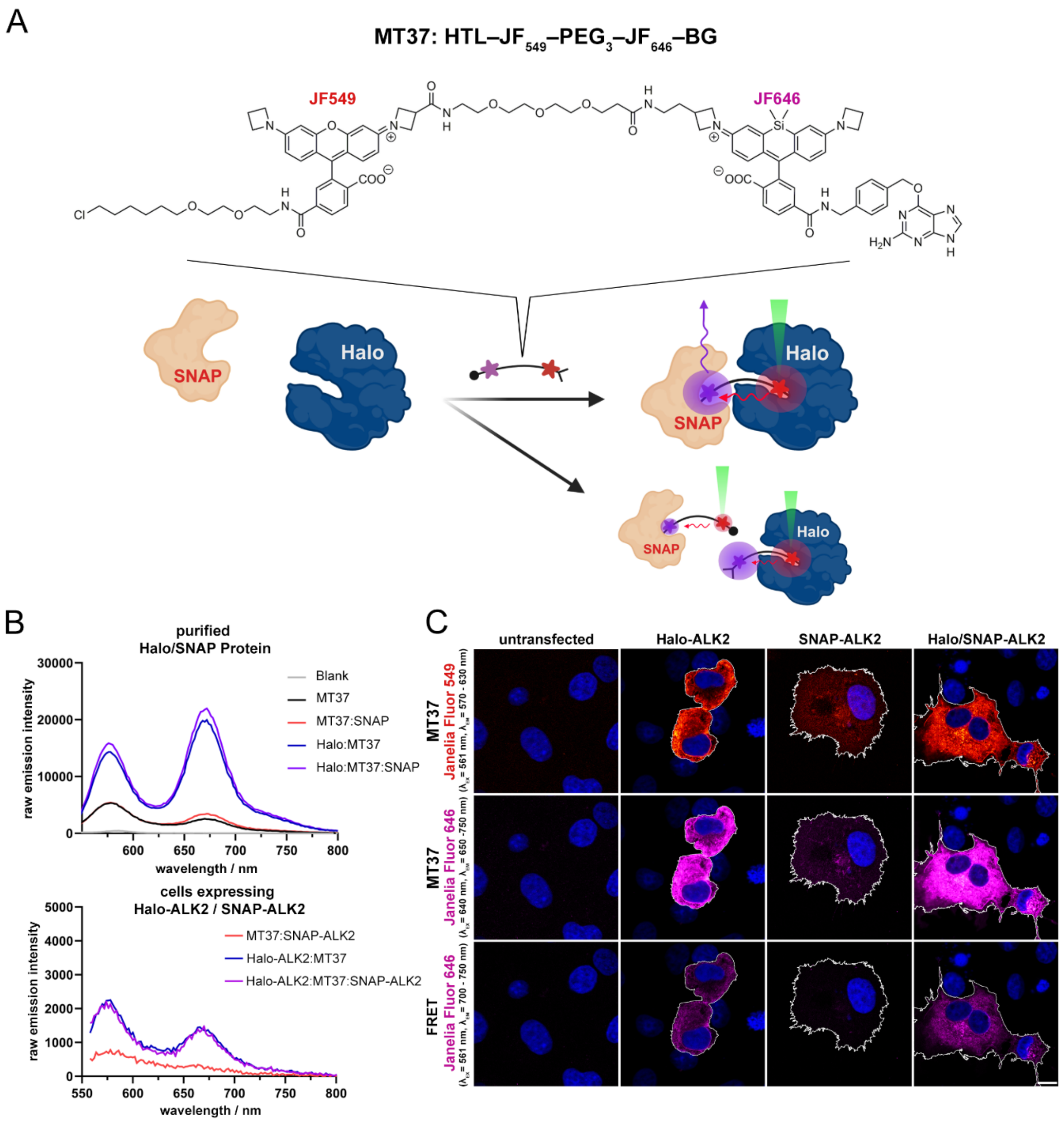

Figure S7: MT37 - a fluorescent dimerizer tool inducing but not confirming protein proximity. (**A**) Structure of fluorescent dimerizer MT37 HTL–JF_{549}–PEG_3–JF_{646}–BG. Sketch below shows working principle of MT37. (**B**) Emission FRET spectra measured in raw emission intensity (λex= 550 nm, λem= 560 – 800 nm) of unbound, partially and fully bound MT37 dimerizer to soluble purified Halo and/or SNAP proteins (up), or cells transiently expressing Halo-ALK2 and/or SNAP-ALK2 (below). Raw emission intensity values were measured as triplicates and for cell measurement as quadruplicates. (**C**) Representative confocal microscopy images of COS7-cells transiently expressing ALK2-Halo and/or ALK2-SNAP incubated with MT37 and DAPI staining. Images are acquired in red channel for JF_{549} emission (λex= 561 nm, λem= 570 – 630 nm), in infrared channel for JF_{646} emission (λex= 640 nm, λem= 650 – 750 nm) or FRET channel (excitation of JF_{549}, λex= 561 nm, λem= 700 – 750 nm). Scale bar ≙ 10 µm.

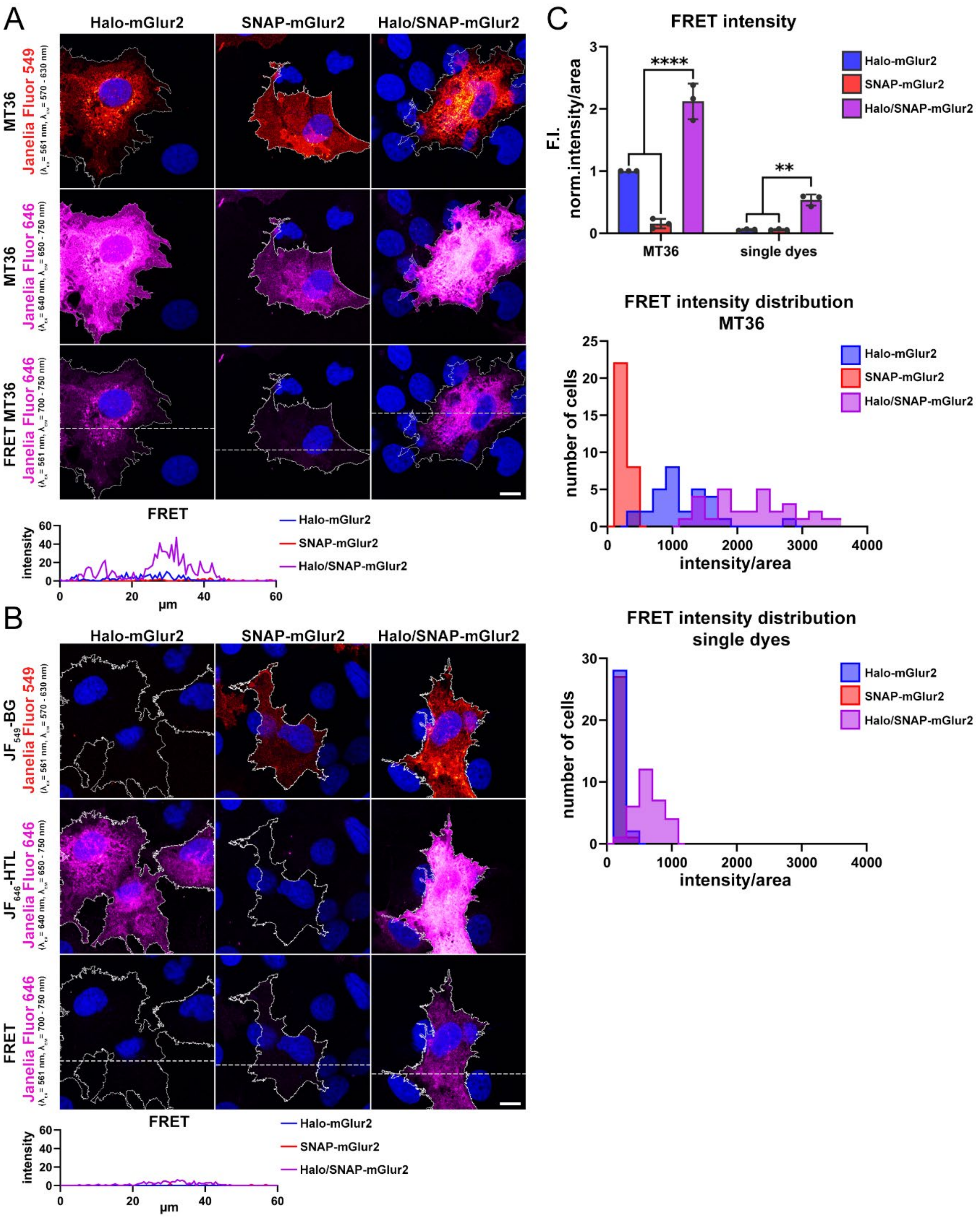

A
Halo-mGlur2
SNAP-mGlur2
Halo/SNAP-mGlur2
MT36
Janelia Fluor 549
FRET MT36
Janelia Fluor 646
FRET
intensity
µm
B
JF549-BG
JF646-HTL
C
FRET intensity
F.I. norm.intensity/area
****
**
MT36
single dyes
FRET intensity distribution MT36
number of cells
intensity/area
FRET intensity distribution single dyes

Figure S8: FRET based validation of mGluR2 dimerization. (A) Representative confocal microscopy images of COS7-cells transiently expressing Halo-mGluR2 and/or SNAP-mGluR2 incubated with MT36 and DAPI. Images are acquired in red channel for JF_{549} emission (λex= 561 nm, λem= 570 – 630 nm), in infrared channel for JF_{646} emission (λex= 640 nm, λem= 650 – 750 nm) or FRET channel (excitation of JF_{549}, λex= 561 nm, λem= 700 – 750 nm). A linescan was performed to assess fluorescence intensity within the FRET channel, and its path is highlighted as dashed lines. Scale bar ≙ 10 µm. (**B upper & C**) Mean fluorescence FRET & pERK1/2 intensities per cell area were normalized to Halo-mGluR2. Data shown as Fold induction (F.I.). (**B lower**) grouped area normalized mean FRET intensity values of each cell (n=3 with 10 cells each). Significance was calculated within respective groups using two-way ANOVA and Šídák's multiple comparisons test, ****p < 0.0001. n=3 independent experiments with 10 cells per condition.

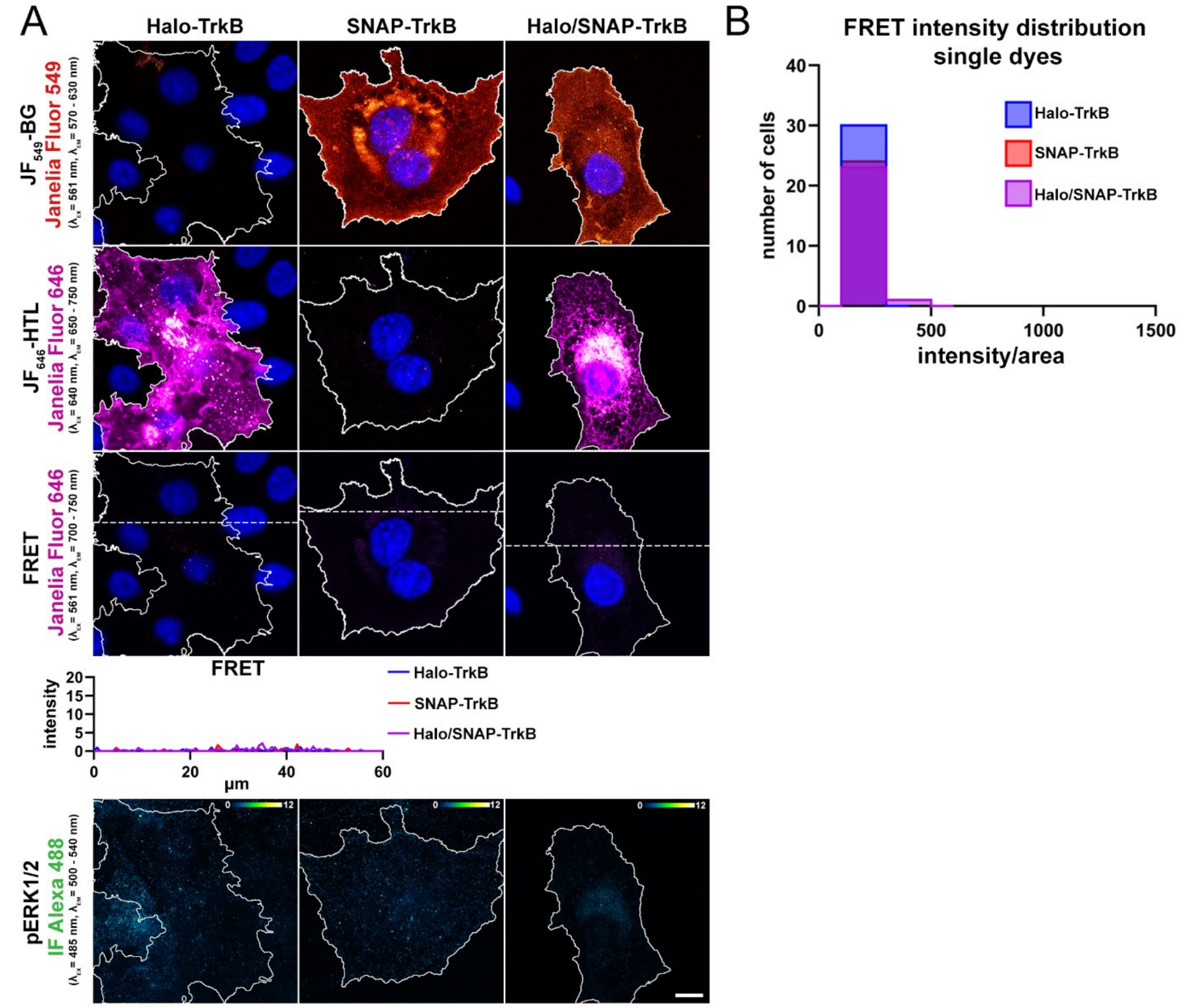

Figure S9: Complementary control condition for FRET based validation of TrkB dimerization and pERK1/2 induction. (**A**) Representative confocal microscopy images of COS7-cells transiently expressing Halo-TrkB and/or SNAP-TrkB incubated with JF_{549}-BG and JF_{646}-HTL and DAPI. Images are acquired in red channel for JF_{549} emission (λex= 561 nm, λem= 570 – 630 nm), in infrared channel for JF_{646} emission (λex= 640 nm, λem= 650 – 750 nm), FRET channel (excitation of JF_{549}, λex= 561 nm, λem= 700 – 750 nm), green channel (excitation of Alexa 488, λex= 485 nm, λem= 500 – 540 nm). A linescan was performed to assess fluorescence intensity within the FRET channel, and its path is highlighted as dashed lines. Scale bar ≙ 10 µm. (**B**) grouped area normalized mean FRET intensity values of each cell (n=3 with 10 cells each).

Supplementary Table

Table S1: Overexpression DNA constructs

Plasmid Name	Description	Backbone	Resistance
Halo-ALK2	full length human ALK2 with N-terminal Halo-tag	pcDNA3.1	Ampicillin
SNAP-ALK2	full length human ALK2 with N-terminal SNAP-tag	pcDNA3.1	Ampicillin
Halo-ALK1	full length human ALK1 with N-terminal Halo-tag	pcDNA3.1	Ampicillin
SNAP-ACVR2B	full length human ACVR2B with N-terminal SNAP-tag	pcDNA3.1	Ampicillin
Halo-ALK5	full length human ALK5 with N-terminal Halo-tag	pcDNA3.1	Ampicillin
SNAP-TGFBR2	full length human TGFBR2 with N-terminal SNAP-tag	pcDNA3.1	Ampicillin
Halo-ALK4	full length human ALK4 with N-terminal Halo-tag	pcDNA3.1	Ampicillin
SNAP-ACVR2A	full length human ACVR2A with N-terminal SNAP-tag	pcDNA3.1	Ampicillin
Halo-ALK3	full length human ALK3 with N-terminal Halo-tag	pcDNA3.1	Ampicillin
SNAP-ALK3	full length human ALK3 with N-terminal SNAP-tag	pcDNA3.1	Ampicillin
SNAP-BMPR2	full length human BMPR2 with N-terminal SNAP-tag	pcDNA3.1	Ampicillin
Halo-TrkB	full length human TrkB with N-terminal Halo-tag gift from Joshua Levitz, Weill Cornell Medicine Graduate School of Medical Sciences	pcDNA3.1	Ampicillin
SNAP-TrkB	full length human TrkB with N-terminal SNAP-tag gift from Joshua Levitz, Weill Cornell Medicine Graduate School of Medical Sciences	pcDNA3.1	Ampicillin
Halo-mGluR2	full length human mGluR2 with N-terminal Halo-tag gift from Joshua Levitz, Weill Cornell Medicine Graduate School of Medical Sciences	pRK5	Ampicillin
SNAP-mGluR2	full length human mGluR2 with N-terminal SNAP-tag gift from Joshua Levitz, Weill Cornell Medicine Graduate School of Medical Sciences	pRK5	Ampicillin

130-pcDNA5-FRT-ER-Halo-SNAP-meGFP	ER localized Halo-Tag linked to SNAP-Tag linked to meGFP Protein gift from Johannes Broichhagen FMP-Berlin/Buch	pcDNA5	Ampicillin

Synthesis

General chemistry

Chemicals and solvents were purchased from Merck (Merck group, Germany), TCI (Tokyo chemical industry CO., LTD., Japan) and Acros Organics (Thermo Fisher scientific, USA) and used without further purification. Dry solvents were purchased from Acros Organics (Thermo Fisher scientific, USA). Amino acids and resins for SPPS were purchased from Novabiochem (Merck, USA) or Iris Biotech GmbH (Germany).

UPLC-UV/Vis for purity assessment was performed on an Agilent 1260 Infinity II LC System equipped with Agilent SB-C18 column (1.8 µm, 2.1 × 50 mm). Buffer A: 0.1% FA in H_2O Buffer B: 0.1% FA acetonitrile. The typical gradient was from 10% B for 1.0 min → gradient to 95% B over 5 min → 95% B for 1.0 min with 0.45 mL/min flow or from 30% B for 1.0 min → gradient to 95% B over 5 min. Retention times (t_R) are given in minutes (min). Chromatograms were imported into Graphpad Prism 8 and purity was determined by calculating AUC ratios.

Preparative or semi-preparative HPLC was performed on an Agilent 1260 Infinity II LC System equipped with columns as followed: preparative column –Reprospher 100 C18 columns (10 µm: 50 x 30 mm at 20 mL/min flow rate; semi-preparative column – 5 µm: 250 x 10 mm at 4 mL/min flow rate. Eluents A (0.1% TFA in H_2O) and B (0.1% TFA in MeCN) were applied as a linear gradient. Peak detection was performed at maximal absorbance wavelength.

For HRMS, samples were analyzed on Orbitrap Fusion mass spectrometer (Thermo Fisher Scientific). MS scans were acquired in a range of 350 to 1500 m/z. MS1 scans were acquired in the Orbitrap with a mass resolution of 120,000 with an AGC target value of 4e5 and 50 ms injection time. MS2 scans were acquired in the ion trap with an AGC target value of 1e4 and 35 ms injection time. Precursor ions with charge states 2-4 were isolated with an isolation window of 1.6 m/z and 40 sec dynamic exclusion. Precursor ions were fragmented using higher-energy collisional dissociation (HCD) with 30% normalized collision energy.

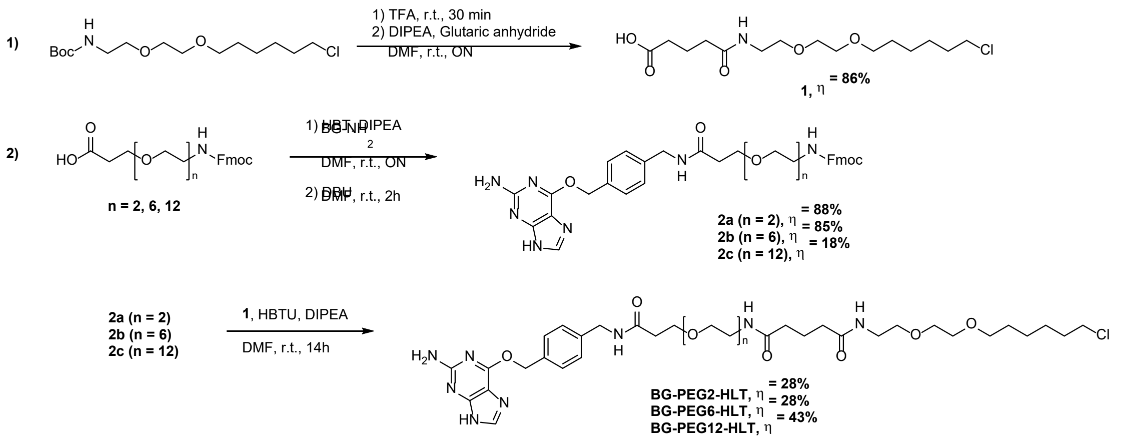

Scheme S1: Synthesis of BG-PEG2-HTL, BG-PEG6-HTL, BG-PEG12-HTL

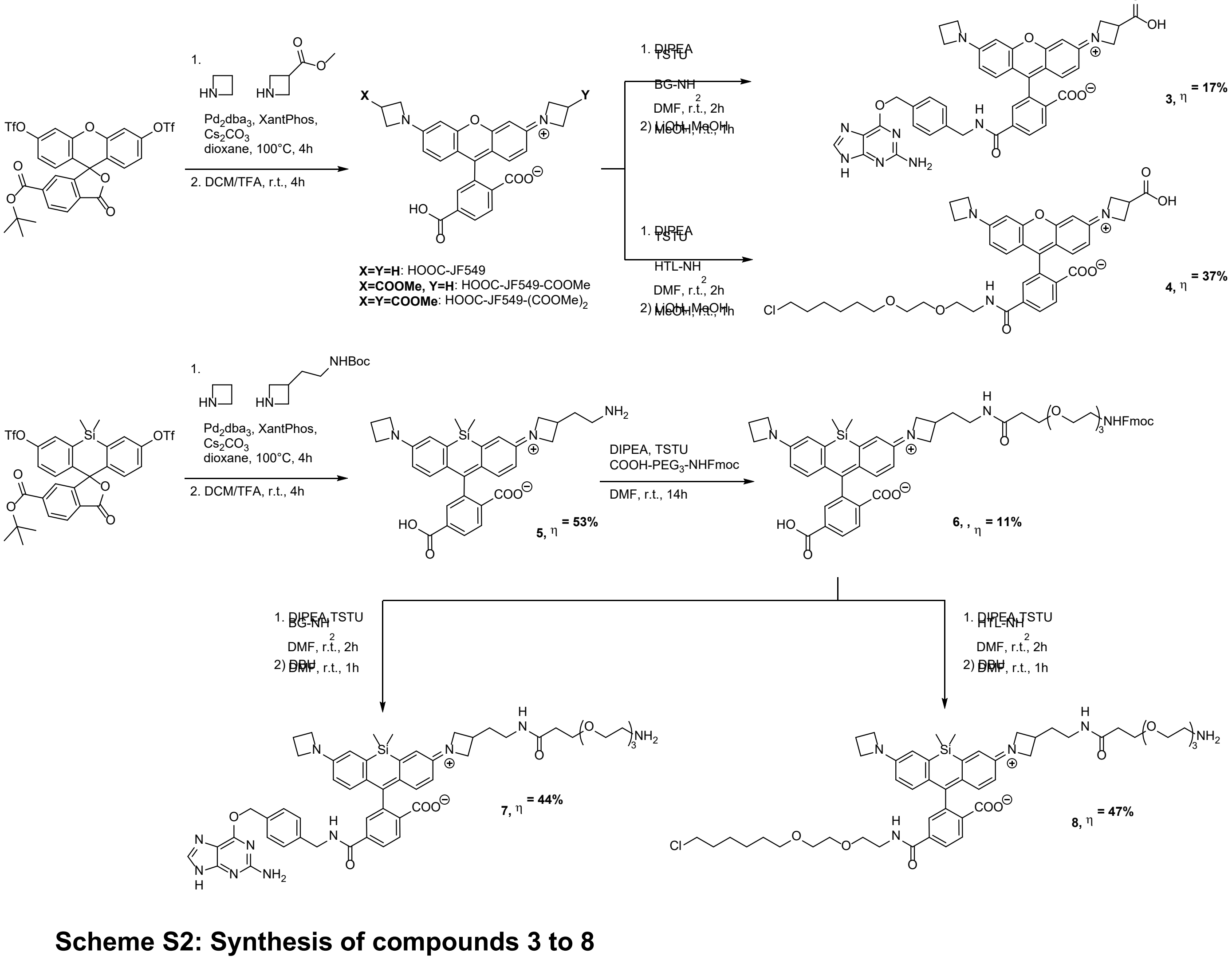

Scheme S2: Synthesis of compounds 3 to 8

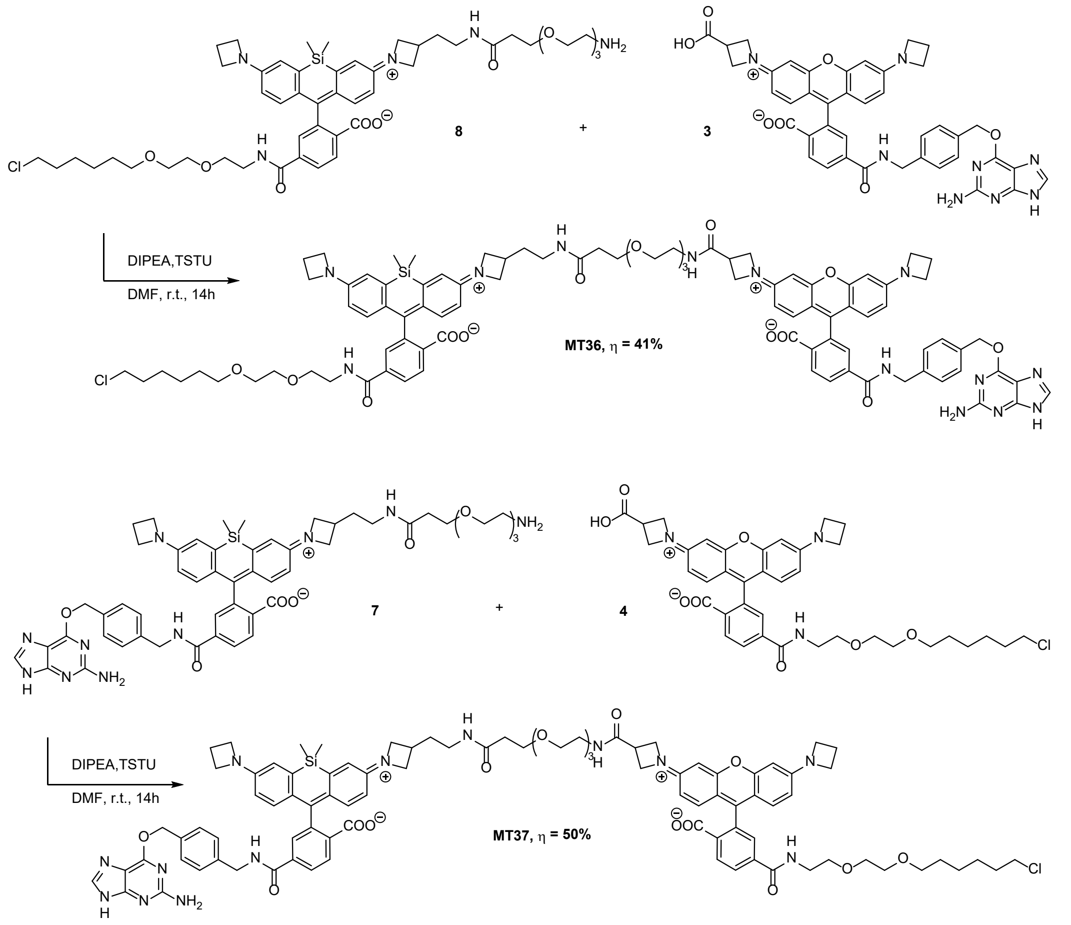

Scheme S3: Synthesis of compounds MT36 and MT37

General procedure A for HOOC-PEG$_n$-NH-Fmoc to BG coupling

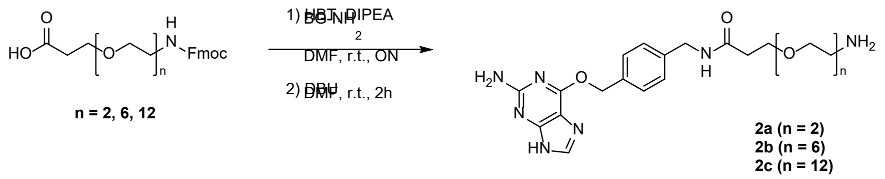

A 4 mL glass vial was charged with HOOC-PEG$_n$-NH-Fmoc (n = 2, 6, 12; 1.0 equiv.) dissolved in 1 mL DMF, before DIPEA (4.0 equiv.) and HBTU (1.2 equiv.) were added successively. The reaction mixture was stirred for 30 min then BG-NH$_2$ (1.2 equiv.) was added and the reaction mixture was allowed to incubate until complete conversion was observed. DBU was added (deprotection in 2% DBU for 1h). The reaction was quenched with 200 µL HOAc, diluted with H_2O:MeCN:AcOH (25:25:1) and subjected to RP-HPLC (MeCN:H_2O+0.1% TFA = 10:90 to 90:10 over 60 minutes) to obtain the desired product after lyophilization.

General procedure B for BG-PEG$_n$-NH$_2$ to HTL coupling

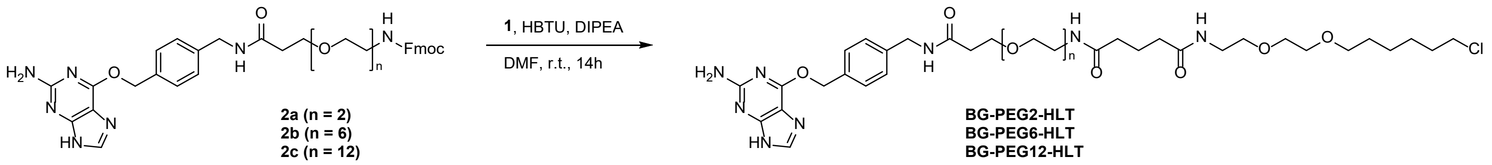

An Eppendorf was charged with **1** (1.2 equiv.) dissolved in 150µL DMF, DIPEA (8.0 equiv.) and TSTU (1.5 equiv.) were added successively. After 15 minutes incubation, a solution of BG-PEG$_n$-NH$_2$ (1.0 equiv.) in 200 µL DMF was added. The reaction mixture was stirred at r.t. during 14h, before it was quenched with 10 µL HOAc, diluted with H_2O:MeCN:AcOH (25:25:1) and subjected to RP-HPLC (MeCN:H_2O+0.1% TFA = 10:90 to 90:10 over 60 minutes) to obtain the desired product after lyophilization.

5-((2-(2-((6-Chlorohexyl)oxy)ethoxy)ethyl)amino)-5-oxopentanoic acid 1

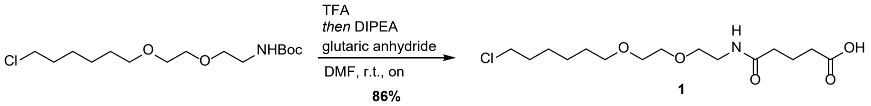

A 4 mL glass vial was charged with HTL-NHBoc (10.0 mg, 30.9 µmol, 1.0 equiv.) and 100 µL neat Trifluoroacetic acid (TFA) was added. The reaction mixture was stirred to ensure homogeneity and allowed to incubate for 30 min, before all volatiles were removed under a gentle stream of nitrogen. The residue was taken up in 200 µL DMF and DIPEA (21 µL, 124 µmol, 4.0 equiv.) was added, before glutaric anhydride (4.2 mg, 37.1 µmol, 1.2 equiv.) was added in one portion. The reaction mixture was incubated overnight before it was quenched with 100 µL HOAc, diluted with H_2O:MeCN:AcOH (25:25:1) and subjected to RP-HPLC (MeCN:H_2O+0.1% TFA = 10:90 to 90:10 over 60 minutes) to obtain 9.0 mg (86%) of the desired product after lyophilization as a colorless oil.

^{1}H NMR (600 MHz, $CDCl_3$) δ 6.37 (t, J = 5.5 Hz, 1H, NH), 3.64 – 3.59 (m, 2H), 3.60 – 3.57 (m, 2H), 3.56 (t, J = 5.0 Hz, 2H), 3.53 (t, J = 6.7 Hz, 2H), 3.50 – 3.43 (m, 4H), 2.41 (t, J = 7.0 Hz, 2H), 2.31 (t, J = 7.3 Hz, 2H), 1.97 (p, J = 7.1 Hz, 2H), 1.77 (dt, J = 14.6, 6.8 Hz, 2H), 1.61 (p, J = 6.9 Hz, 2H), 1.49 – 1.41 (m, 2H), 1.41 – 1.33 (m, 2H).

^{13}C NMR (151 MHz, $CDCl_3$) δ 176.5 (C), 173.0 (C), 71.5 (CH_2-O), 70.3 (CH_2-O), 70.1 (CH_2-O), 69.9 (CH_2-O), 45.1 (CH_2-Cl), 39.4 (CH_2-NH), 35.3 (CH_2), 33.0 (CH_2), 32.6 (CH_2), 29.4 (CH_2), 26.8 (CH_2), 25.5 (CH_2), 20.8 (CH_2).

^{19}F NMR (564 MHz, $CDCl_3$) δ -76.0.

LCMS (ESI): calc. for $C_{15}H_{29}ClNO_5$ $[M+H]^+$: 338.2, found: 338.2.

HRMS (ESI): calc. for $C_{15}H_{29}ClNO_5$ $[M+H]^+$: 338.1729, found: 338.1761.

_N_-(4-(((2-Amino-9_H_-purin-6-yl)oxy)methyl)benzyl)-2-(2-(2-aminoethoxy)ethoxy)acetamide 2a

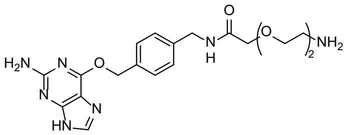

Prepared according to general procedure A.

HOOC-PEG_2-NHFmoc: (10.0 mg, 28 µmol), yield: 15.7 mg (88%). White solid.

^{1}H NMR (600 MHz, MeOD-d4) δ 8.24 (s, 1H), 7.52 (d, J = 8.0 Hz, 2H), 7.35 (d, J = 8.0 Hz, 2H), 5.62 (s, 2H), 4.45 (s, 2H), 4.08 (s, 2H), 3.75 – 3.70 (m, 4H), 3.70 – 3.64 (m, 2H), 3.12 – 3.04 (m, 2H).

^{13}C NMR (151 MHz, MeOD-d4) δ 172.6 (C), 161.2 (C), 154.2 (C), 142.8 (CH), 140.6 (C), 135.8 (C), 130.2 (2 × CH), 128.9 (2 × CH), 71.9 (CH_2-O), 71.4 (CH_2-O), 71.2 (CH_2-O), 70.4 (O-CH_2-CONH), 67.9 (CH_2-O), 43.4 (CH_2-NH), 40.6 (CH_2-NH_2). (two Cq are missing).

^{19}F NMR (564 MHz, MeOD-d4) δ -77.1.

HRMS (ESI): calc. for $C_{19}H_{26}N_7O_4$ $[M+H]^+$: 416.2041, found: 416.2044.

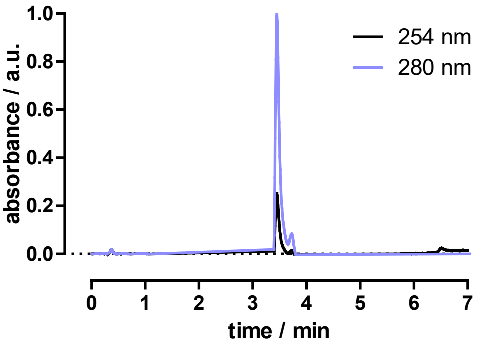

1-Amino-*N*-(3-(((2-amino-9*H*-purin-6-yl)oxy)methyl)benzyl)-3,6,9,12,15,18-hexaoxa-henicosan-21-amide 2b

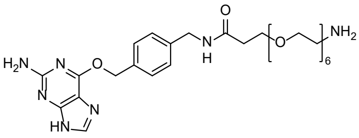

Prepared according to general procedure A.

HOOC-PEG6-NHFmoc: (16.1 mg, 57.5 µmol), yield 16.9 mg (85%). White solid.

^{1}H NMR (600 MHz, MeOD-d4) δ 8.11 (s, 1H), 7.51 (d, J = 8.2 Hz, 2H), 7.33 (d, J = 8.1 Hz, 2H), 5.61 (s, 2H), 4.41 (d, J = 5.0 Hz, 2H), 3.76 (t, J = 5.9 Hz, 2H), 3.73 – 3.69 (m, 2H), 3.67 – 3.64 (m, 2H), 3.64 – 3.57 (m, 18H), 3.10 – 3.06 (m, 2H), 2.53 (t, J = 5.9 Hz, 2H).

^{13}C NMR (151 MHz, MeOD-d4) δ 174.0 (C), 161.3 (C), 159.9 (C), 155.2 (C), 141.9 (CH), 140.5 (C), 136.1 (C), 130.0 (2 × CH), 128.6 (2 × CH), 71.4 (CH_2-O), 71.4 (CH_2-O), 71.3 (CH_2-O), 71.2 (2 × CH_2-O), 71.1 (CH_2-O), 71.0 (CH_2-O), 70.9 (CH_2-O), 70.7 (CH_2-O), 69.7 (CH_2-O), 68.3 (CH_2-O), 67.9 (CH_2-O), 43.8 (CH_2-NH), 40.6 (CH_2-CONH), 37.4 (CH_2-NH_2). (One Cq is missing).

^{19}F NMR (564 MHz, MeOD-d4) δ -76.9.

HRMS (ESI): calc. for $C_{28}H_{43}N_7O_8$ $[M+H]^+$: 606.3246, found: 606.3264

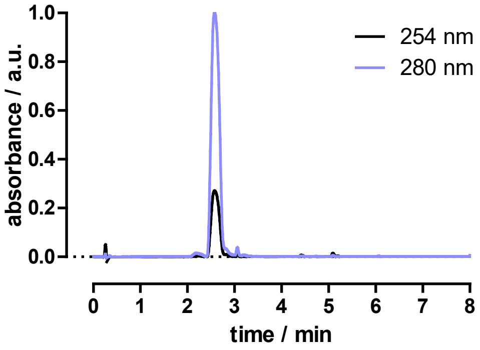

1-Amino-*N*-(3-(((2-amino-9*H*-purin-6-yl)oxy)methyl)benzyl)-3,6,9,12,15,18,21,24,27,30, 33,36-dodecaoxanonatriacontan-39-amide 2c

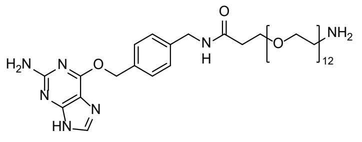

Prepared according to general procedure A.

HOOC-PEG$_{12}$-NHFmoc: (38.3 mg, 45.8 µmol), yield 7.2 mg (18%).

^{1}**H NMR** (600 MHz, MeOD-d4) δ 8.26 (s, 1H), 7.51 (d, J = 8.0 Hz, 2H), 7.35 (d, J = 8.0 Hz, 2H), 5.63 (s, 2H), 4.41 (s, 2H), 3.78 – 3.74 (m, 4H), 3.71 – 3.56 (m, 38H), 3.21 – 3.14 (m, 2H), 2.50 (t, J = 6.1 Hz, 2H).

^{13}**C NMR** (151 MHz, MeOD-d4) δ 173.7 (C), 161.2 (C), 154.9 (C), 142.4 (CH), 140.7 (C), 135.9 (C), 130.0 (2 × CH), 128.7 (2 × CH), 71.5 (CH_2-O), 71.5 (CH_2-O), 71.3 (CH_2-O), 71.3 (CH_2-O), 71.2 (CH_2-O), 71.1 (CH_2-O), 71.0 (CH_2-O), 70.7 (CH_2-O), 70.1 (CH_2-O), 68.3 (CH_2-O), 67.9 (CH_2-O), 43.8 (CH_2-NH), 40.7 (CH_2-CONH), 37.7 (CH_2-NH_2). (two Cq are missing).

^{19}**F NMR** (564 MHz, MeOD-d4) δ -77.0.

HRMS (ESI): calc. for $C_{28}H_{43}N_7O_8$ $[M+H]^+$: 870.4819, found: 869.4899.

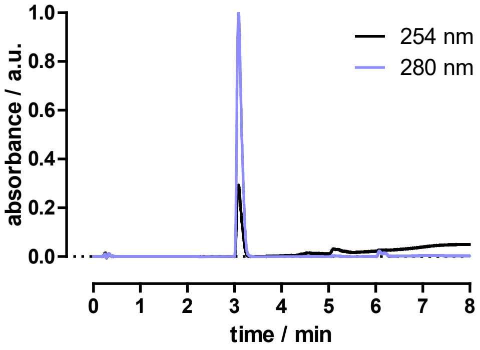

***N*^1-(2-(2-(2-((4-(((2-Amino-9*H*-purin-6-yl)oxy)methyl)benzyl)amino)-2-oxoethoxy)ethoxy)ethyl)-*N*^5-(2-(2-((6-chlorohexyl)oxy)ethoxy)ethyl)glutaramide**

BG-PEG2-HTL

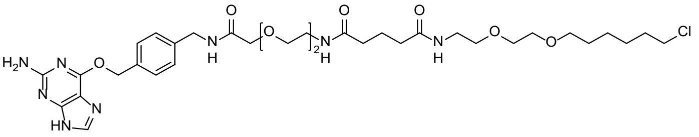

Prepared according to general procedure B.

BG-PEG2-NH_2 **2a** (3.1 mg, 7.40 µmol), yield 1.5 mg (28%).

^{1}H NMR (600 MHz, MeOD-d4) δ 8.17 (s, 1H), 7.51 (d, J = 8.0 Hz, 2H), 7.35 (d, J = 8.1 Hz, 2H), 5.61 (s, 2H), 4.46 (s, 2H), 4.05 (s, 2H), 3.72 – 3.68 (m, 2H), 3.66 – 3.63 (m, 2H), 3.60 – 3.58 (m, 2H), 3.58 – 3.55 (m, 2H), 3.55 – 3.51 (m, 4H), 3.50 (t, J = 5.6 Hz, 2H), 3.47 (t, J = 6.6 Hz, 2H), 3.34 (t, J = 5.5 Hz, 2H), 3.28 (t, J = 5.5 Hz, 2H), 2.18 (q, J = 7.7 Hz, 4H), 1.85 (p, J = 7.5 Hz, 2H), 1.75 (q, J = 6.1 Hz, 2H), 1.58 (q, J = 7.0 Hz, 2H), 1.48 – 1.42 (m, 2H), 1.40 – 1.36 (m, 2H).

^{13}C NMR (151 MHz, MeOD-d4) δ 175.5 (C), 175.4 (C), 172.7 (C), 161.2 (C), 142.3 (CH), 140.4 (C), 136.1 (C), 130.1 (CH), 128.9 (CH), 72.2 (CH_2), 72.1 (CH_2), 71.4 (CH_2), 71.3 (CH_2), 71.2 (CH_2), 71.2 (CH_2), 70.6 (CH_2), 70.1 (CH_2-O), 45.7 (CH_2), 43.3 (CH_2-NH), 40.4 (CH_2), 40.2 (CH_2), 36.1 (CH_2), 36.1 (CH_2), 33.7 (CH_2), 30.5 (CH_2), 27.7 (CH_2), 26.5 (CH_2), 23.2 (CH_2). (two Cq are missing).

^{19}F NMR (564 MHz, MeOD-d4) δ -77.0.

HRMS (ESI): calc. for $C_{34}H_{52}ClN_8O_8$ $[M+H]^+$: 735.3592, found: 735.3616.

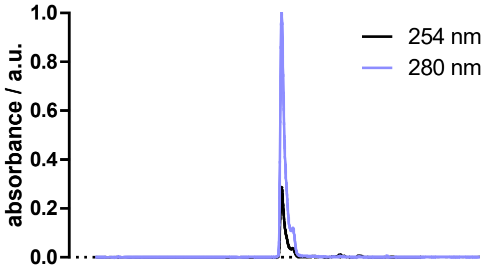

N^1-(1-(4-(((2-Amino-9*H*-purin-6-yl)oxy)methyl)phenyl)-3-oxo-6,9,12,15,18,21-hexaoxa-2-azatricosan-23-yl)-N^5-(2-(2-((6-chlorohexyl)oxy)ethoxy)ethyl) glutaramide BG-PEG6-HTL

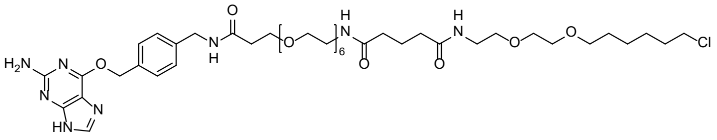

Prepared according to general procedure B.

BG-PEG6-NH_2 **2b** (4.5 mg, 7.40 µmol), yield 1.9 mg (28%).

^{1}H NMR (600 MHz, MeOD-d4) δ 8.16 (s, 1H), 7.50 (d, *J* = 8.1 Hz, 2H), 7.34 (d, *J* = 7.9 Hz, 2H), 5.61 (s, 2H), 4.41 (s, 2H), 3.75 (t, *J* = 6.0 Hz, 2H), 3.64 – 3.51 (m, 30H), 3.47 (t, *J* = 6.6 Hz, 2H), 3.35 (q, *J* = 5.8 Hz, 4H), 2.50 (t, *J* = 6.0 Hz, 2H), 2.21 (t, *J* = 8.3 Hz, 4H), 1.88 (p, *J* = 7.5 Hz, 2H), 1.76 (p, *J* = 6.8 Hz, 2H), 1.58 (p, *J* = 6.8 Hz, 2H), 1.49 – 1.43 (m, 2H), 1.41 – 1.36 (m, 2H).

^{13}C NMR (151 MHz, MeOD-d4) δ 175.4 (C), 174.0 (C), 161.2 (C), 155.0 (C), 142.3 (CH), 140.6 (C), 135.9 (C), 130.0 (CH), 128.7 (CH), 72.2 (CH_2), 71.5 (CH_2), 71.5 (CH_2), 71.5 (CH_2), 71.4 (CH_2), 71.3 (CH_2), 71.2 (CH_2), 70.6 (CH_2), 70.5 (CH_2), 70.0 (CH_2), 68.3 (CH_2), 45.7 (CH_2), 43.8 (CH_2), 40.4 (CH_2), 37.8 (CH_2), 36.1 (CH_2), 33.7 (CH_2), 30.5 (CH_2), 27.7 (CH_2), 26.5 (CH_2), 23.2 (CH_2).

^{19}F NMR (564 MHz, MeOD-d4) δ -77.0.

HRMS (ESI): calc. for $C_{43}H_{71}ClN_8O_{12}$ $[M+2H]^+$: 463.2435, found: 463.2377.

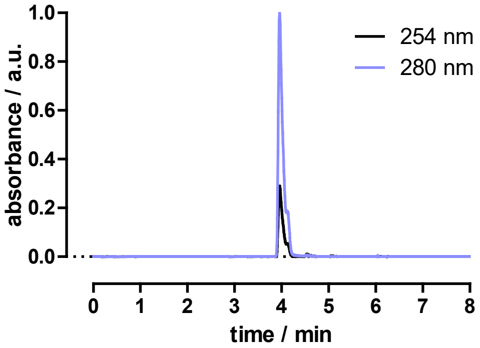

***N*[1]-(1-(4-(((2-amino-9*H*-purin-6-yl)oxy)methyl)phenyl)-3-oxo-6,9,12,15,18,21,24,27,30,33,36,39-dodecaoxa-2-azahentetracontan-41-yl)-*N*[5]-(2-(2-((6-chlorohexyl)oxy)ethoxy)ethyl) glutaramide BG-PEG12-HTL**

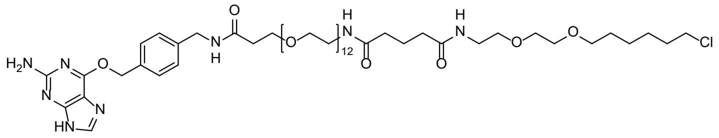

Prepared according to general procedure B.

BG-PEG12-NH2 **2c** (6.5 mg, 7.40 µmol), yield 3.8 mg (43%).

^{1}H NMR (600 MHz, MeOD-d4) δ 8.21 (s, 1H), 7.51 (d, *J* = 7.9 Hz, 2H), 7.35 (d, *J* = 7.9 Hz, 2H), 5.62 (s, 2H), 4.41 (s, 2H), 3.75 (t, *J* = 5.9 Hz, 2H), 3.65 – 3.52 (m, 54H), 3.48 (t, *J* = 6.5 Hz, 2H), 3.37 – 3.34 (m, 4H), 2.50 (t, *J* = 5.9 Hz, 2H), 2.22 (t, *J* = 7.4 Hz, 4H), 1.90 (p, *J* = 7.5 Hz, 2H), 1.76 (p, *J* = 6.9 Hz, 2H), 1.61 – 1.56 (m, 2H), 1.49 – 1.44 (m, 2H), 1.42 – 1.37 (m, 2H).

^{13}C NMR (151 MHz, MeOD-d4) δ 175.4 (C), 174.1 (C), 161.1 (C), 154.8 (C), 142.5 (CH), 140.7 (C), 135.8 (C), 130.1 (CH), 128.7 (CH), 72.2 (CH_2), 71.6 (CH_2), 71.5 (CH_2), 71.5 (CH_2), 71.5 (CH_2), 71.4 (CH_2), 71.3 (CH_2), 71.2 (CH_2), 70.6 (CH_2), 70.1 (CH_2-O), 68.3 (CH_2), 45.7 (CH_2), 43.7 (CH_2-NH), 40.4 (CH_2), 40.4 (CH_2), 37.8 (CH_2), 36.1 (CH_2), 33.7 (CH_2), 30.5 (CH_2), 27.7 (CH_2), 26.5 (CH_2), 23.2 (CH_2). (three Cq are missing).

^{19}F NMR (564 MHz, MeOD-d4) δ -77.0.

HRMS (ESI): calc. for $C_{55}H_{95}ClN_8O_{18}$ $[M+2H]^+$: 595.3221, found: 595.3206.

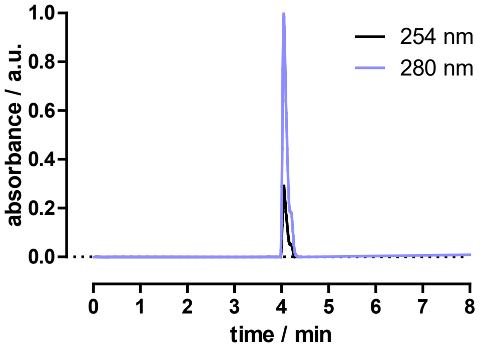

4-((4-(((2-Amino-9*H*-purin-6-yl)oxy)methyl)benzyl)carbamoyl)-2-(6-(azetidin-1-yl)-3-(3-carboxyazetidin-1-ium-1-ylidene)-3*H*-xanthen-9-yl)benzoate 3

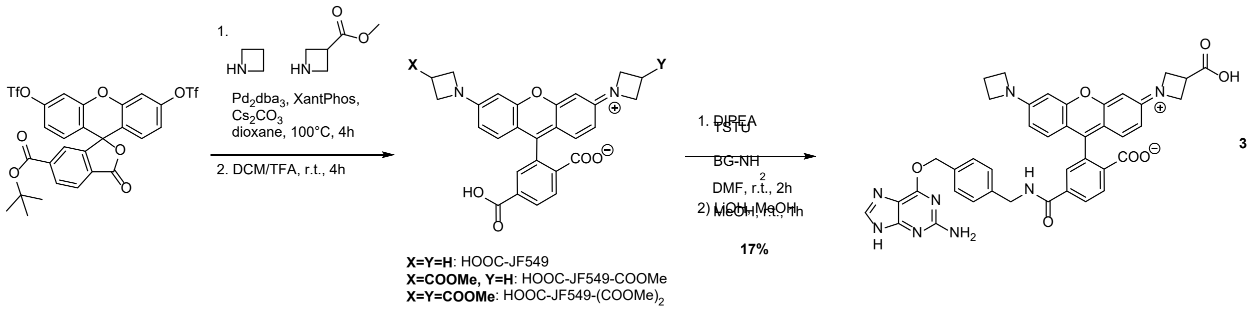

A Schenck tube was charged with 6-*tert*-butoxycarbonylfluorescein ditriflate (50.0 mg, 71.8 µmol), Pd_2dba_3 (6.6 mg, 7.18 µmol, 0.1 equiv.), XantPhos (12.5 mg, 21.5 µmol, 0.3 equiv.), Cs_2CO_3 (117 mg, 359 µmol 5 equiv.), methyl azetidine-3-carboxylate hydrochloride (16.3 mg, 108 µmol, 1.5 equiv.) and were dissolved in dioxane (1.0 mL). under N_2. Azetidine (6 µL, 89.0 µmol, 1.25 equiv.) was added in one drop. The Schlenck tube was sealed and evacuated/backfilled with N_2 (3×). The reaction mixture was stirred at 100°C during 4h. After cooling to r.t., the crude mixture was filtrated through a plug of Celite and washed with MeOH than concentrated under vacuum. The crude residue was then diluted in DCM/TFA (5.00 mL, 3:1) and stirred during 4h. The crude material was concentrated to dryness. The residue obtained was dissolved in a DMSO:H_2O:MeCN:AcOH (10:25:25:1) and subjected to RP-HPLC (MeCN:H_2O+0.1% TFA = 10:90 to 90:10 over 60 minutes), to afford an inseparable mixture of HOOC-JF549, HOOC-JF549-COOMe and HOOC-JF549-$(COOMe)_2$ as a red powder.

In a round bottom flask a mixture of HOOC-JF549, HOOC-JF549-COOMe and HOOC-JF549-$(COOMe)_2$ (4.0 mg, 7.79 µmol, 1.0 equiv.) was dissolved in 500 µL DMF, before DIPEA (11.0 µL, 62.3 µmol, 8.0 equiv.) and a 100 mM solution of TSTU (117 µL, 11.7 µmol, 1.5 equiv.) in DMF were added successively. The reaction mixture was allowed to incubate for 30 minutes, then BG-NH_2 (2.5 mg, 9.35 µmol, 1.2 equiv.) was added. The reaction mixture was incubated at r.t. until complete conversion, then 1 ml of a 1 M LiOH solution in THF/MeOH (2:1) was added. The reaction mixture was stirred at r.t. until complete conversion was observed (by LCMS). The mixture was quenched with 150 µL HOAc, diluted with H_2O:MeCN:AcOH (25:25:1) and subjected to RP-HPLC (MeCN:H_2O+0.1% TFA = 10:90 to 90:10 over 60 minutes) to obtain 1.0 mg (17%) of **3** after lyophilization as a red powder. The side products were isolated and put aside for other projects of the group.

^{1}**H NMR** (600 MHz, MeOD-d4) δ 8.42 – 8.37 (m, 1H), 8.23 – 8.18 (m, 1H), 7.96 (d, J = 1.6 Hz, 1H), 7.82 (d, J = 1.8 Hz, 1H), 7.50 (d, J = 8.0 Hz, 2H), 7.40 (d, J = 8.0 Hz, 2H), 7.08 (dd, J = 9.3, 7.4 Hz, 2H), 6.66 – 6.60 (m, 3H), 6.56 (dd, J = 4.2, 2.2 Hz, 1H), 5.60 (s, 2H), 4.59 (s, 2H), 4.49 – 4.45 (m, 2H), 4.41 – 4.37 (m, 2H), 4.35 – 4.30 (m, 4H), 3.76 – 3.69 (m, 1H), 2.57 (p, J = 7.6 Hz, 2H).

^{19}**F NMR** (564 MHz, MeOD-d4) δ -77.1.

HRMS (ESI): calc. for $C_{41}H_{34}N_8O_7$ $[M+H]^+$: 751.2623, found: 751.2598.

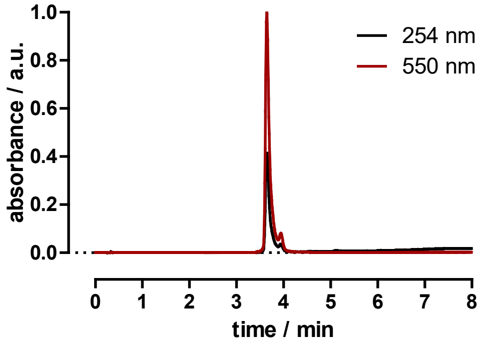

2-(6-(Azetidin-1-yl)-3-(3-carboxyazetidin-1-ium-1-ylidene)-3*H*-xanthen-9-yl)-4-((2-(2-((6-chlorohexyl)oxy)ethoxy)ethyl)-carbamoyl)benzoate 4

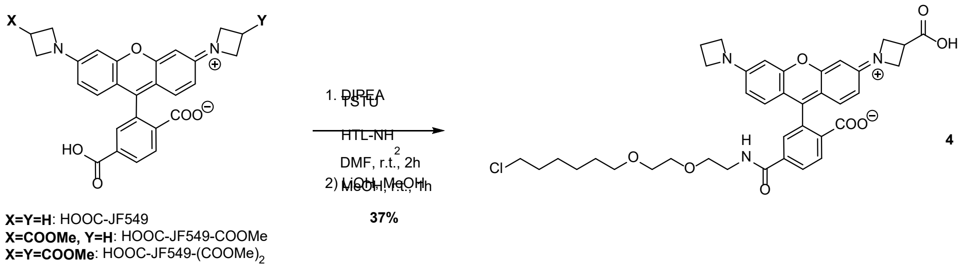

A 4 mL vial was charged with HTL-NHBoc (1.3 mg, 5.86 µmol, 1.2 equiv.) and 30 µL neat Trifluoroacetic acid (TFA) was added. The reaction mixture was stirred to ensure homogeneity and allowed to incubate for 30 min. All volatiles were removed under a gentle stream of nitrogen. The residue, HTL-NH_2, was taken up in 50 µL DMF. In round bottom flask, a mixture of HOOC-JF549, HOOC-JF549-COOMe and HOOC-JF549-$(COOMe)_2$ (2.0 mg, 3.89 µmol, 1.0 equiv.) was dissolved in DMF (400µL). DIPEA (5.4 µL, 31.2 µmol, 8.0 equiv.) and a 100 mM solution of TSTU (58.4 µL, 5.84 µmol, 1.5 equiv.) in DMF were added successively. The reaction mixture was allowed to incubate for 30 minutes, and then the HTL-NH_2 solution was added in one drop. The reaction mixture was incubated at r.t. until complete conversion, and then 1 ml of a 1 M LiOH solution in THF/MeOH (2:1) was added. The reaction mixture was stirred at r.t. until complete conversion was observed (by LCMS). The mixture was quenched with 100 µL HOAc, diluted with H_2O:MeCN:AcOH (25:25:1) and subjected to RP-HPLC (MeCN:H_2O+0.1% TFA = 10:90 to 90:10 over 60 minutes) to obtain 1.0 mg (37%) of **4** after lyophilization as a red powder.

^{1}H NMR (600 MHz, MeOD-d4) δ 8.40 (d, J = 8.2 Hz, 1H), 8.20 (dd, J = 8.2, 1.8 Hz, 1H), 7.81 (d, J = 1.8 Hz, 1H), 7.10 (dd, J = 9.1, 2.2 Hz, 2H), 6.67 – 6.62 (m, 3H), 6.57 (d, J = 2.1 Hz, 1H), 4.48 (t, J = 9.4 Hz, 2H), 4.42 – 4.37 (m, 2H), 4.33 (t, J = 7.7 Hz, 4H), 3.73 (tt, J = 9.1, 5.7 Hz, 1H), 3.66 (t, J = 5.4 Hz, 2H), 3.63 – 3.61 (m, 2H), 3.60 – 3.58 (m, 2H), 3.53 (t, J = 6.6 Hz, 2H), 3.43 (t, J = 6.5 Hz, 2H), 2.57 (p, J = 7.7 Hz, 2H), 1.72 (dt, J = 14.7, 6.8 Hz, 2H), 1.50 (dt, J = 14.6, 6.6 Hz, 2H), 1.41 (dq, J = 9.1, 7.0 Hz, 2H), 1.35 – 1.30 (m, 4H). . In accordance with previous characterizations.[1]

^{19}F NMR (564 MHz, MeOD-d4) δ -77.0.

HRMS (ESI): calc. for $C_{38}H_{42}ClN_3O_8$ $[M+H]^+$: 704.2734, found: 704.2707.

[1] Deo *et al. J. Am. Chem. Soc.* **2019**, *141*, 13734−13738.

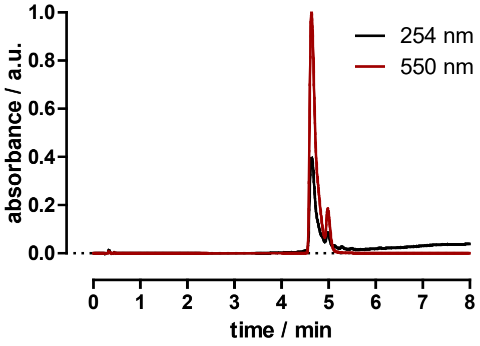
absorbance / a.u.
1.0
0.8
0.6
0.4
0.2
0.0
0
1
2
3
4
5
6
7
8
time / min
254 nm
550 nm

2-(3-(3-(2-aminoethyl)azetidin-1-ium-1-ylidene)-7-(azetidin-1-yl)-5,5-dimethyl-3,5-dihydrodibenzo[b,e]silin-10-yl)-4-carboxybenzoate 5

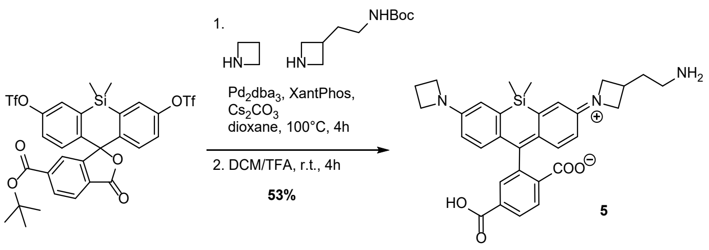

5

A Schenck tube was charged with 6-*tert*-butoxycarbonylsilafluorescein ditriflate (50.0 mg, 67.7 µmol), Pd_2dba_3 (6.2 mg, 6.77 µmol, 0.1 equiv.), XantPhos (11.7 mg, 20.3 µmol, 0.3 equiv.), Cs_2CO_3 (110 mg, 338 µmol 5 equiv.), tert-butyl (2-(azetidin-3-yl)ethyl)carbamate hydrochloride (24.0 mg, 102 µmol, 1.5 equiv.) were dissolved in dioxane (1.0 mL). with N_2. Azetidine (5.5 µL, 81.2 µmol, 1.2 equiv.) was added in one drop. The Schlenck tube was sealed and evacuated/backfilled with N_2 (3×). The reaction mixture was stirred at 100°C during 4h. After cooling to r.t., the crude mixture was filtrated through a plug of Celite and washed with MeOH than concentrated under vacuum. The crude residue was then diluted in DCM/TFA (5.00 mL, 3:1) and stirred during 4h. The crude material was concentrated to dryness. The residue obtained was dissolved in a DMSO:H_2O:MeCN:AcOH (10:25:25:1) and subjected to RP-HPLC (MeCN:H_2O+0.1% TFA = 10:90 to 90:10 over 60 minutes), to afford 19.2 mg (53 %) of **5** as a blue powder.

^{1}H NMR (600 MHz, DMSO) δ 8.12 (d, J = 7.9 Hz, 1H), 8.05 (d, J = 7.9 Hz, 1H), 7.64 (s, 1H), 6.75 – 6.67 (m, 4H), 6.38 – 6.31 (m, 2H), 3.95 (td, J = 7.5, 4.9 Hz, 3H), 3.83 (t, J = 7.3 Hz, 4H), 3.52 – 3.47 (m, 2H), 2.82 – 2.76 (m, 2H), 2.76 – 2.70 (m, 1H), 2.29 (q, J = 7.3 Hz, 2H), 1.88 (q, J = 7.7 Hz, 2H), 0.61 (s, 3H), 0.51 (s, 3H).
^{13}C NMR (151 MHz, DMSO) δ 169.2 (C), 166.0, 158.1, 150.8, 136.4, 131.3 (C), 131.1 (C), 130.0 (CH), 129.0 (C), 128.6 (C), 128.0 (C), 127.1 (2 × CH), 126.0 (CH), 124.2 (CH), 115.5 (2 × CH), 112.9 (2 × CH), 56.6 (2 × CH_2-N), 51.9 (2 × CH_2-N), 36.9 (CH_2), 31.6 (CH_2), 26.9 (CH), 16.3 (CH_2), -0.2 (CH_3), -1.0 (CH_3).
^{19}F NMR (564 MHz, DMSO) δ -77.0.
HRMS (ESI): calc. for $C_{31}H_{34}N_3O_4Si$ $[M+H]^+$: 540.2314, found: 540.2344.

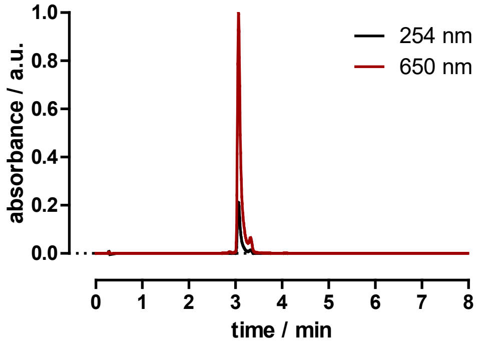

2-(3-(3-(1-(9*H*-Fluoren-9-yl)-3,16-dioxo-2,7,10,13-tetraoxa-4,17-diazanonadecan-19-yl) azetidin-1-ium-1-ylidene)-7-(azetidin-1-yl)-5,5-dimethyl-3,5-dihydrodibenzo[*b,e*]silin-10-yl)-4-carboxybenzoate 6

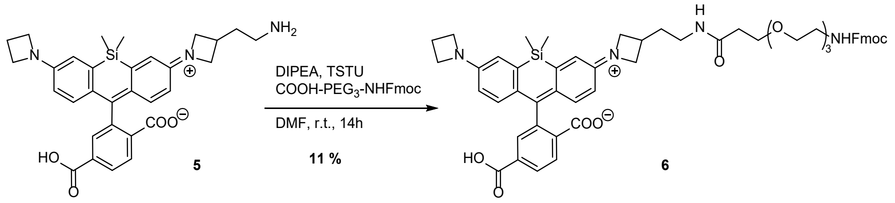

In an Eppendorf tube, HOOC-PEG$_3$-Fmoc (12.3 mg, 11.1 µmol,1.2 equiv.) was dissolved in DMF (500 µL), before DIPEA (25.8 µL, 148 µmol, 8.0 equiv.) and TSTU (12.6 mg, 41.7 µmol, 2.25 equiv.) were added. The mixture was stirred for 15 min at r.t., before a solution of HOOC-JF646-NH$_2$ (10 mg, 9.27 µmol, 1.0 equiv.) and DIPEA (12.9 µL, 74.1 µmol, 4.0 equiv.) in 200 µL DMF was added. The reaction mixture was allowed to incubate overnight. The reaction mixture was then quenched with 50 µL HOAc, diluted with H_2O:MeCN:AcOH (25:25:1) and subjected to RP-HPLC (MeCN:H_2O+0.1% TFA = 10:90 to 90:10 over 60 minutes) to obtain 2.0 mg (11%) of **6** after lyophilization as a blue powder.

^{1}H NMR (600 MHz, MeOD-d4) δ 8.28 (s, 2H), 7.82 (s, 1H), 7.76 (d, *J* = 7.6 Hz, 2H), 7.61 (d, *J* = 7.8 Hz, 2H), 7.36 (t, *J* = 7.5 Hz, 2H), 7.27 (t, *J* = 7.5 Hz, 2H), 6.90 – 6.79 (m, 4H), 6.27 (ddd, *J* = 13.9, 9.3, 2.6 Hz, 2H), 4.39 – 4.19 (m, 8H), 4.14 (t, *J* = 6.9 Hz, 1H), 3.91 – 3.80 (m, 2H), 3.71 (t, *J* = 6.0 Hz, 2H), 3.62 – 3.52 (m, 8H), 3.47 (t, *J* = 5.6 Hz, 2H), 3.26 – 3.18 (m, 5H), 2.88 – 2.81 (m, 1H), 2.49 (p, *J* = 7.6 Hz, 2H), 2.41 (t, *J* = 5.9 Hz, 2H), 1.84 (q, *J* = 7.0 Hz, 2H), 0.58 (s, 3H), 0.51 (s, 3H).

^{19}F NMR (564 MHz, MeOD-d4) δ -77.4.

HRMS (ESI): calc. for $C_{55}H_{62}N_4O_{10}Si$ [M+2H]$^{2+}$: 483.2112, found: 483.2088.

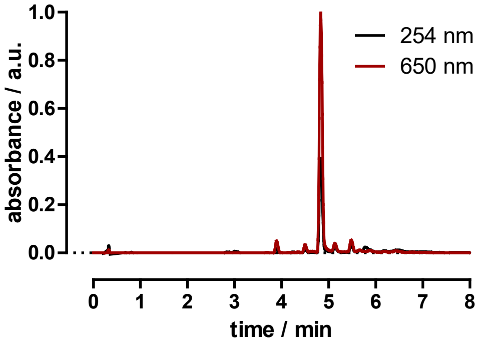

2-(3-(3-(1-Amino-12-oxo-3,6,9-trioxa-13-azapentadecan-15-yl)azetidin-1-ium-1-ylidene)-7-(azetidin-1-yl)-5,5-dimethyl-3,5-dihydrodibenzo[b,e]silin-10-yl)-4-((4-(((2-amino-9*H*-purin-6-yl)oxy)methyl)benzyl)carbamoyl)benzoate 7

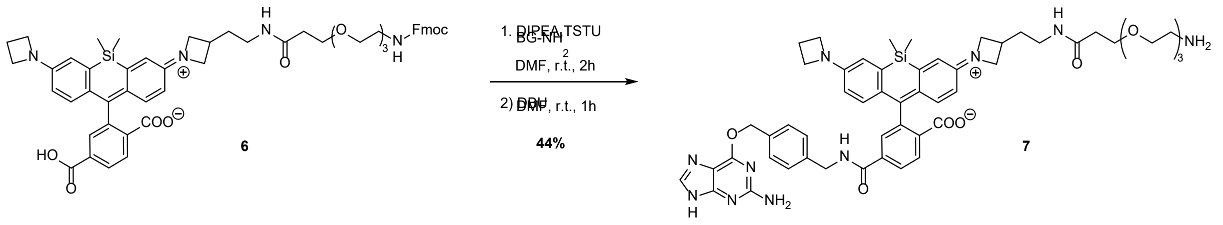

In an Eppendorf, **6** (0.55 mg, 0.57 µmol, 1.0 equiv.) was dissolved in 250 µL DMF, before DIPEA (0.8 µL, 4.6 µmol, 8.0 equiv.) and a 100 mM solution of TSTU (8.5 µL, 0.85 µmol, 1.5 equiv.) in DMF were added successively. The reaction was stirred 15 minutes, then BG-NH_2 (0.2 mg, 0.68 µmol, 1.2 equiv.) was added and the reaction mixture was allowed to incubate at r.t. until complete conversion. 2.5 µL DBU was added and the reaction mixture was stirred for one additional hour, before it was quenched with 30 µL HOAc, diluted with H_2O:MeCN:AcOH (25:25:1) and subjected to RP-HPLC (MeCN:H_2O+0.1% TFA = 10:90 to 90:10 over 60 minutes) to obtain 0.25 µmol (44%) of the desired product after lyophilization as a blue powder.

^{1}H NMR (600 MHz, MeOD-d4) δ 8.08 – 8.03 (m, 2H), 7.99 (s, 1H), 7.67 (s, 1H), 7.49 (d, J = 7.9 Hz, 2H), 7.36 (d, J = 7.9 Hz, 2H), 6.81 – 6.71 (m, 4H), 6.37 – 6.32 (m, 2H), 5.56 (s, 2H), 4.55 (s, 2H), 4.13 – 4.04 (m, 3H), 4.03 – 3.92 (m, 5H), 3.73 (t, J = 6.0 Hz, 2H), 3.65 – 3.54 (m, 10H), 3.23 (t, J = 6.8 Hz, 2H), 3.07 (t, J = 5.2 Hz, 2H), 2.82 – 2.77 (m, 1H), 2.44 (t, J = 6.0 Hz, 2H), 2.42 – 2.38 (m, 2H), 1.85 (q, J = 7.1 Hz, 2H), 0.59 (s, 3H), 0.53 (s, 3H).

^{19}F NMR (564 MHz, DMSO) δ -77.0.

HRMS (ESI): calc. for $C_{53}H_{64}N_{10}O_8Si$ $[M+2H]^+$: 498.2334, found: 498.2276.

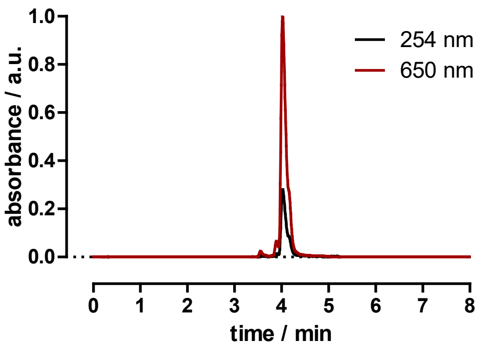

2-(3-(3-(1-Amino-12-oxo-3,6,9-trioxa-13-azapentadecan-15-yl)azetidin-1-ium-1-ylidene)-7-(azetidin-1-yl)-5,5-dimethyl-3,5-dihydrodibenzo[b,e]silin-10-yl)-4-((2-(2-((6-chlorohexyl)oxy)ethoxy)ethyl)carbamoyl)benzoate 8

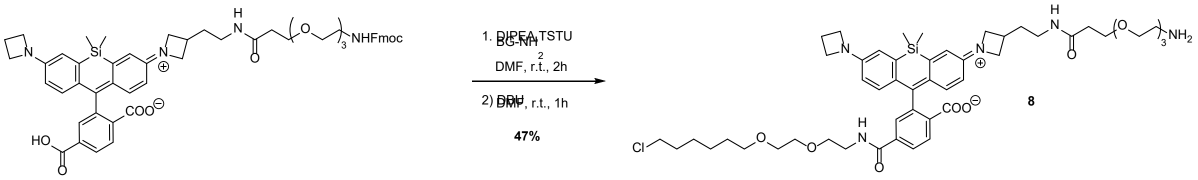

A 4 mL vial was charged with HTL-NHBoc (1.0 mg, 3.09 µmol) and 30 µL neat Trifluoroacetic acid (TFA) was added. The reaction mixture was stirred to ensure homogeneity and allowed to incubate for 30 min. All volatiles were removed under a gentle stream of nitrogen. The residue, HTL-NH_2, was taken up in 50 µL DMF. In an Eppendorf, **6** (0.55 mg, 0.57 µmol, 1.0 equiv.) was dissolved in 250 µL DMF, before DIPEA (0.8 µL, 4.6 µmol, 8.0 equiv.) and a 100 mM solution of TSTU (8.5 µL, 0.85 µmol, 1.5 equiv.) in DMF were added successively. The reaction was stirred 15 minutes, then CA-NH_2 (11 µL, 0.68 µmol, 1.2 equiv.) was added and the reaction mixture was allowed to incubate at r.t. until complete conversion. 2.5 µL DBU was added and the reaction mixture was stirred for one additional hour, before it was quenched with 30 µL HOAc, diluted with H_2O:MeCN:AcOH (25:25:1) and subjected to RP-HPLC (MeCN:H_2O+0.1% TFA = 10:90 to 90:10 over 60 minutes) to obtain 0.27 µmol (47%) of the desired product after lyophilization as a blue powder.

^{1}H NMR (600 MHz, MeOD-d4) δ 8.03 (t, J = 6.6 Hz, 2H), 7.67 (s, 2H), 6.75 (d, J = 2.6 Hz, 2H), 6.72 (dd, J = 8.7, 5.7 Hz, 2H), 6.35 (dt, J = 8.8, 3.4 Hz, 2H), 4.03 (s, 3H), 3.94 – 3.87 (m, 4H), 3.73 (t, J = 6.0 Hz, 2H), 3.66 – 3.58 (m, 14H), 3.57 – 3.52 (m, 6H), 3.49 (t, J = 6.7 Hz, 2H), 3.39 (t, J = 6.5 Hz, 2H), 3.23 (t, J = 6.8 Hz, 2H), 3.08 (t, J = 5.1 Hz, 2H), 2.80 – 2.75 (m, 2H), 2.44 (t, J = 6.0 Hz, 2H), 2.38 (q, J = 7.3 Hz, 2H), 1.84 (q, J = 7.1 Hz, 2H), 1.71 – 1.64 (m, 2H), 1.49 – 1.42 (m, 2H), 1.39 – 1.33 (m, 2H), 0.62 (s, 3H), 0.53 (s, 3H).

HRMS (ESI): calc. for $C_{50}H_{72}ClN_5O_9Si$ $[M+2H]^+$: 474.7389, found: 474.7425.

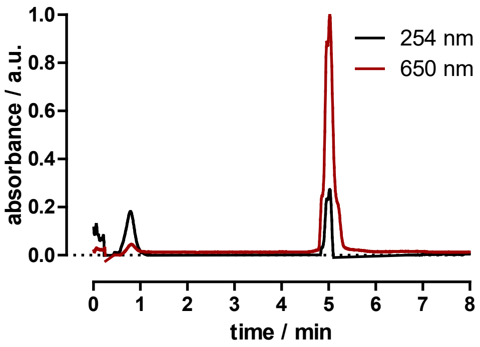

4-((4-(((2-Amino-9*H*-purin-6-yl)oxy)methyl)benzyl)carbamoyl)-2-(6-(azetidin-1-yl)-3-(3-((15-(1-(7-(azetidin-1-yl)-10-(2-carboxylato-5-((2-(2-((6-chlorohexyl)oxy)ethoxy)ethyl) carbamoyl)phenyl)-5,5-dimethyldibenzo[b,e]silin-3(5*H*)-ylidene)azetidin-1-ium-3-yl)-12-oxo-3,6,9-trioxa-13-azapentadecyl)carbamoyl)azetidin-1-ium-1-ylidene)-3*H*-xanthen-9-yl)benzoate (MT36)

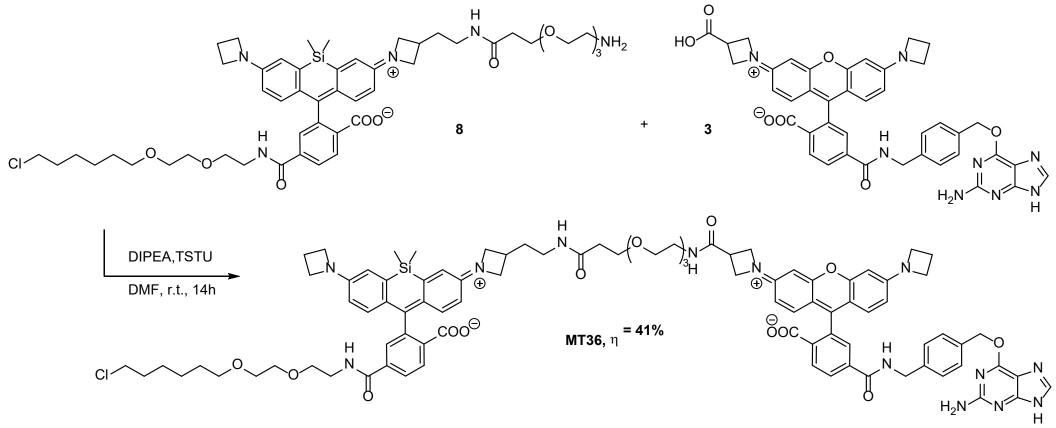

An Eppendorf tube was charged with a solution of **3** (25 µL, 250 nmol, 1.0 equiv.) in DMF, before DIPEA (0.5 µL, 2.0 µmol, 8.0 equiv.) and a 100 mM solution of TSTU (3.8 µL, 0.375 µmol, 1.5 equiv.) in DMF were added successively and diluted with DMF (100 µL in total). The reaction mixture was stirred at r.t. during 30 minutes. A solution of **8** (250 nmol, 1.0 equiv.) in DMF (100 µL) was added. The reaction mixture was stirred until complete conversion, before it was quenched with 50 µL HOAc, diluted with H_2O:MeCN:AcOH (25:25:1) and subjected to RP-HPLC (MeCN:H_2O+0.1% TFA = 10:90 to 90:10 over 60 minutes) to obtain 102 nmol of the desired product after lyophilization in 41% yield as a purple powder.

^{1}H NMR (600 MHz, MeOD-d4) δ 8.38 (dt, J = 6.2, 2.0 Hz, 1H), 8.21 (d, J = 8.1 Hz, 1H), 8.03 – 7.91 (m, 3H), 7.86 – 7.80 (m, 1H), 7.63 (d, J = 7.9 Hz, 1H), 7.48 – 7.45 (m, 2H), 7.40 – 7.35 (m, 2H), 7.08 – 7.01 (m, 2H), 6.75 (s, 2H), 6.73 – 6.70 (m, 1H), 6.59 – 6.43 (m, 4H), 6.36 – 6.32 (m, 1H), 6.31 – 6.24 (m, 2H), 5.52 (d, J = 2.9 Hz, 2H), 4.59 (s, 2H), 4.38 – 4.23 (m, 7H), 4.05 – 3.99 (m, 1H), 3.97 – 3.91 (m, 3H), 3.74 – 3.71 (m, 1H), 3.64 – 3.46 (m, 21H), 3.16 (t, J = 6.9 Hz, 2H), 2.79 – 2.72 (m, 2H), 2.56 – 2.50 (m, 2H), 2.46 – 2.40 (m, 2H), 2.40 – 2.33 (m, 2H), 2.00 – 1.94 (m, 2H), 1.83 – 1.78 (m, 2H), 1.68 – 1.65 (m, 2H), 1.53 – 1.43 (m, 4H), 0.60 (d, J = 5.5 Hz, 3H), 0.53 (s, 2H), 0.49 (s, 1H). (One CH aliphatic and two CH_2 are missing).
^{19}F NMR (564 MHz, DMSO) δ -77.0.
HRMS (ESI): calc. for $C_{91}H_{105}ClN_{13}O_{15}Si$ $[M+3H]^+$: 560.9098, found: 560.9113.

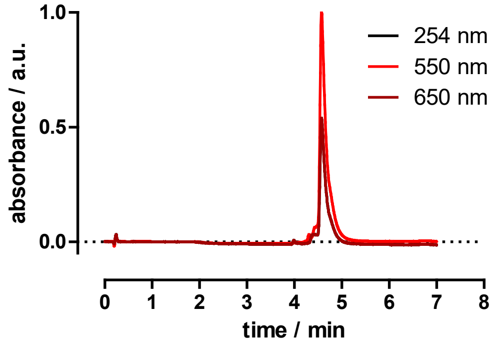

1.0
0.5
0.0
absorbance / a.u.
254 nm
550 nm
650 nm
0
1
2
3
4
5
6
7
8
time / min

4-((4-(((2-Amino-9*H*-purin-6-yl)oxy)methyl)benzyl)carbamoyl)-2-(7-(azetidin-1-yl)-3-(3-(1-(1-(6-(azetidin-1-yl)-9-(2-carboxylato-5-((2-(2-((6-chlorohexyl)oxy)ethoxy)ethyl) carbamoyl)phenyl)-3*H*-xanthen-3-ylidene)azetidin-1-ium-3-yl)-1,14-dioxo-5,8,11-trioxa-2,15-diazaheptadecan-17-yl)azetidin-1-ium-1-ylidene)-5,5-dimethyl-3,5-dihydrodibenzo[b,e]silin-10-yl)benzoate (MT37)

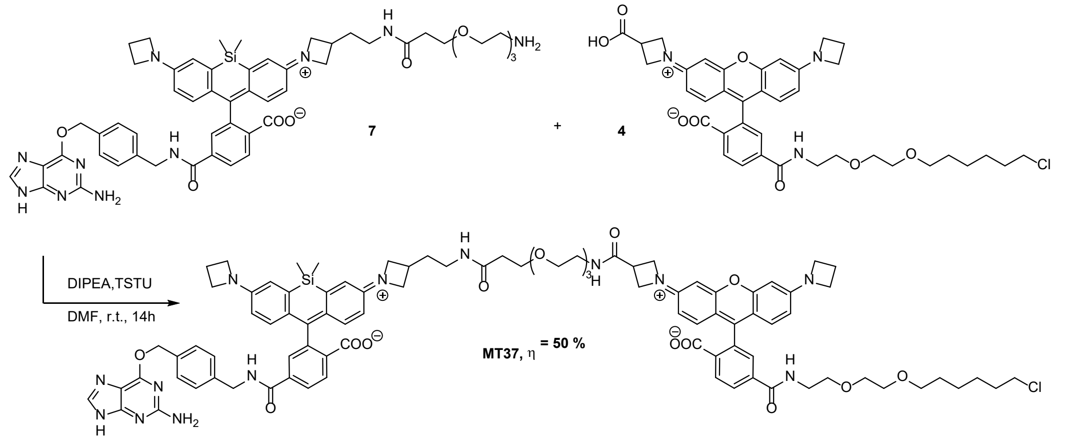

An Eppendorf tube was charged with a solution of **4** (25 µL, 250 nmol, 1.0 equiv.) in DMF, before DIPEA (0.5 µL, 2.0 µmol, 8.0 equiv.) and a 100 mM solution of TSTU (3.8 µL, 0.375 µmol, 1.5 equiv.) in DMF were added successively and diluted with DMF (100 µL in total). The reaction mixture was stirred at r.t. during 30 minutes. A solution of **7** (250 nmol, 1.0 equiv.) in DMF (100 µL) was added. The reaction mixture was stirred until complete conversion, before it was quenched with 50 µL HOAc, diluted with H_2O:MeCN:AcOH (25:25:1) and subjected to RP-HPLC (MeCN:H_2O+0.1% TFA = 10:90 to 90:10 over 60 minutes) to obtain 125 nmol of the desired product after lyophilization in 50% yield as a purple powder.

^{1}H NMR (600 MHz, MeOD-d4) δ 8.37 (d, J = 8.1 Hz, 1H), 8.18 (d, J = 8.4 Hz, 1H), 8.10 – 7.96 (m, 3H), 7.88 – 7.79 (m, 1H), 7.66 (d, J = 14.9 Hz, 1H), 7.44 (d, J = 7.9 Hz, 2H), 7.33 (d, J = 5.5 Hz, 2H), 7.02 – 6.93 (m, 2H), 6.81 – 6.72 (m, 4H), 6.51 – 6.38 (m, 4H), 6.36 – 6.24 (m, 2H), 5.48 (s, 2H), 4.52 (s, 2H), 4.35 – 4.20 (m, 8H), 4.06 (s, 2H), 4.03 – 3.95 (m, 3H), 3.76 – 3.70 (m, 3H), 3.69 – 3.47 (m, 21H), 3.22 (s, 2H), 3.17 – 3.13 (m, 1H), 2.83 – 2.74 (m, 1H), 2.58 – 2.49 (m, 3H), 2.47 – 2.36 (m, 4H), 1.87 – 1.79 (m, 2H), 1.73 – 1.65 (m, 2H), 1.63 – 1.57 (m, 2H), 1.51 – 1.43 (m, 2H), 0.58 (d, J = 6.1 Hz, 3H), 0.51 (d, J = 11.7 Hz, 3H). (One CH_2 is missing).

^{19}F NMR (564 MHz, DMSO) δ -77.0.

HRMS (ESI): calc. for $C_{91}H_{105}ClN_{13}O_{15}Si$ $[M+3H]^{+}$: 560.9098, found: 560.9113.

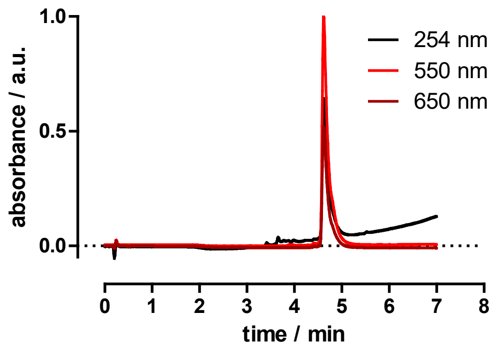
1.0
0.5
0.0
absorbance / a.u.
254 nm
550 nm
650 nm
0
1
2
3
4
5
6
7
8
time / min

NMR analysis

^{1}H NMR (600 MHz, $CDCl_3$) of 1

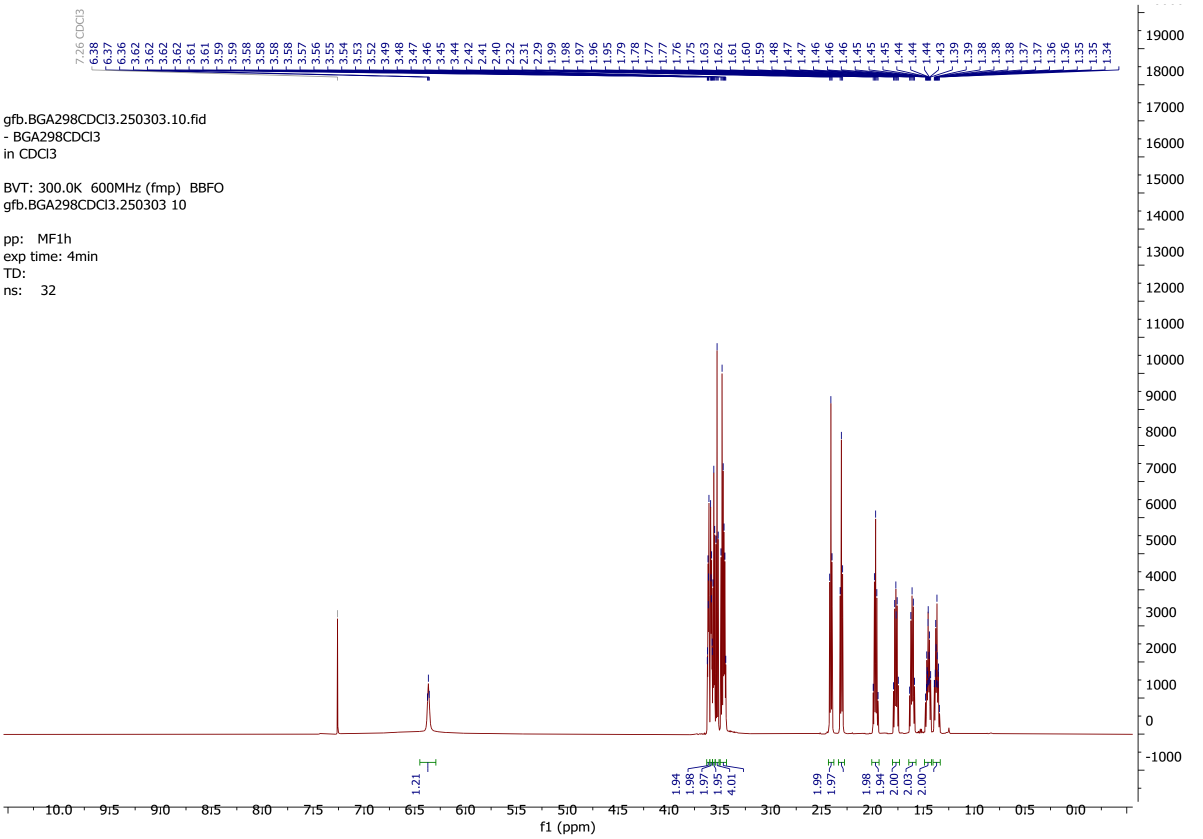

^{13}C NMR (151 MHz, $CDCl_3$) of 1

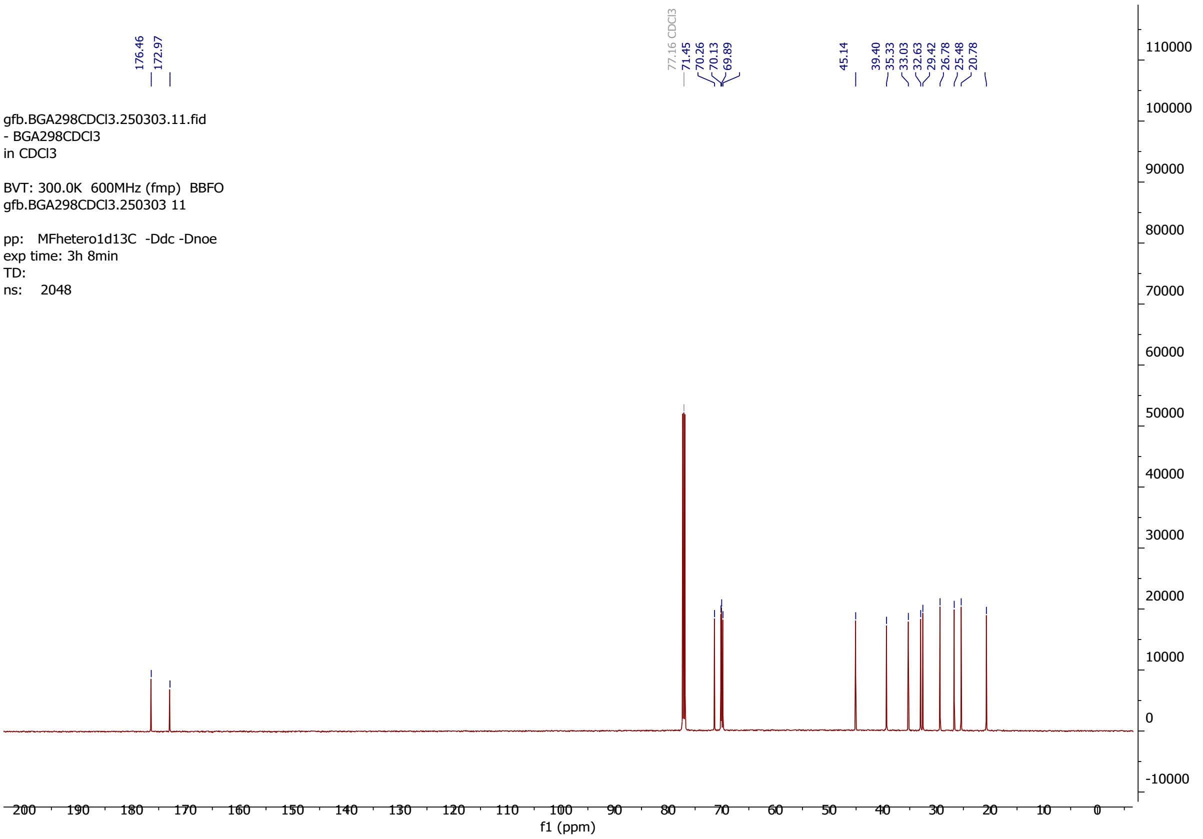

^{19}F NMR (564 MHz, $CDCl_3$) of **1**

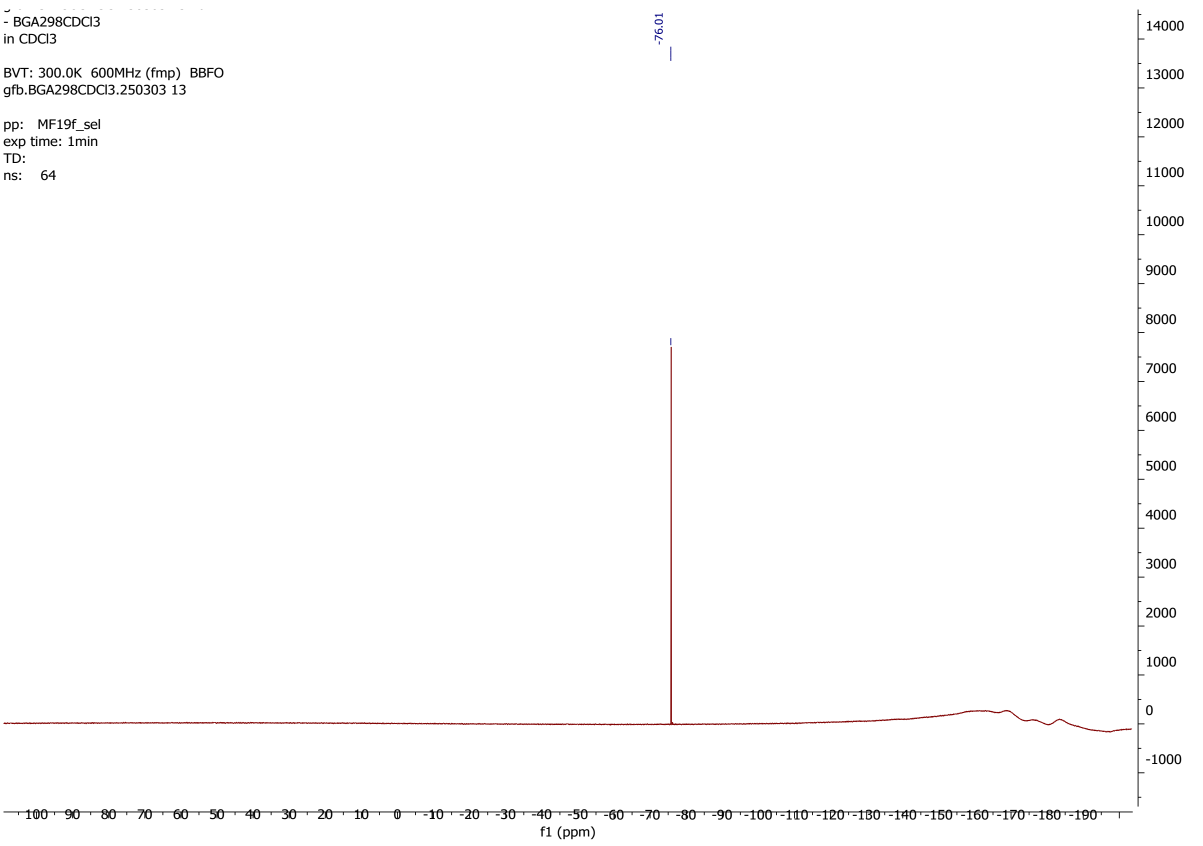

LC-MS of 1

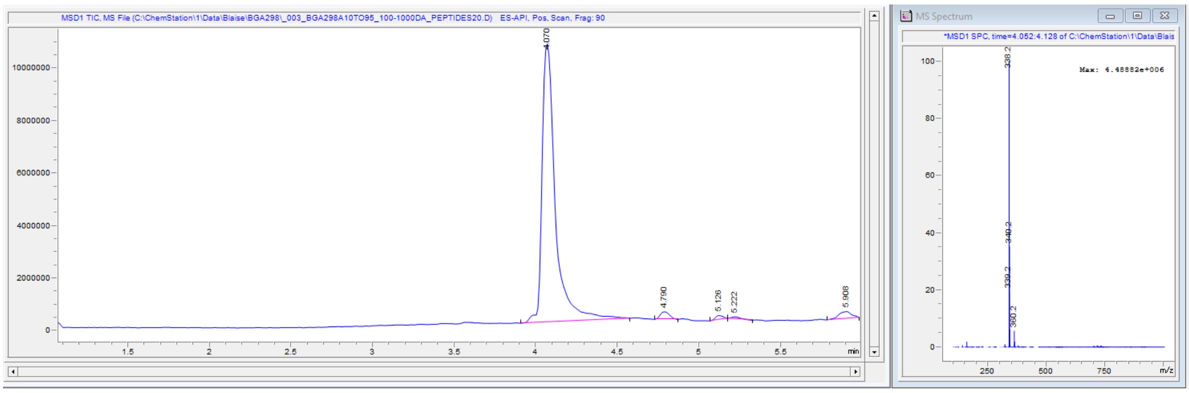

HRMS of 1

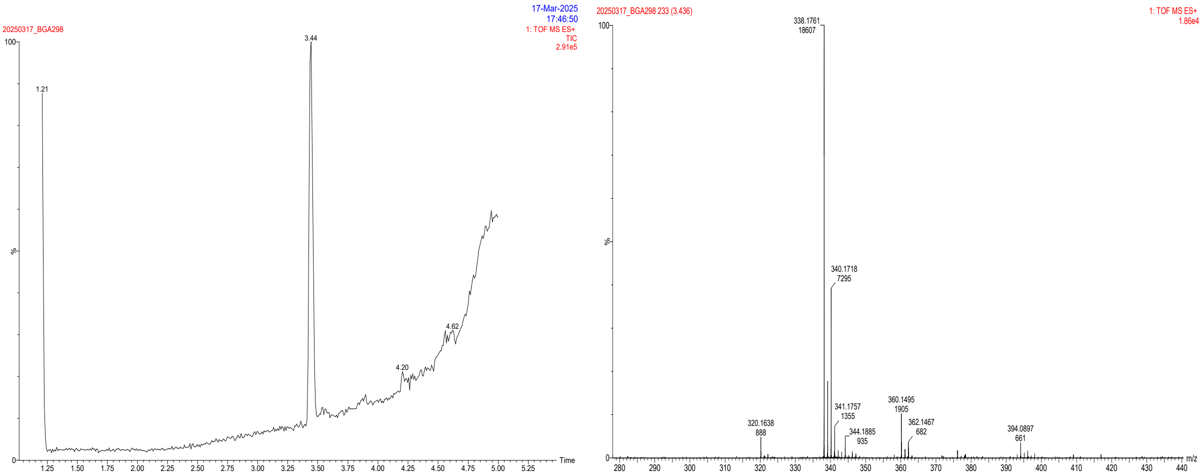

^{1}H NMR (600 MHz, MeOD-d4) of **2a**

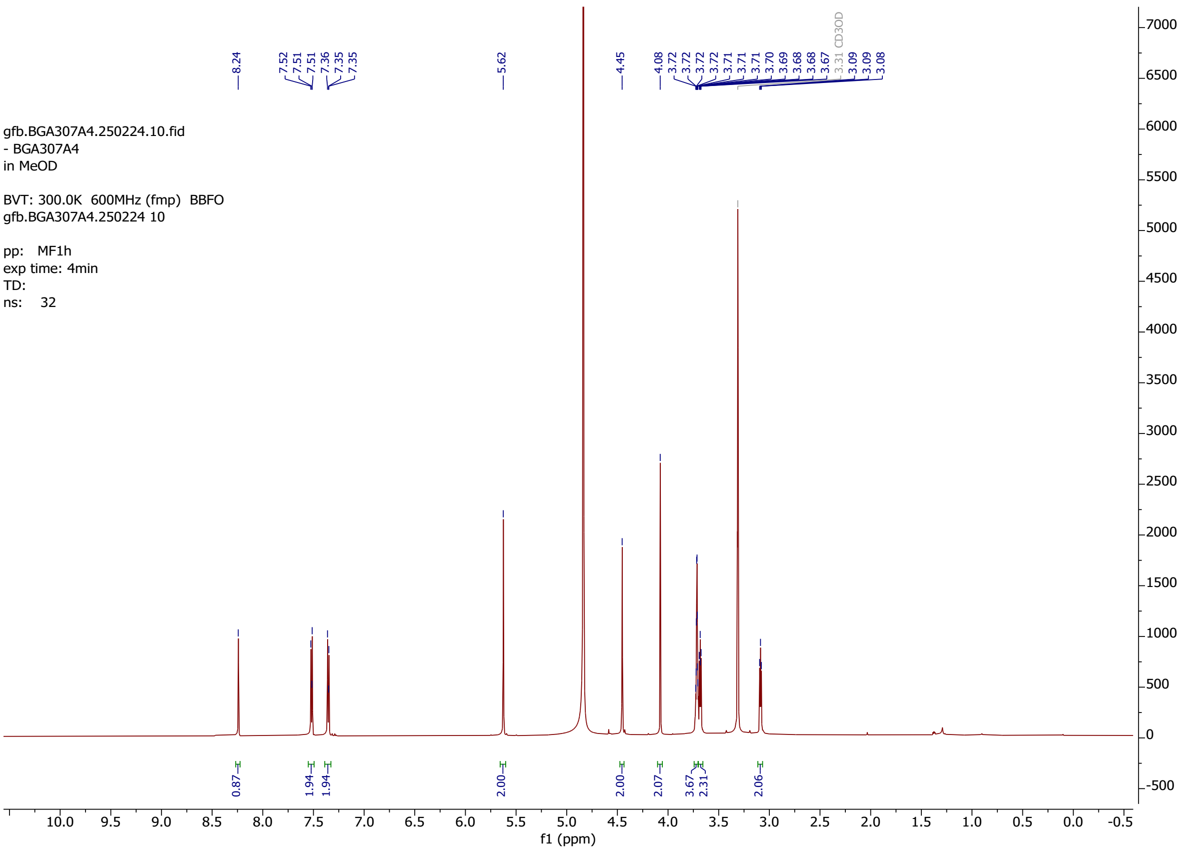

^{13}C NMR (151 MHz, MeOD-d4) of **2a**

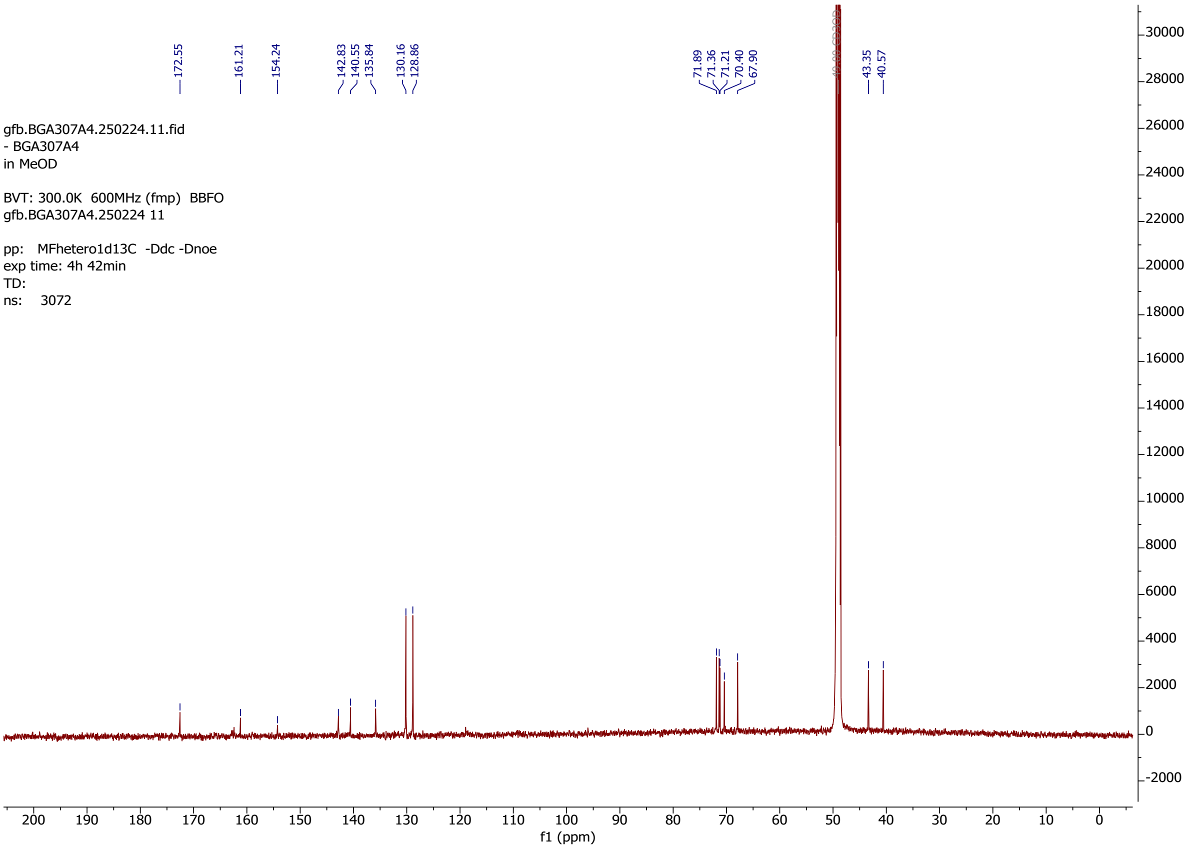

^{19}F NMR (564 MHz, $CDCl_3$) of **2a**

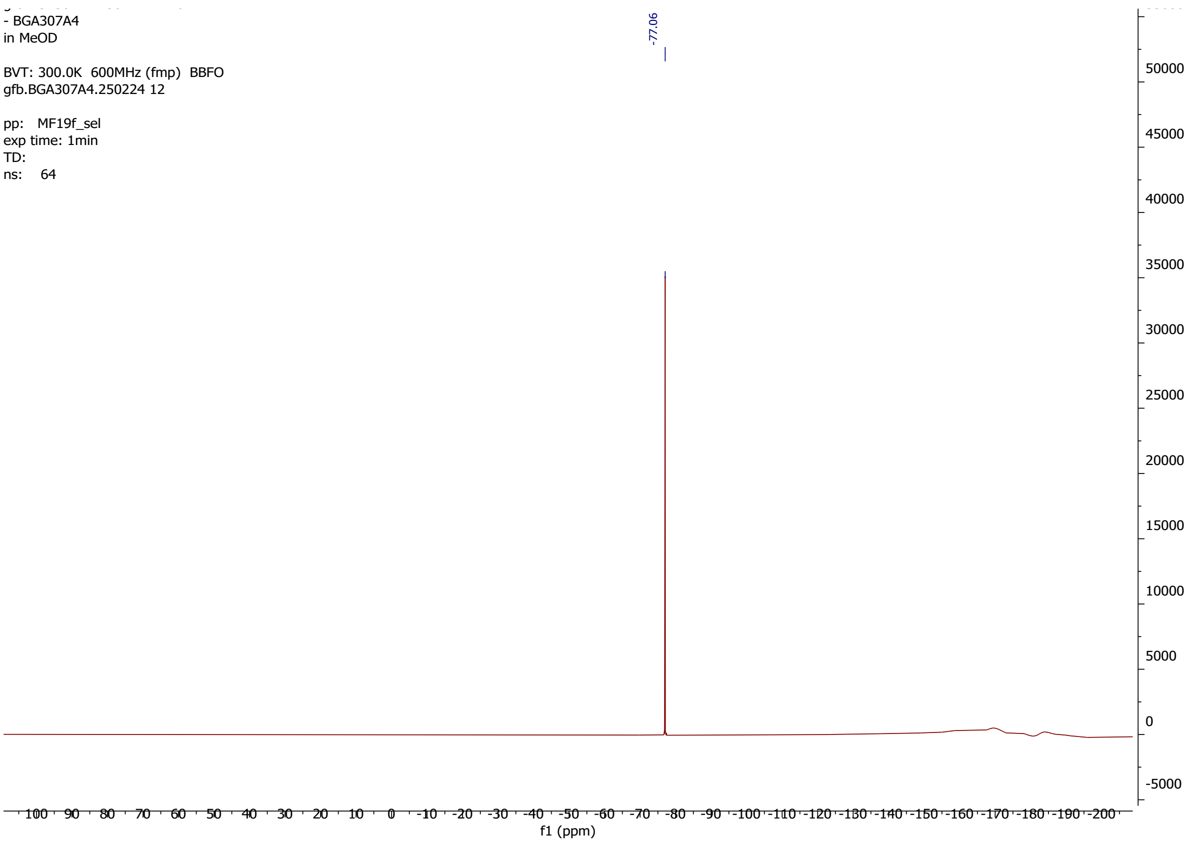

LC-MS of **2a**

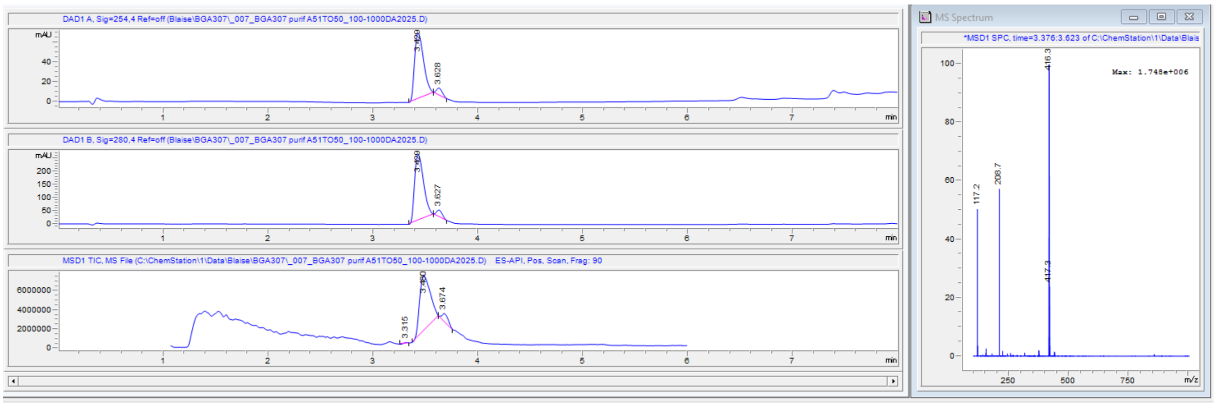

HRMS of **2a**

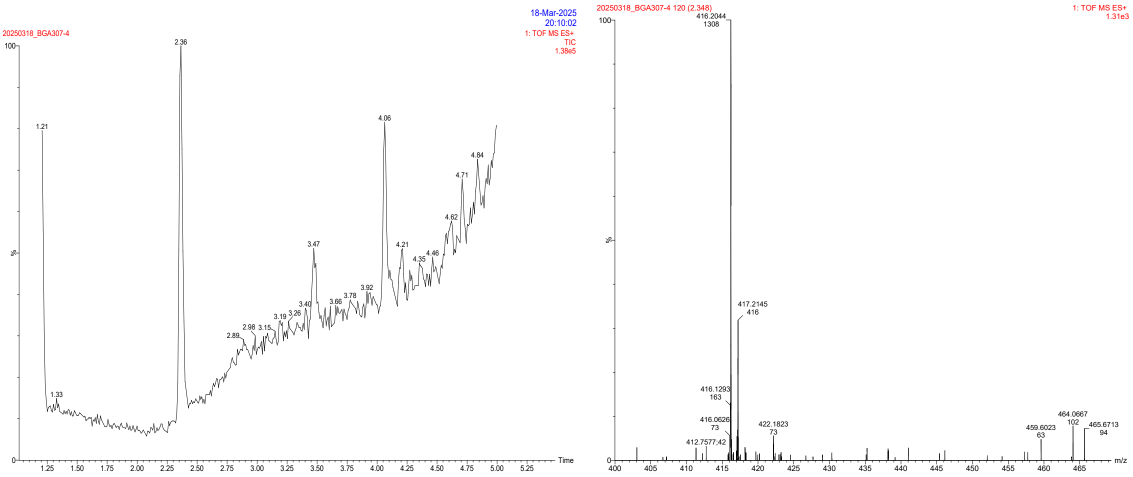

^{1}H NMR (600 MHz, MeOD-d4) of **2b**

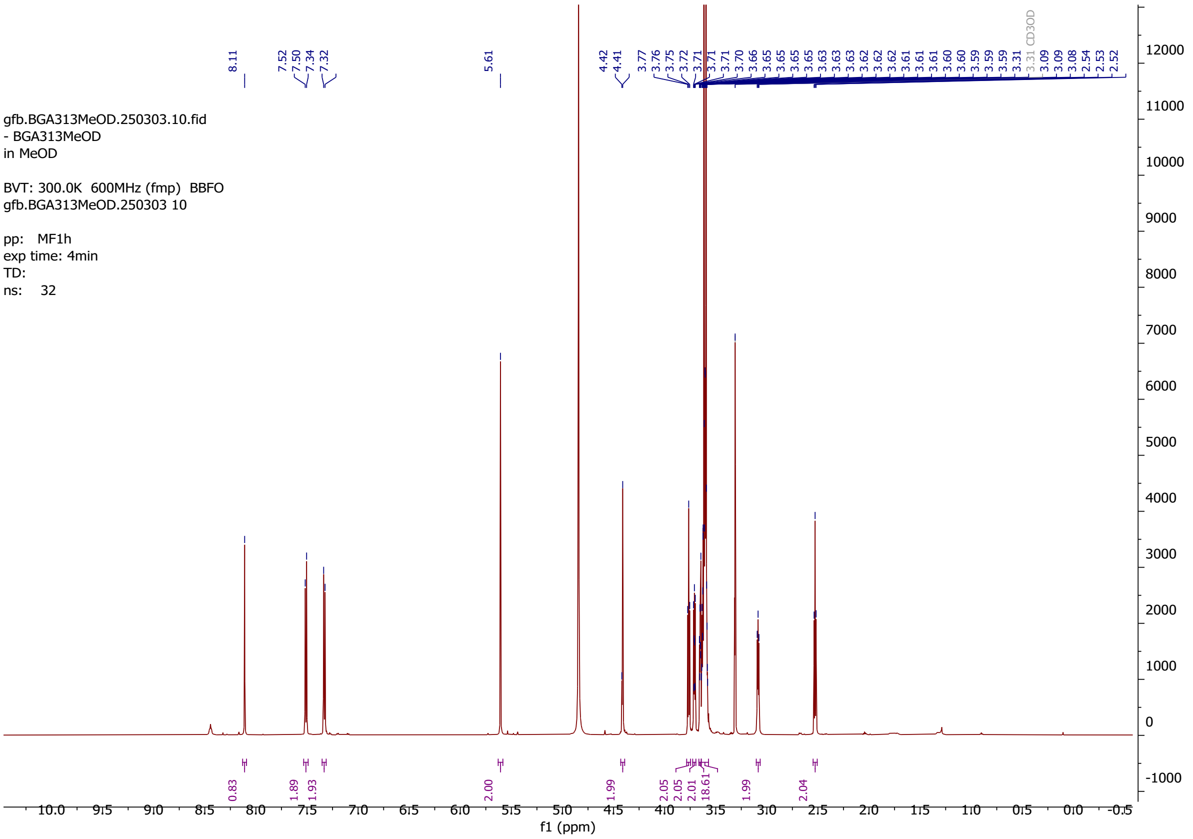

^{13}C NMR (151 MHz, MeOD-d4) of **2b**

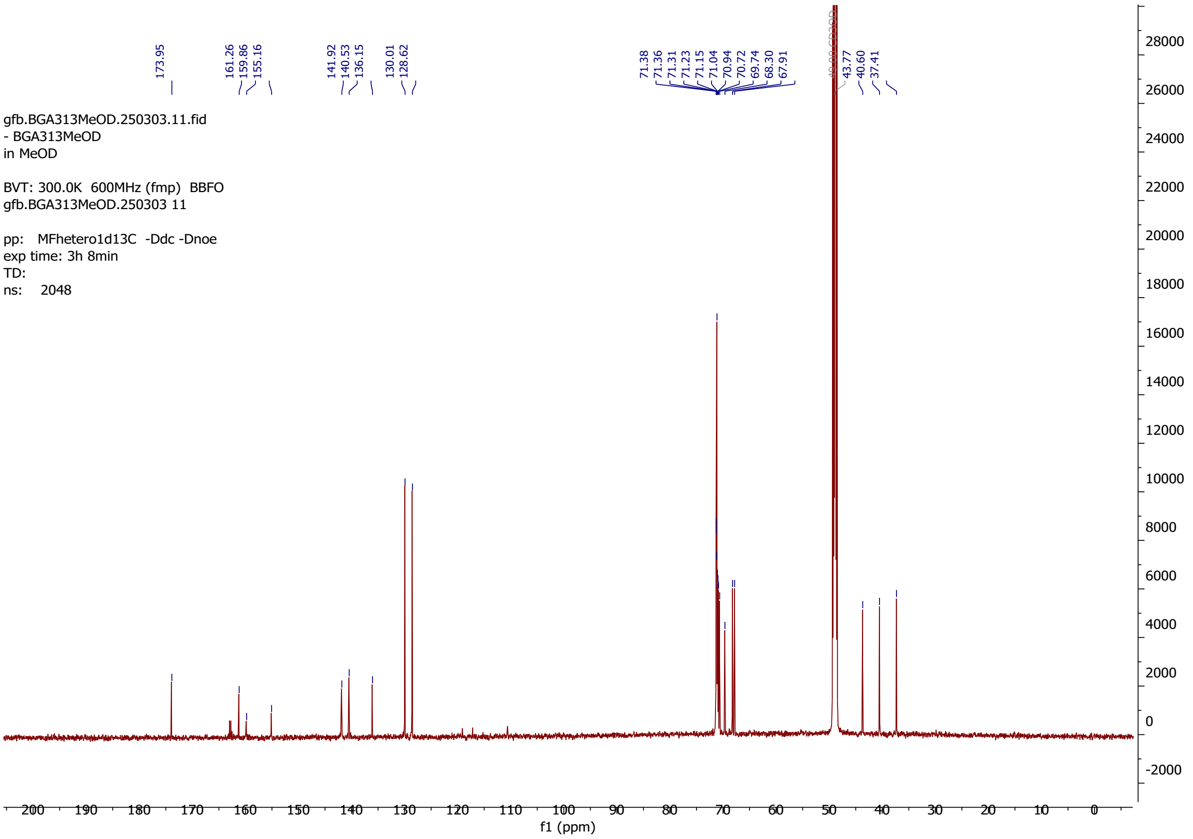

LC-MS of **2b**

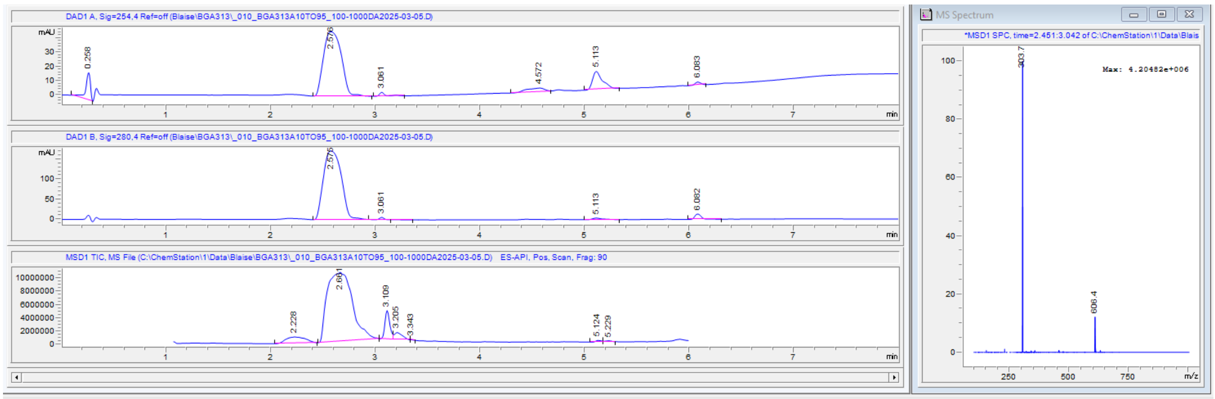

HRMS of **2b**

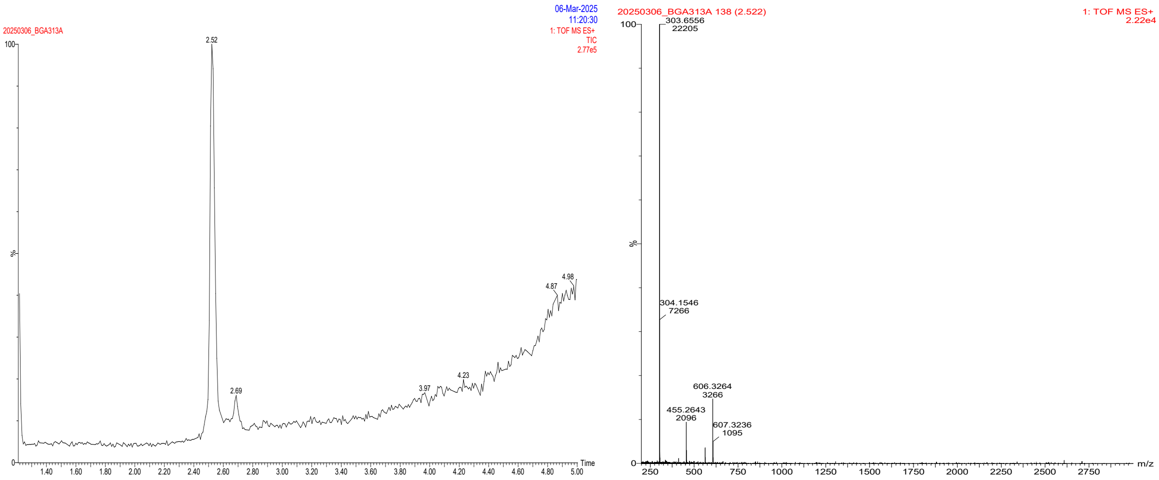

¹⁹F NMR (564MHz, MeOD-d4) of **2b**

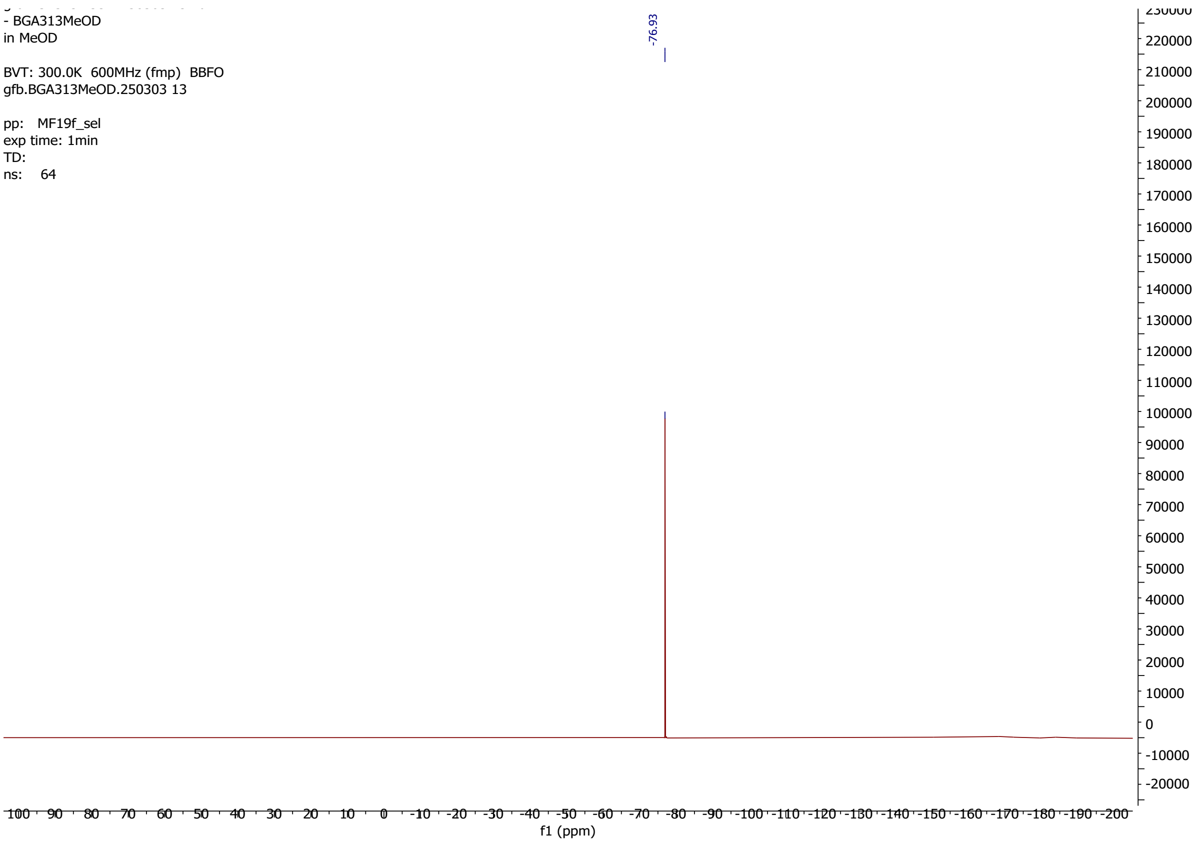

^{1}H NMR (600 MHz, MeOD-d4) of **2c**

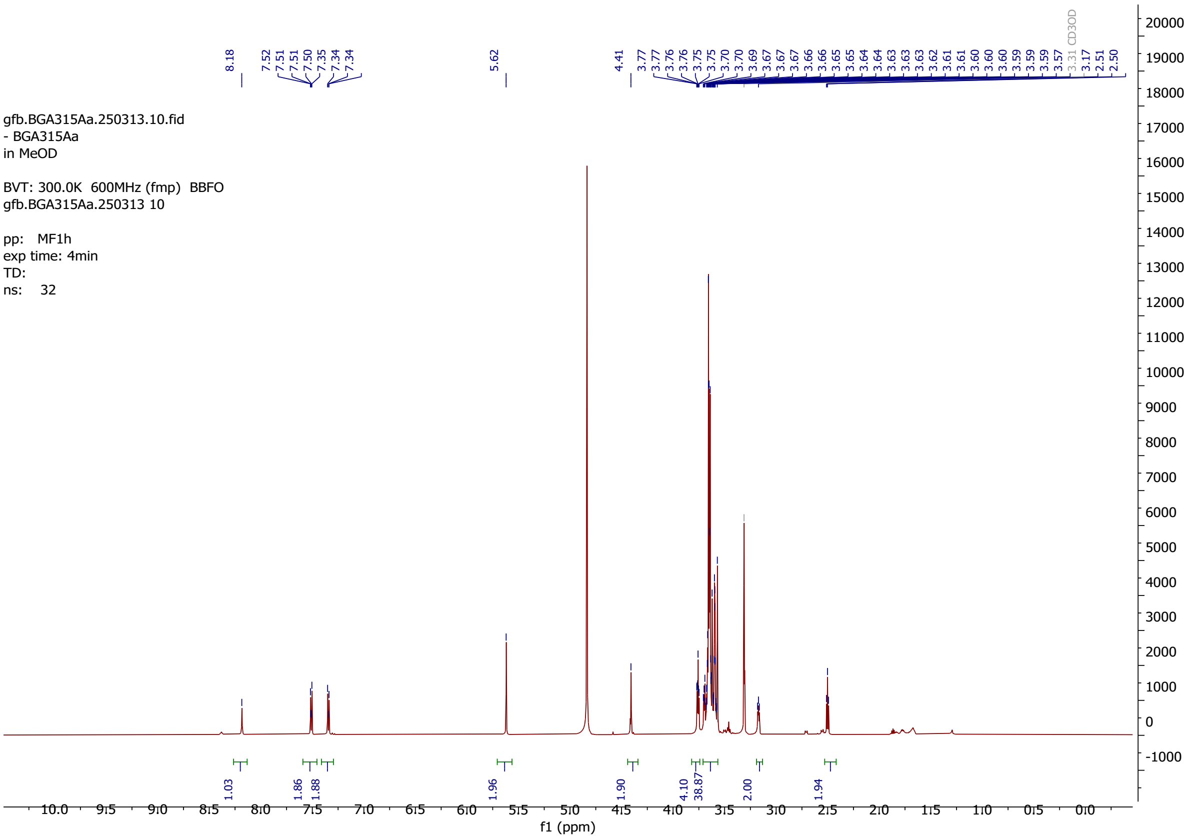

^{13}C NMR (151 MHz, MeOD-d4) of **2c**

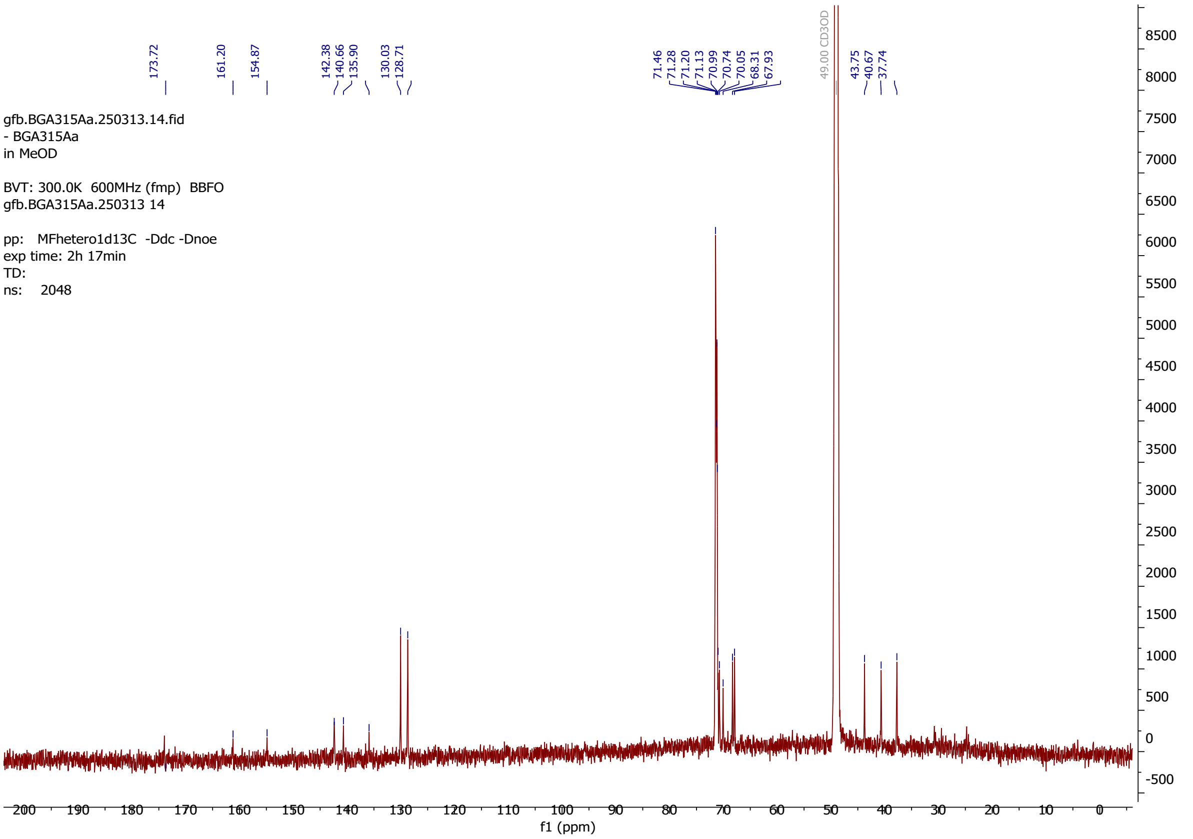

^{19}F NMR (564MHz, MeOD-d4) of **2c**

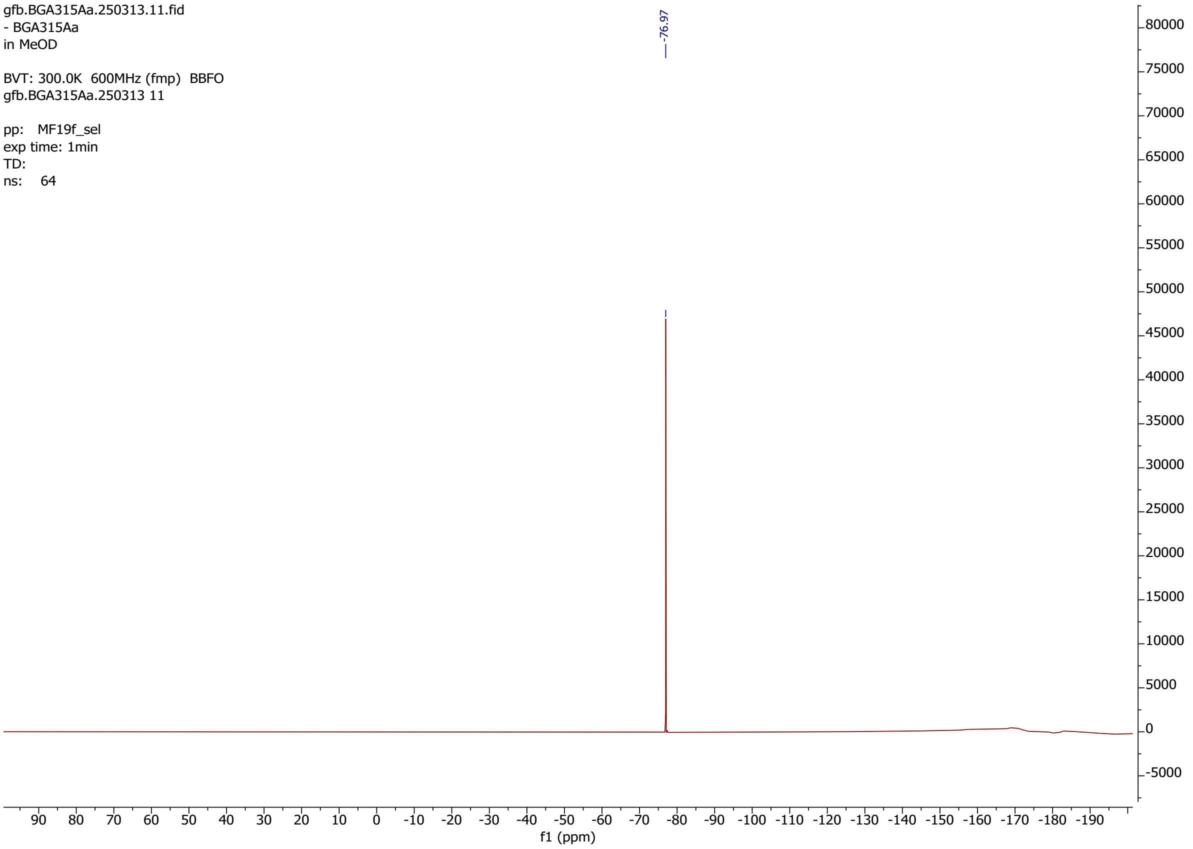

LC-MS of 2c

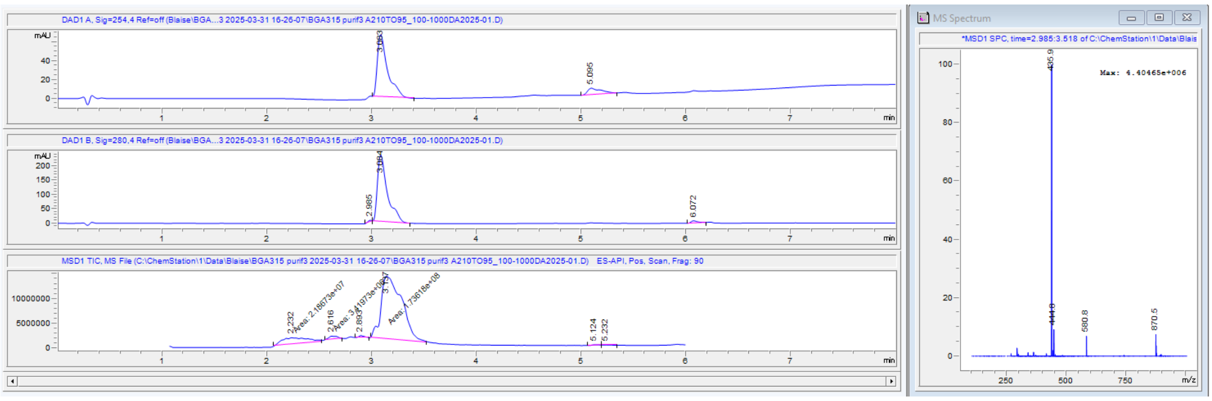

HRMS of 2c

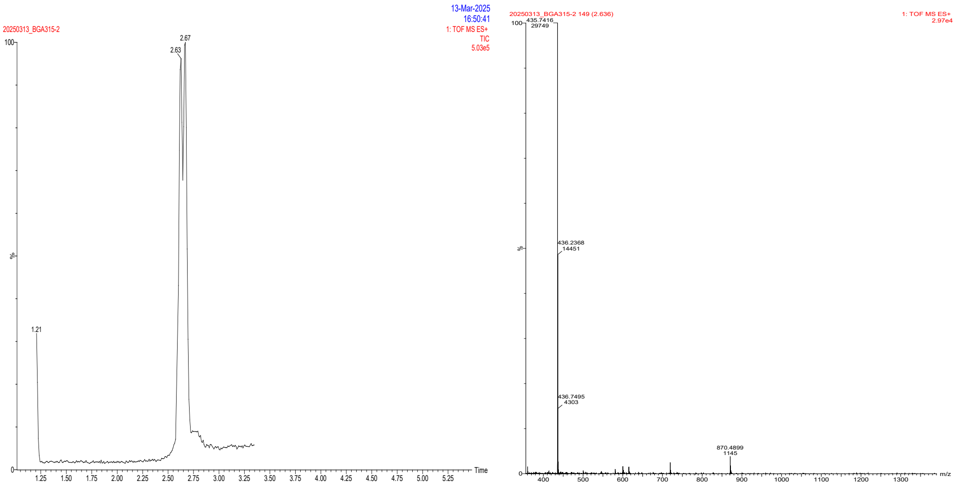

^{1}H NMR (600 MHz, MeOD-d4) of **BG-PEG2-HTL**

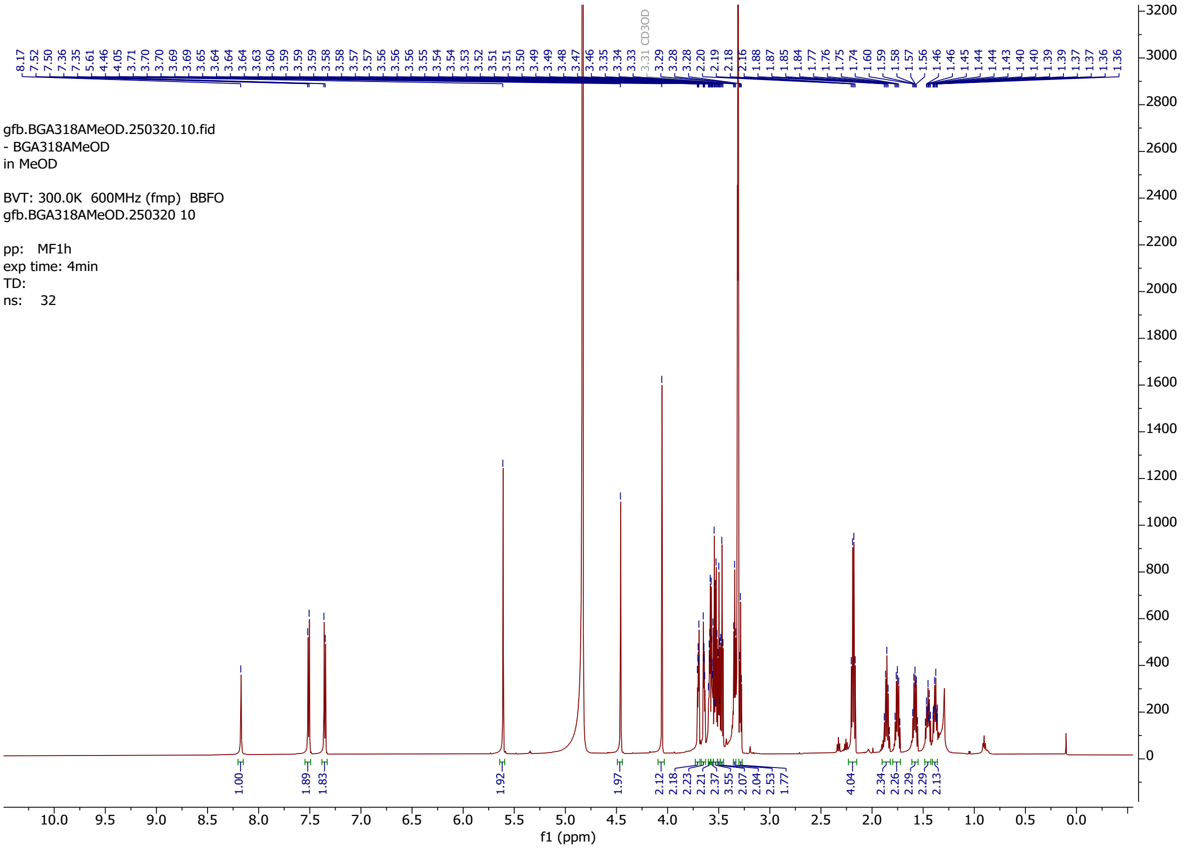

^{13}C NMR (151 MHz, MeOD-d4) of **BG-PEG2-HTL**

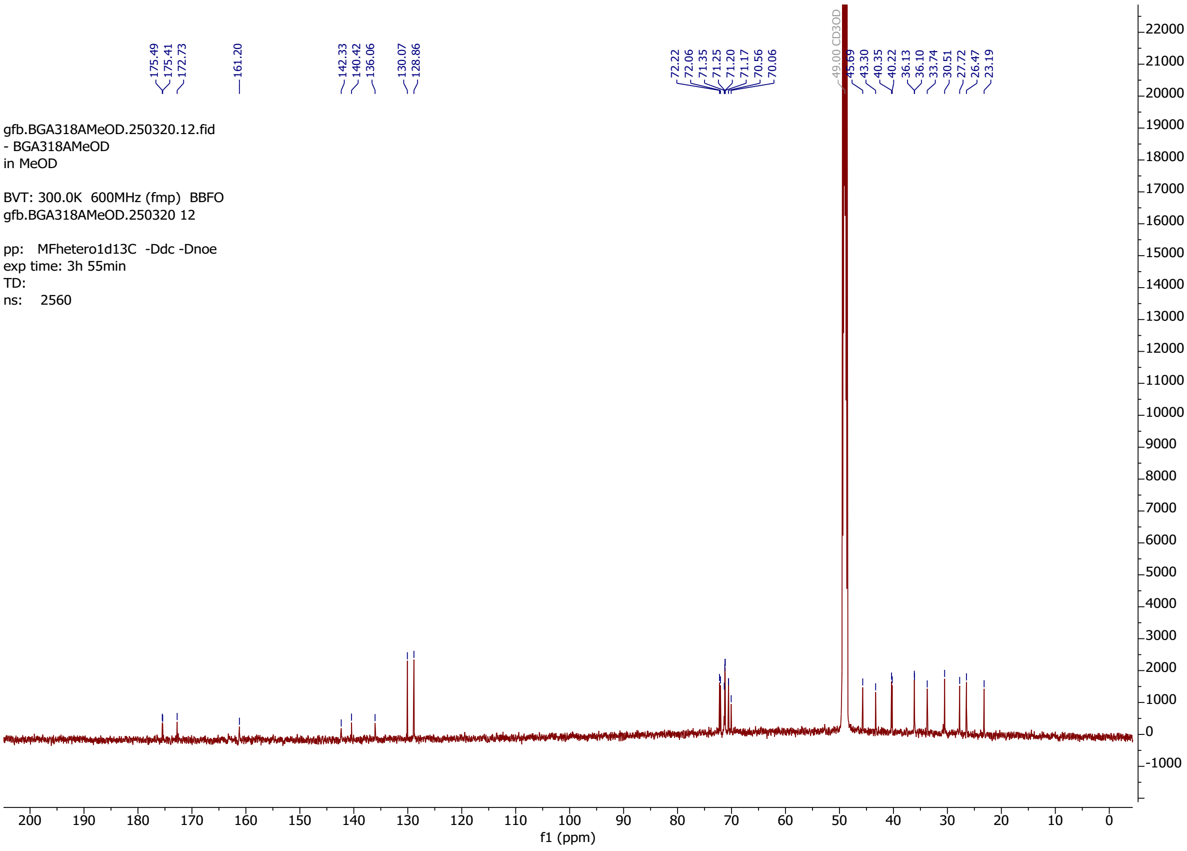

^{19}F NMR (564MHz, MeOD-d4) of **BG-PEG2-HTL**

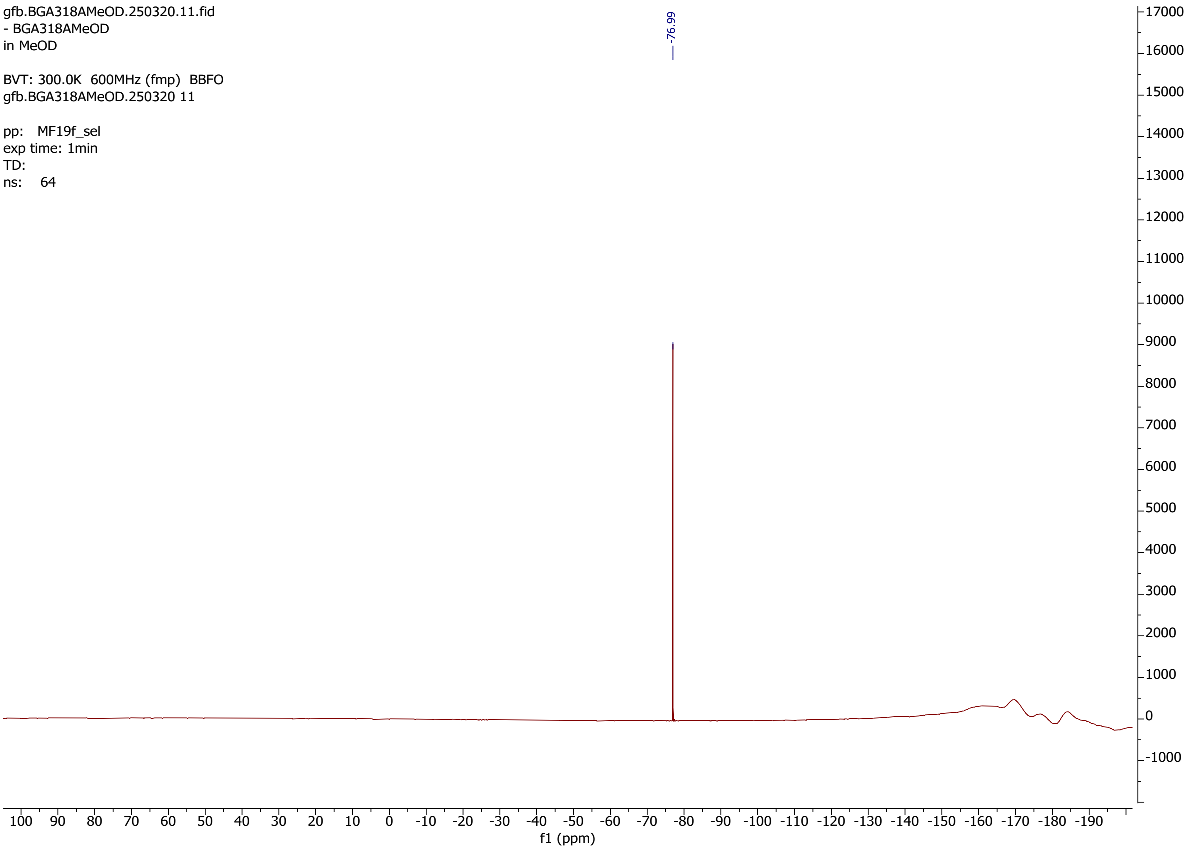

LC-MS of BG-PEG2-HTL

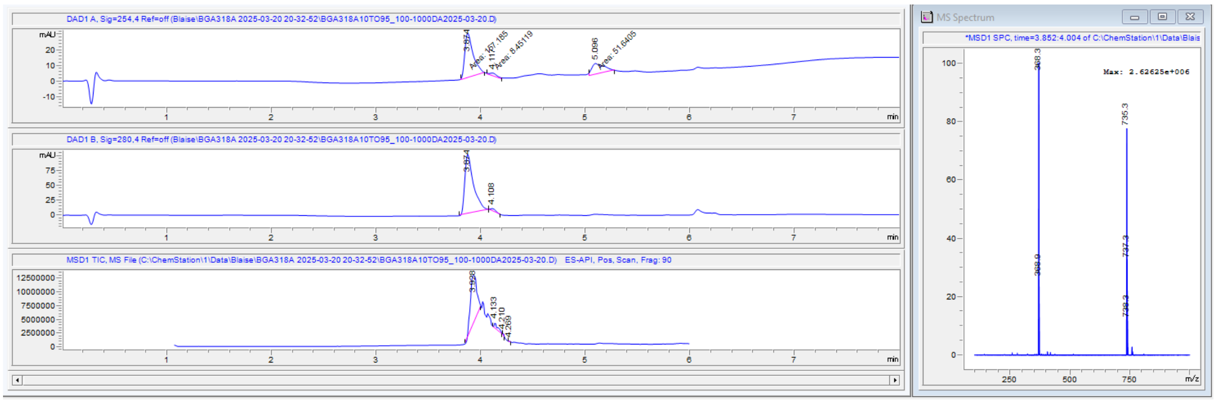

HRMS of BG-PEG2-HTL

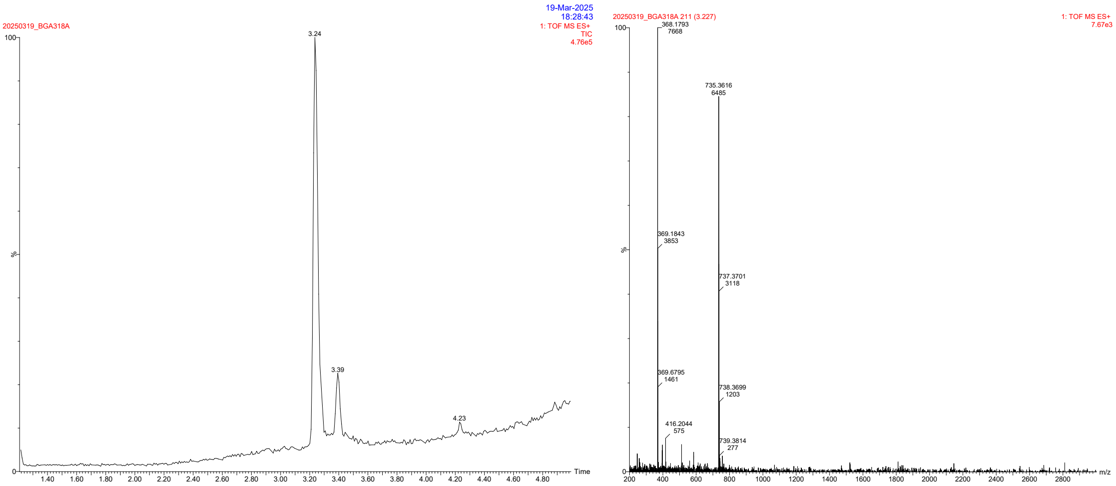

^{1}H NMR (600 MHz, MeOD-d4) of **BG-PEG6-HTL**

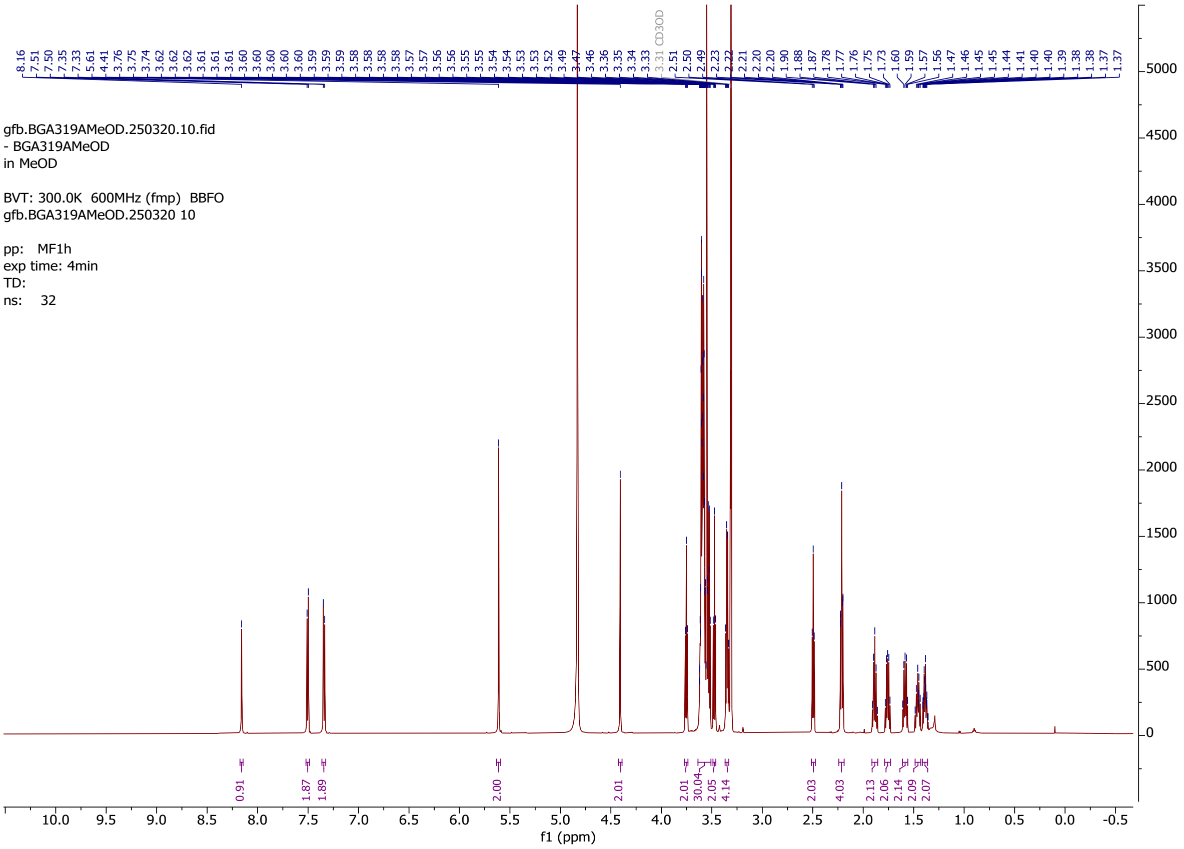

^{13}C NMR (151 MHz, MeOD-d4) of **BG-PEG6-HTL**

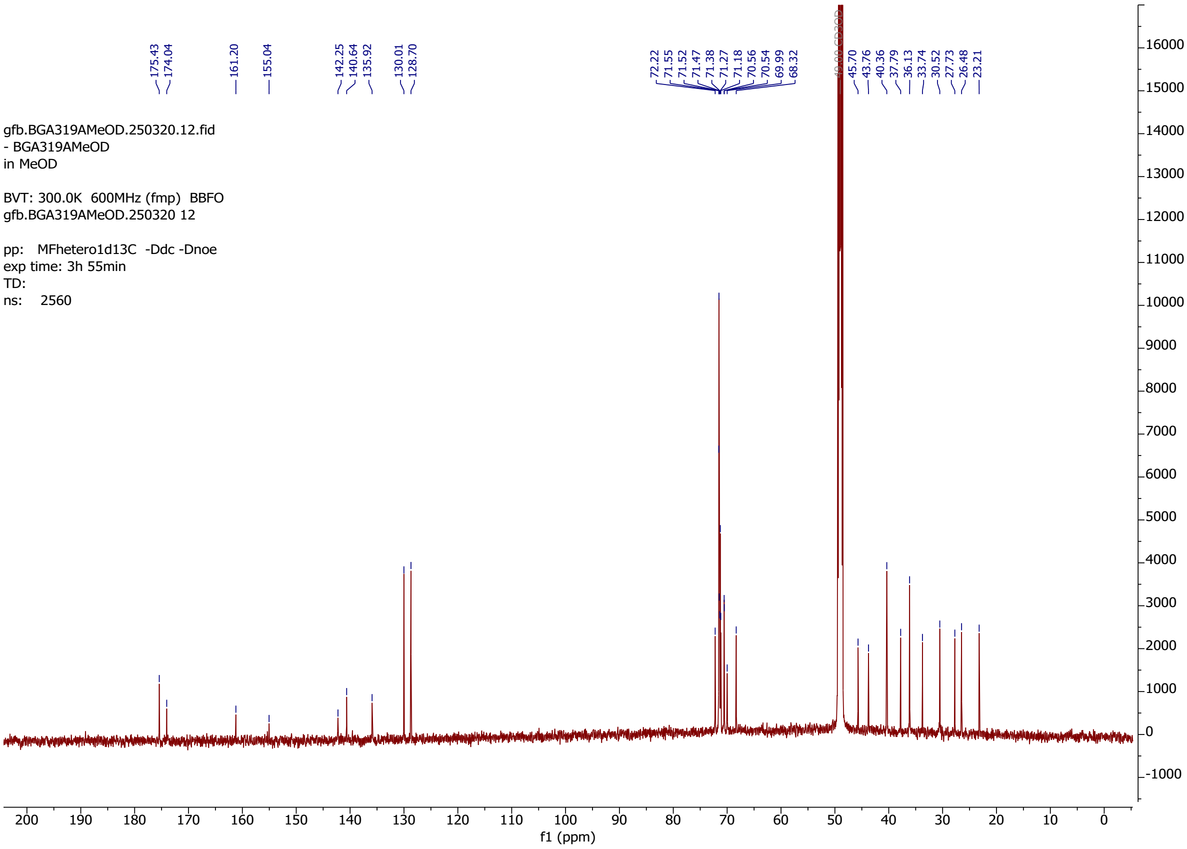

^{19}F NMR (564MHz, MeOD-d4) of **BG-PEG6-HTL**

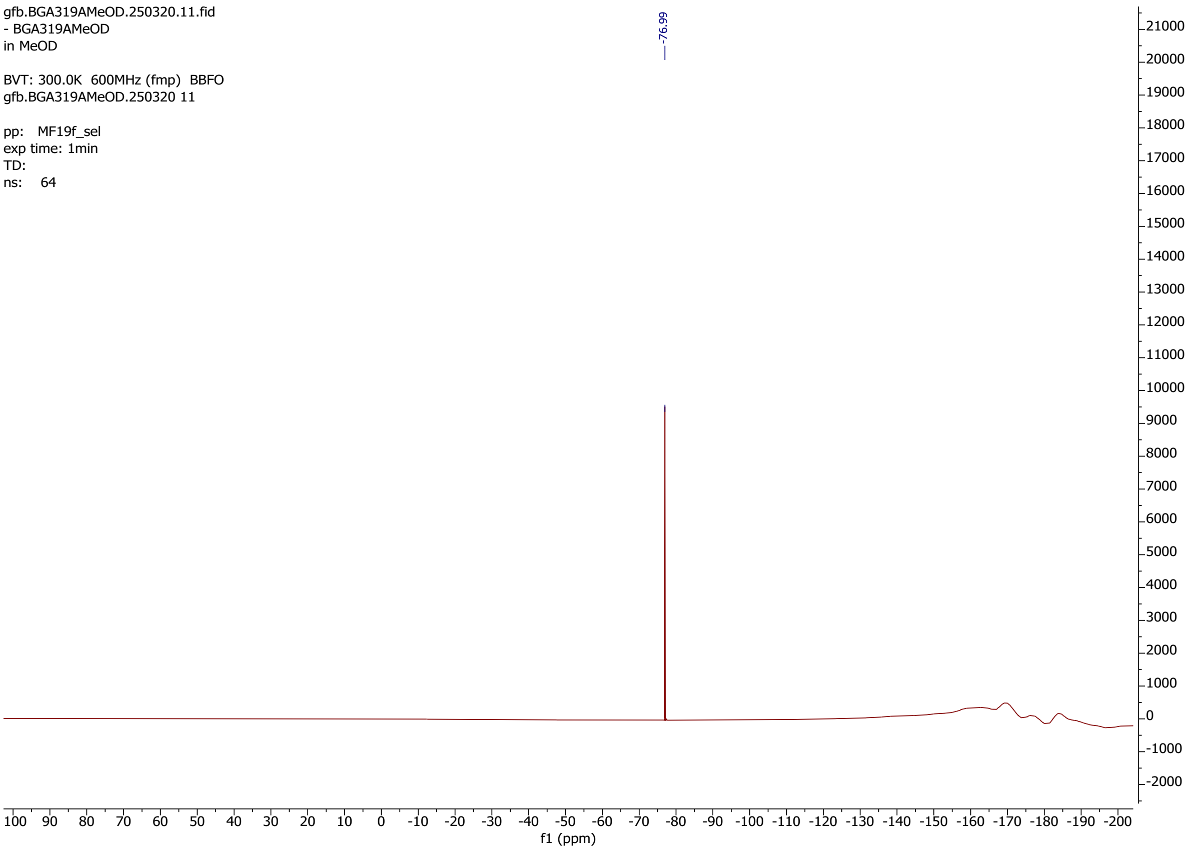

LC-MS of **BG-PEG6-HTL**

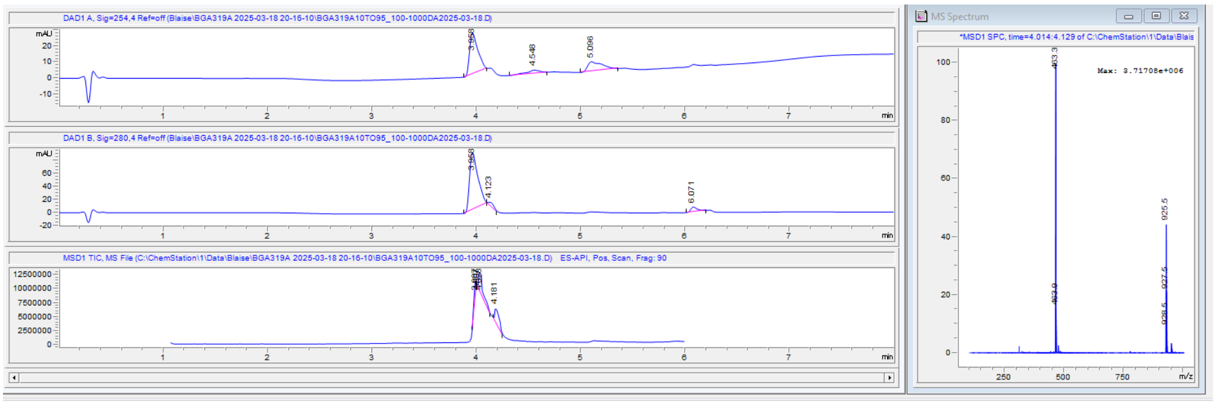

HRMS of **BG-PEG6-HTL**

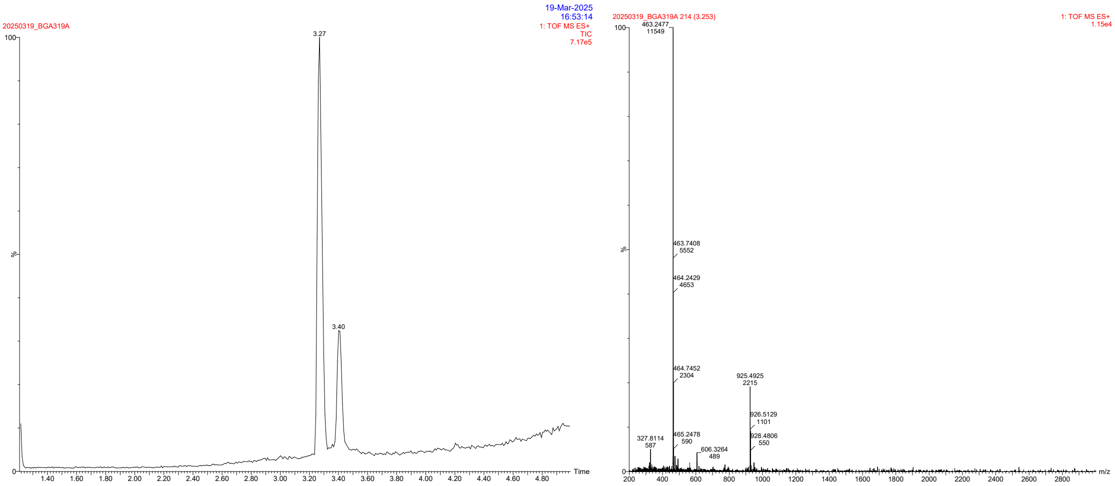

^{1}H NMR (600 MHz, MeOD-d4) of **BG-PEG12-HTL**

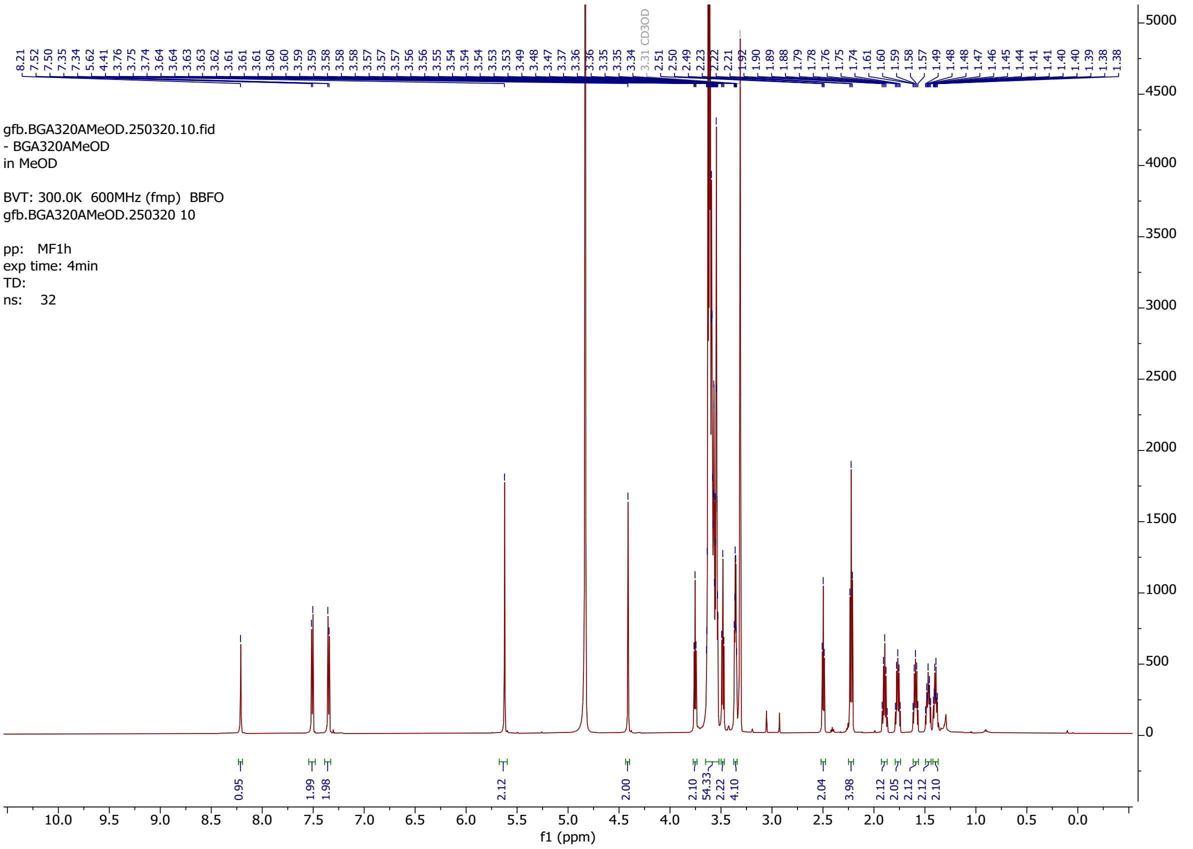

^{13}C NMR (151 MHz, MeOD-d4) of **BG-PEG12-HTL**

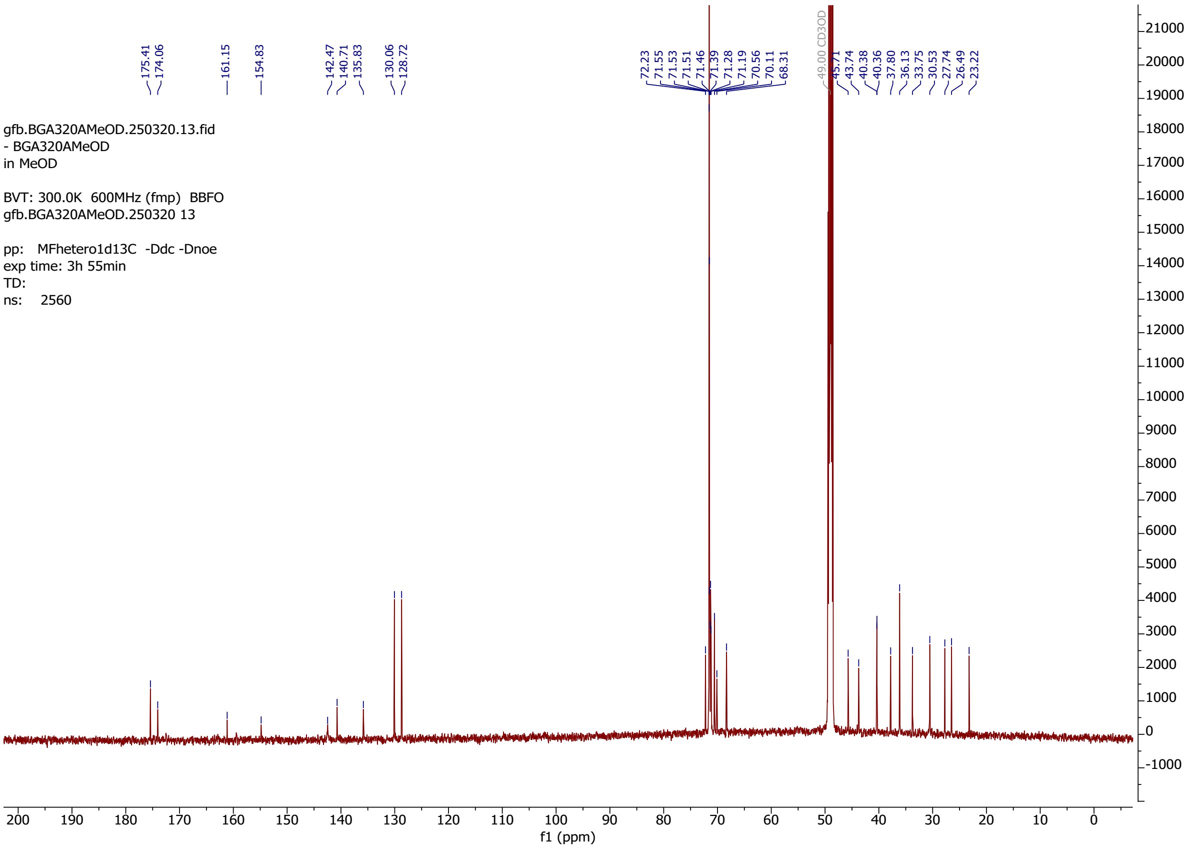

^{19}F NMR (564MHz, MeOD-d4) of **BG-PEG12-HTL**

gfb.BGA320AMeOD.250320.11.fid
- BGA320AMeOD
in MeOD

BVT: 300.0K 600MHz (fmp) BBFO
gfb.BGA320AMeOD.250320 11

pp: MF19f_sel
exp time: 1min
TD:
ns: 64

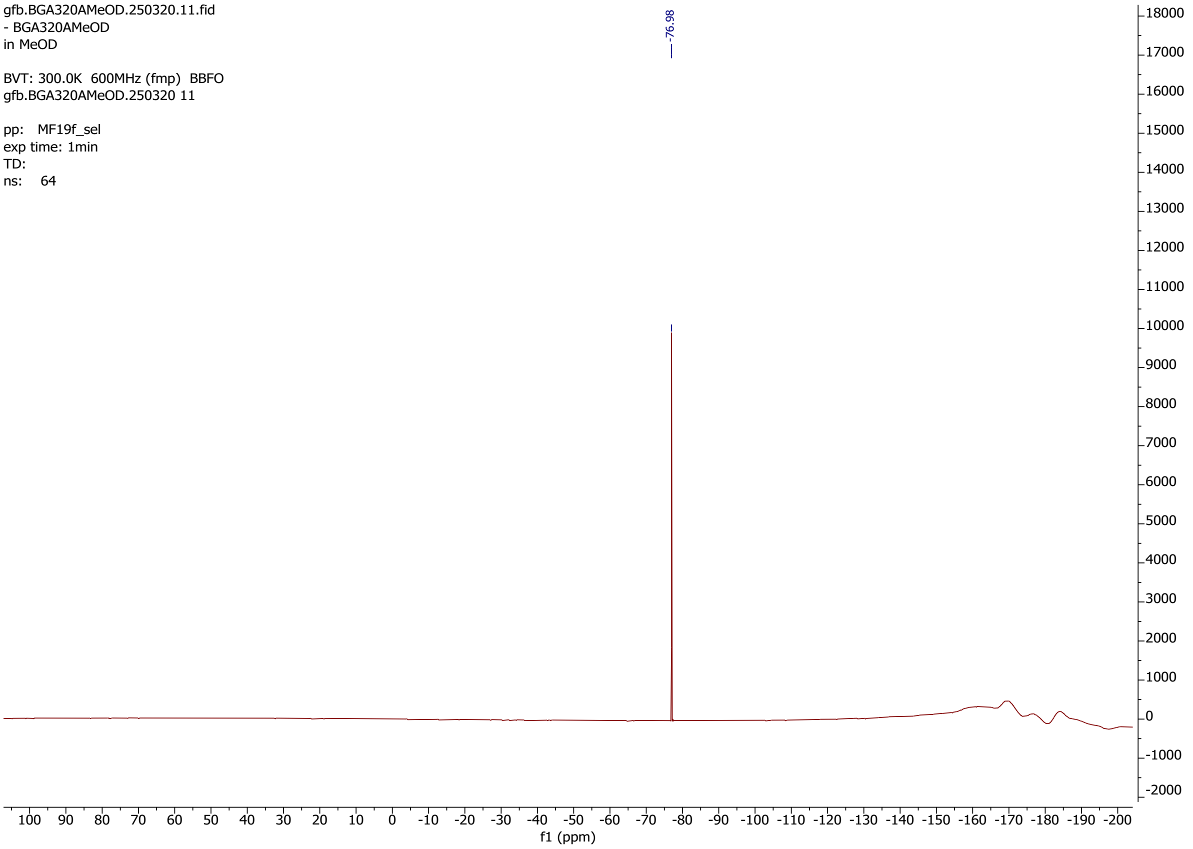

LC-MS of BG-PEG12-HTL

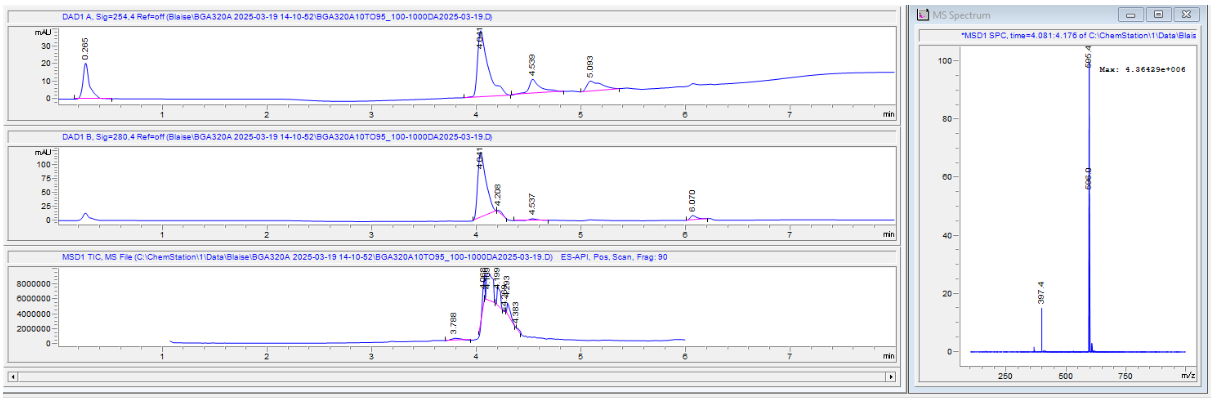

HRMS of BG-PEG12-HTL

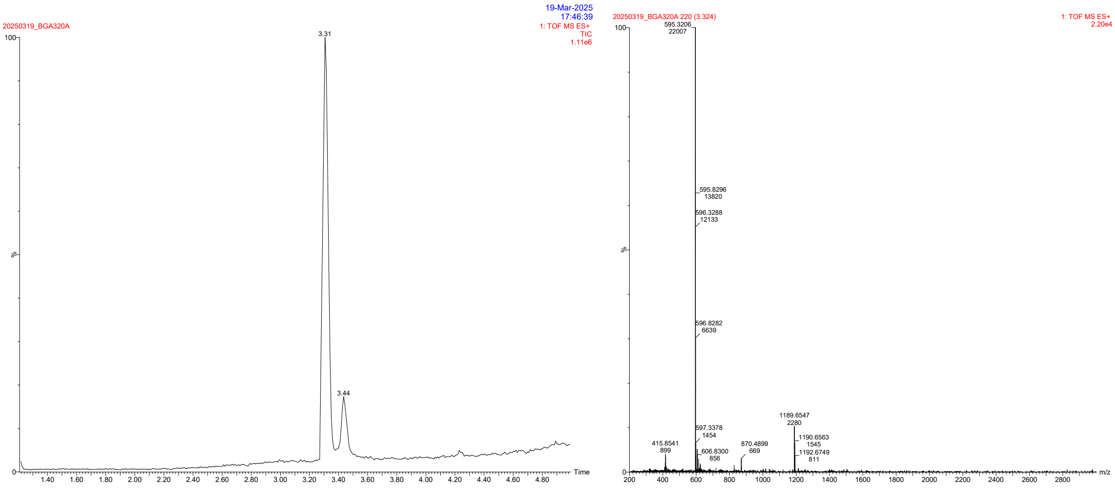

^{1}H NMR (600 MHz, MeOD-d4) of **3**

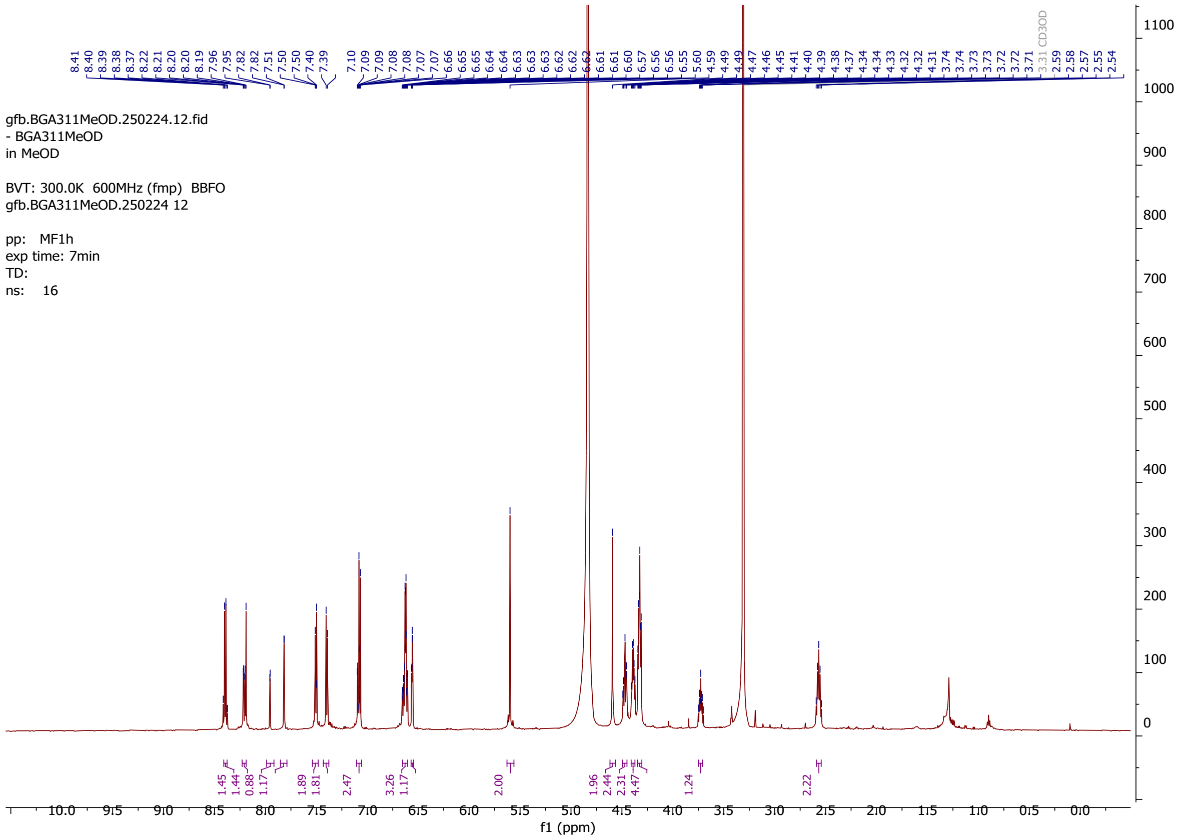

^{19}F NMR (564MHz, MeOD-d4) of **3**

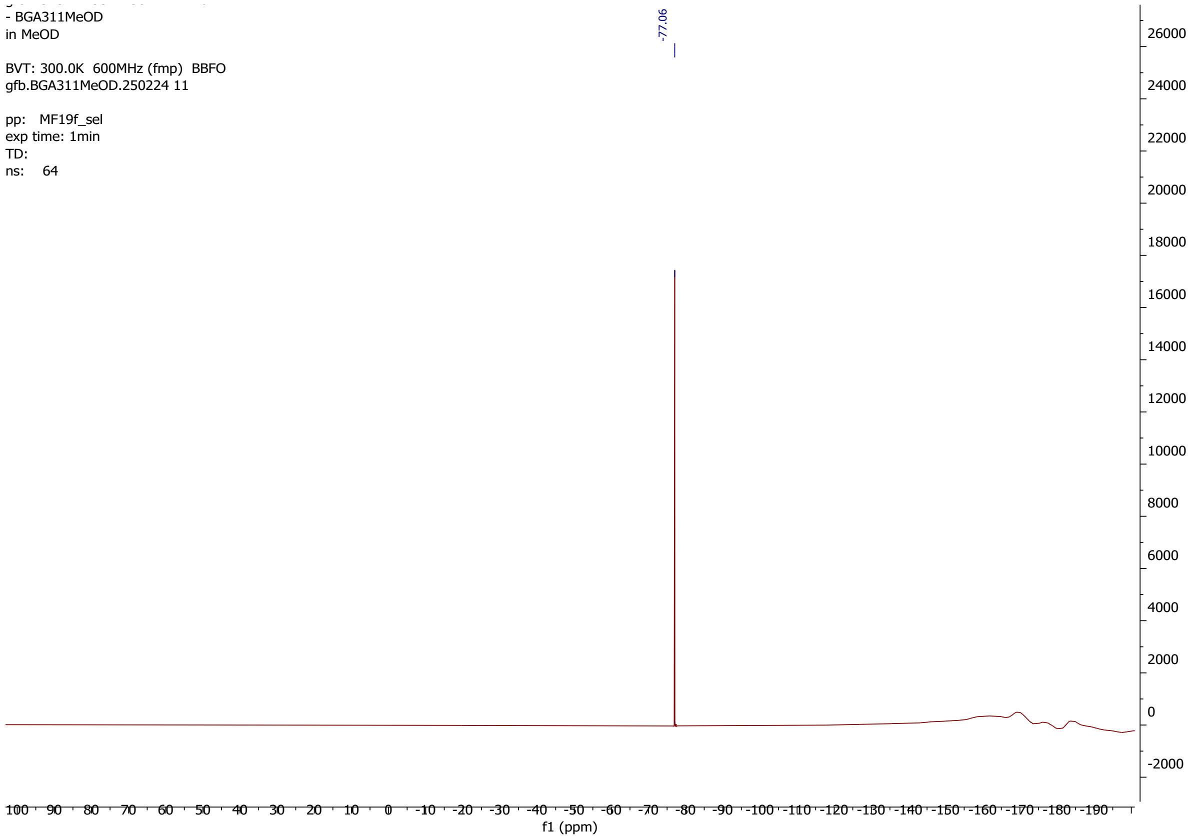

LC-MS of **3**

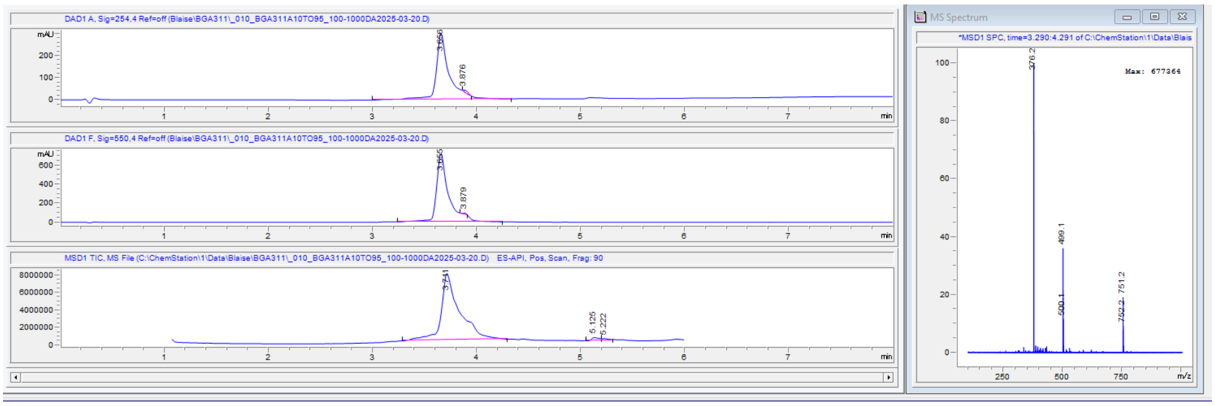

HRMS of **3**

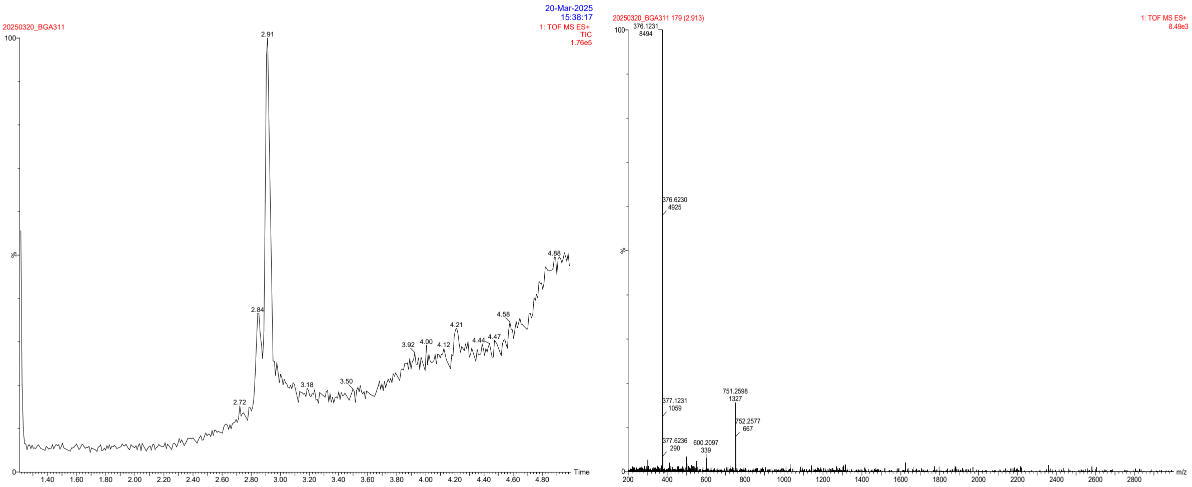

^{1}H NMR (600 MHz, MeOD-d4) of 4

gfb.BGA312MeOD.250225.12.fid
- BGA312MeOD
in MeOD

BVT: 300.0K 600MHz (fmp) BBFO
gfb.BGA312MeOD.250225 12

pp: MF1h
exp time: 7min
TD:
ns: 16

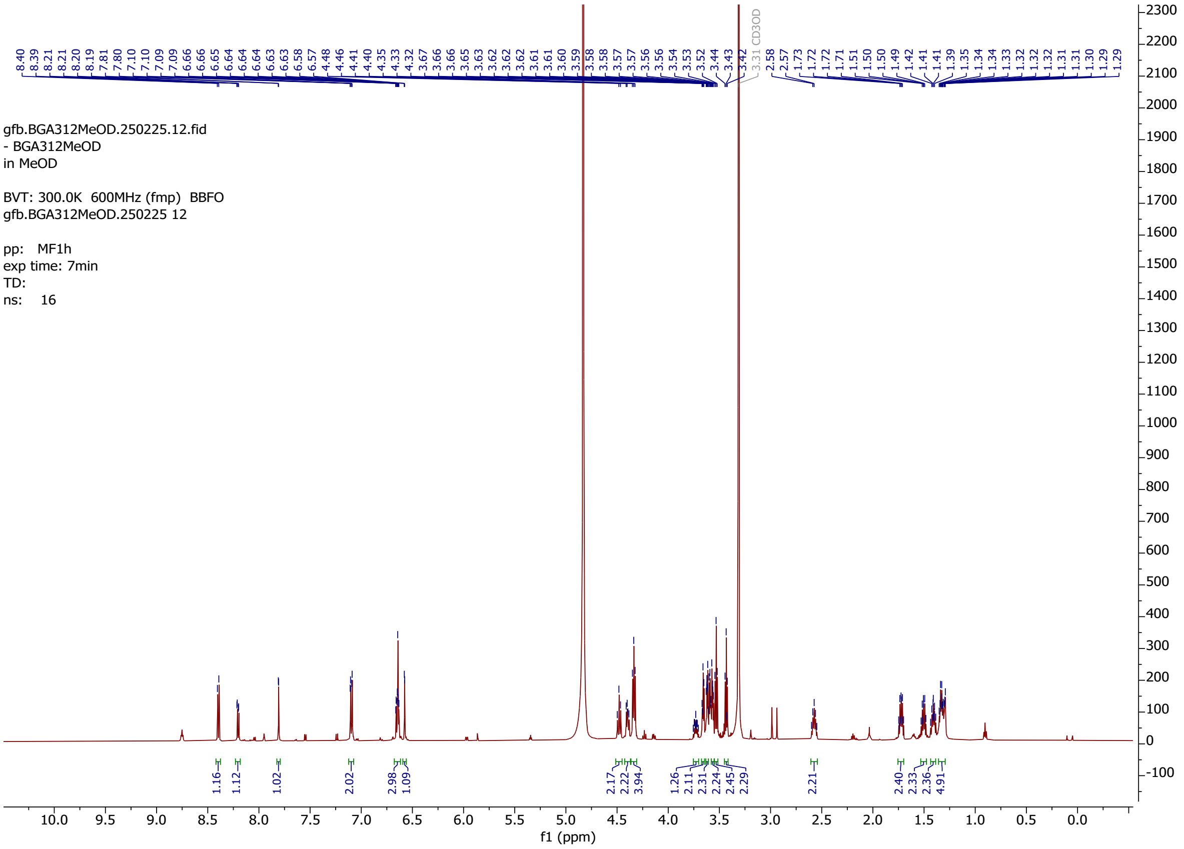

^{19}F NMR (564MHz, MeOD-d4) of 4

gfb.BGA312MeOD.250225.11.fid
- BGA312MeOD
in MeOD

BVT: 300.0K 600MHz (fmp) BBFO
gfb.BGA312MeOD.250225 11

pp: MF19f_sel
exp time: 1min
TD:
ns: 64

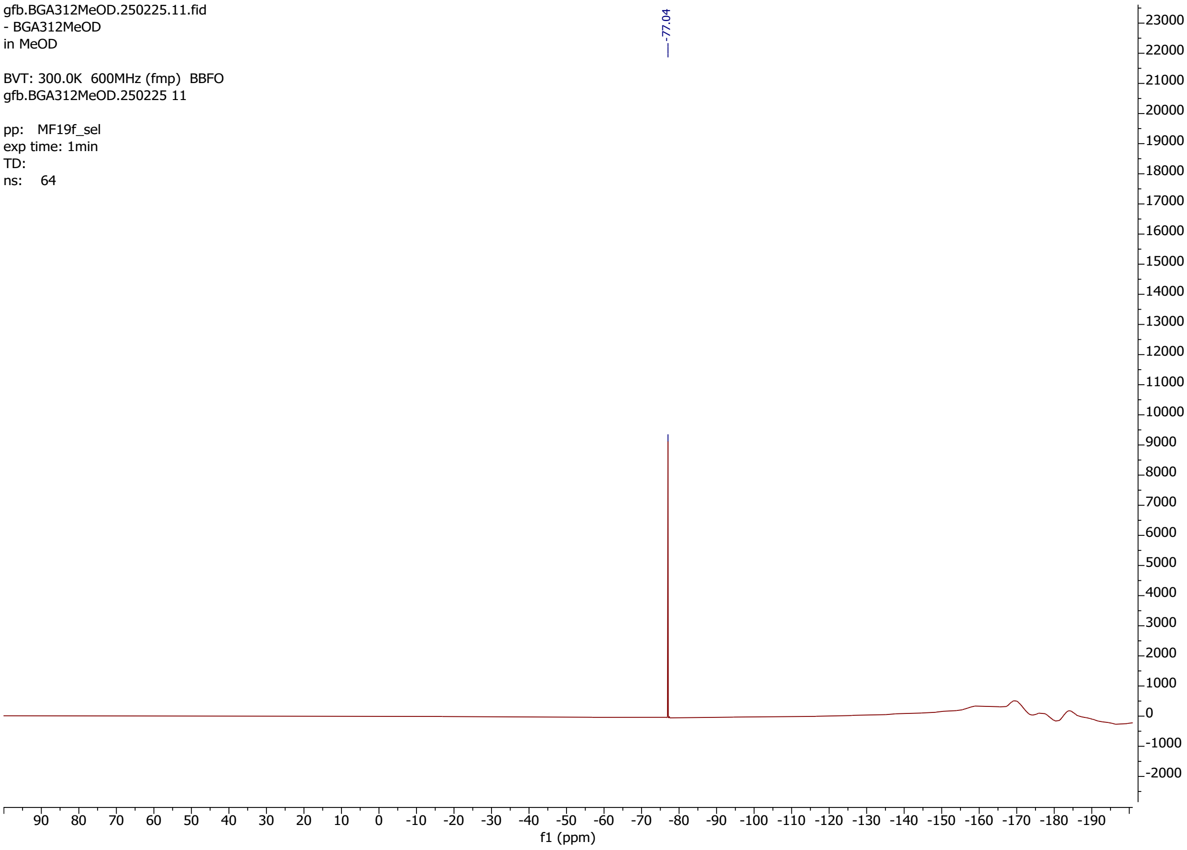

LC-MS of **4**

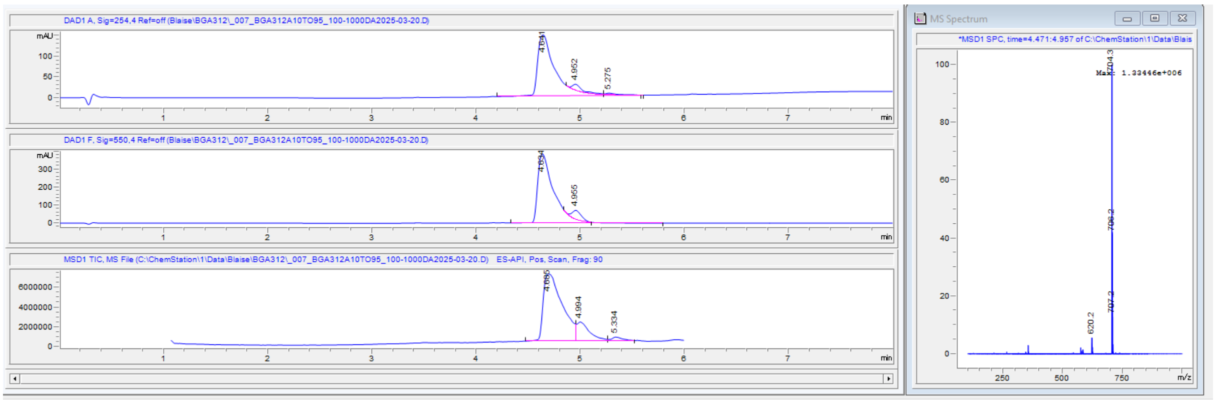

HRMS of **4**

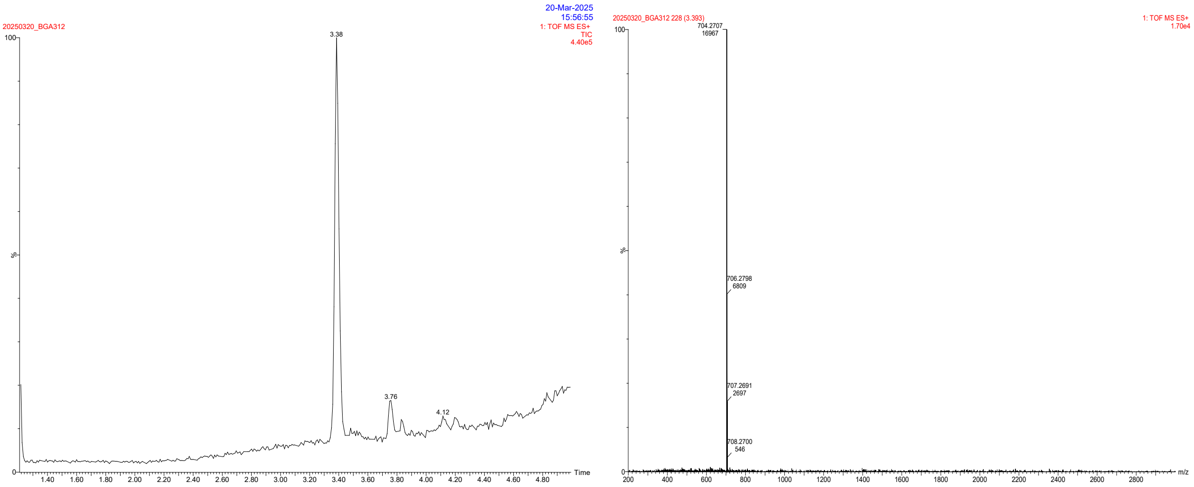

^{1}H NMR (600 MHz, DMSO-d6) of 5

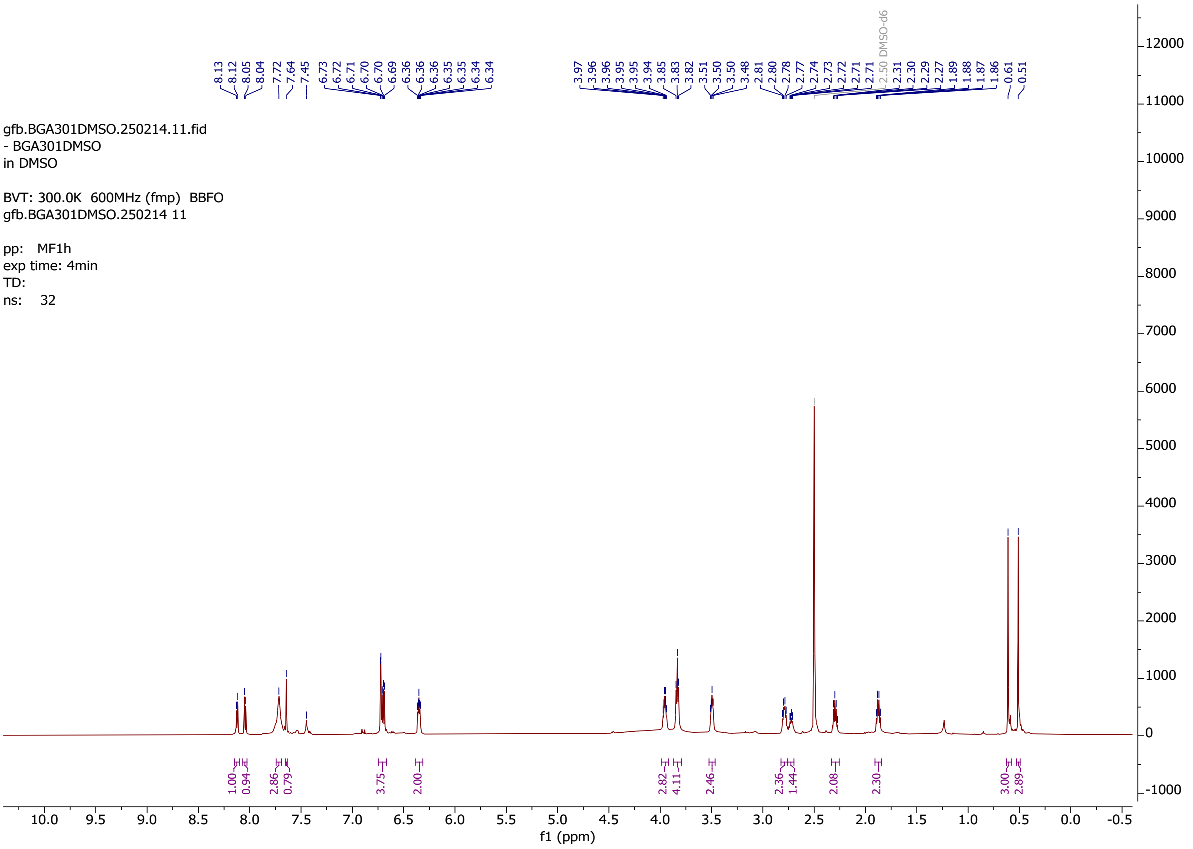

^{13}C NMR (151 MHz, DMSO-d6) of 5

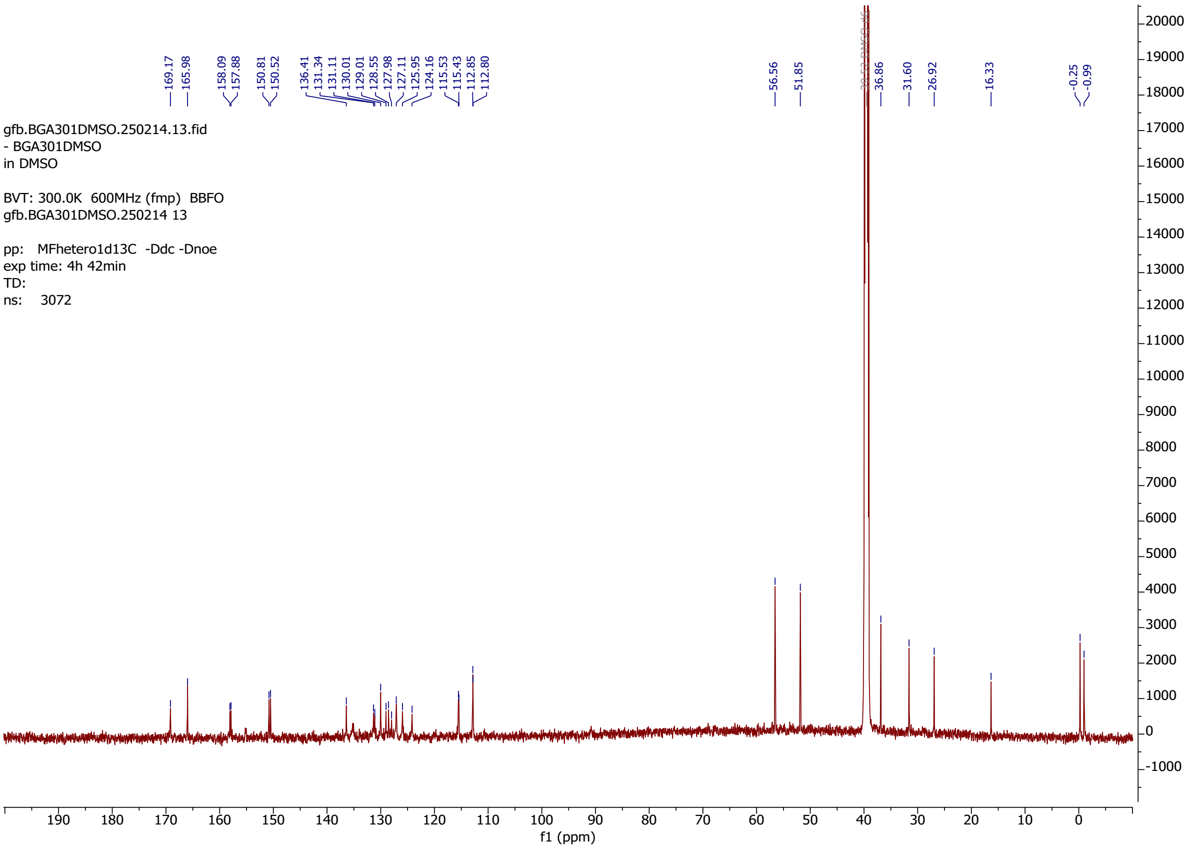

^{19}F NMR (564 MHz, DMSO-d6) of **5**

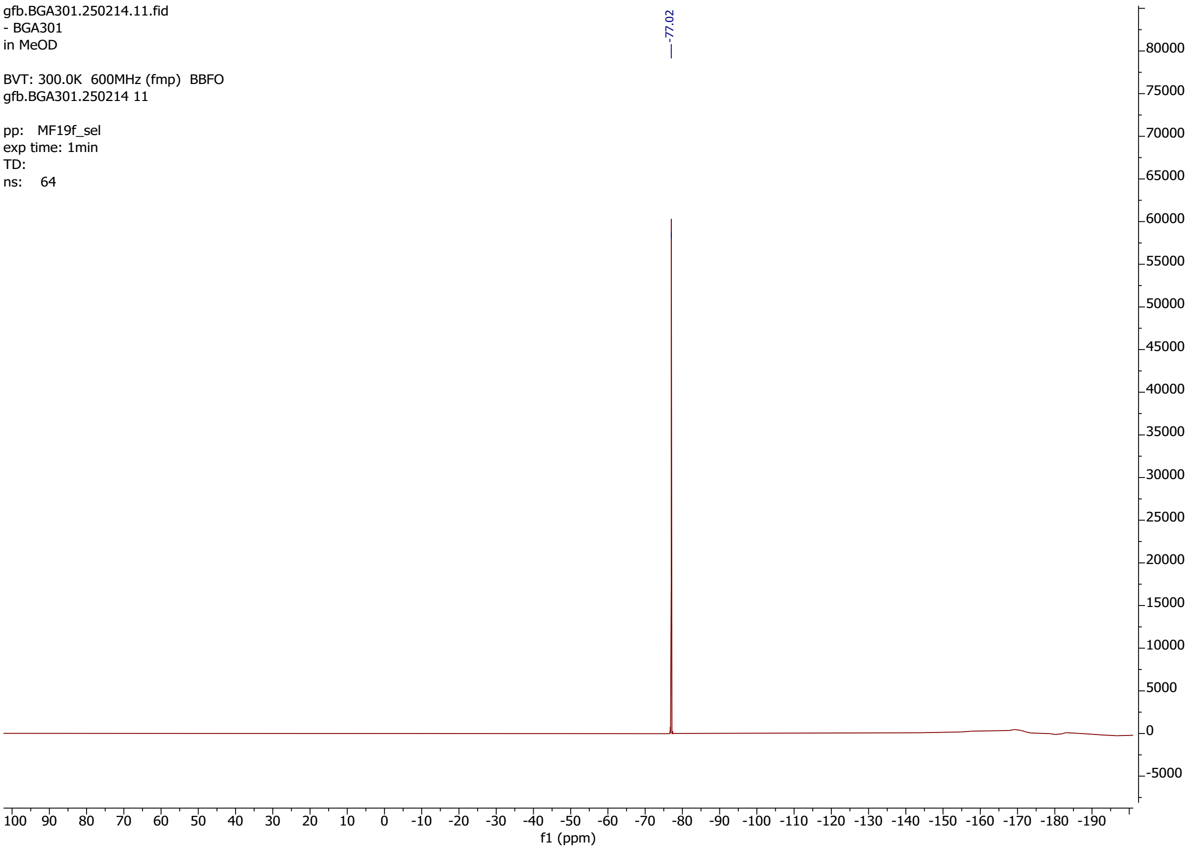

LC-MS of **5**

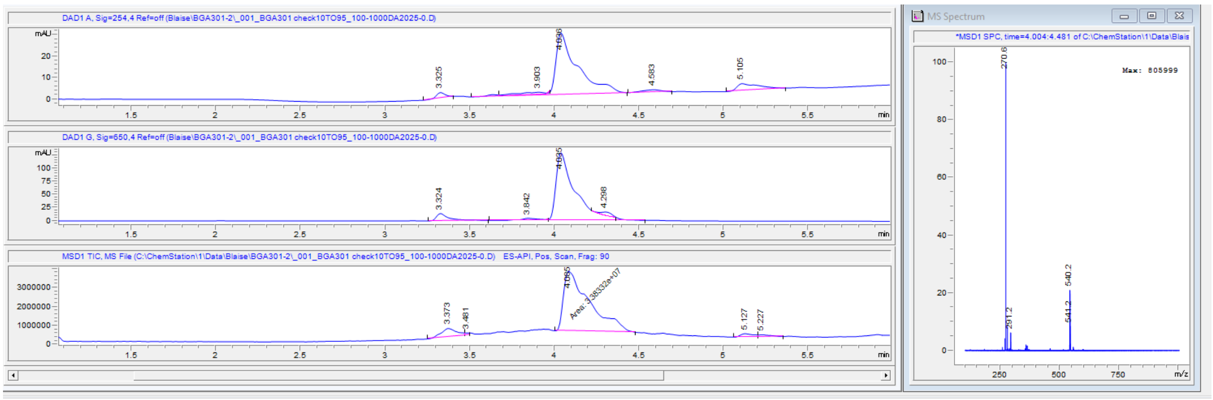

HRMS of **5**

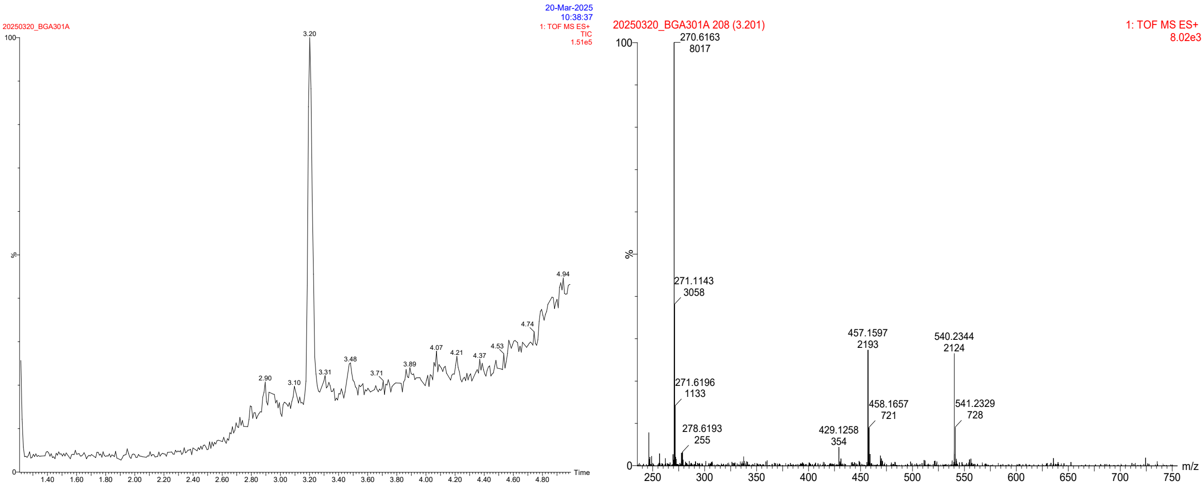

^{1}H NMR (600 MHz, MeOD-d4) of **6**

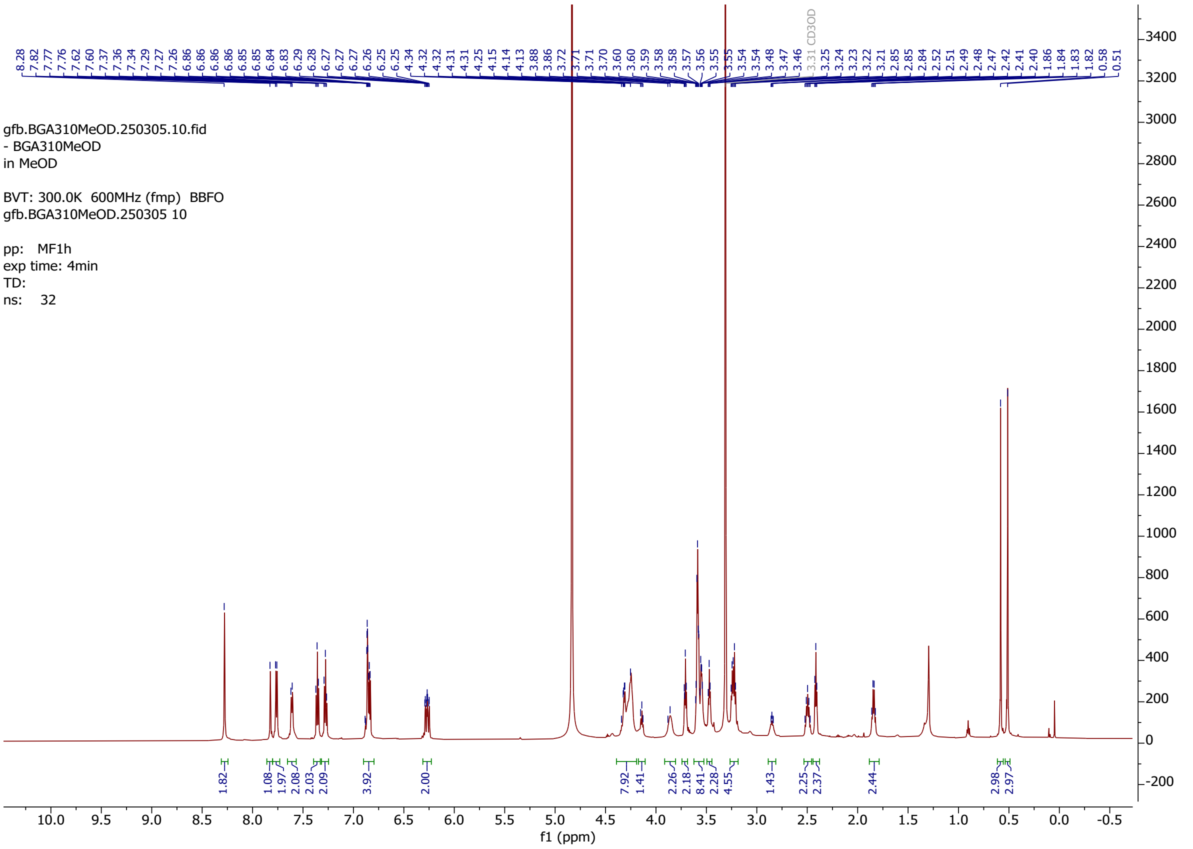

^{19}F NMR (564MHz, MeOD-d4) of **6**

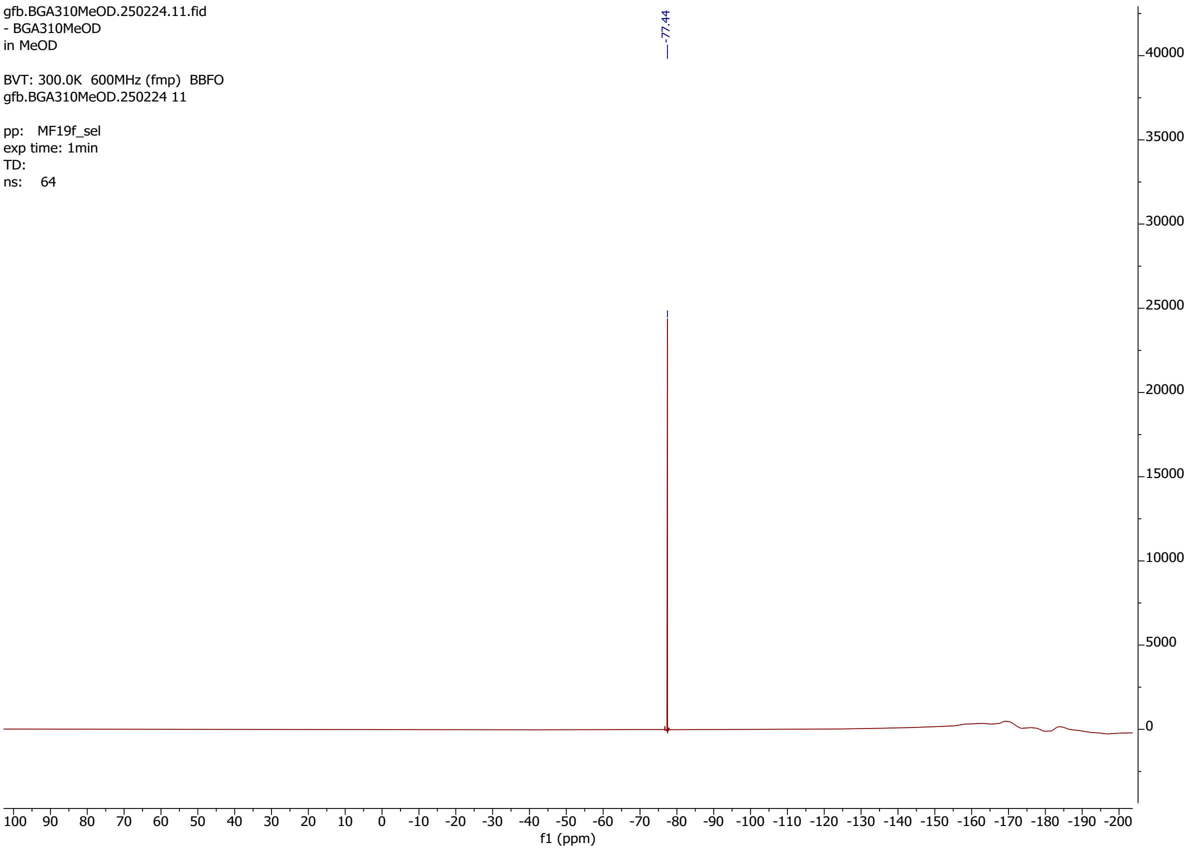

LC-MS of **6**

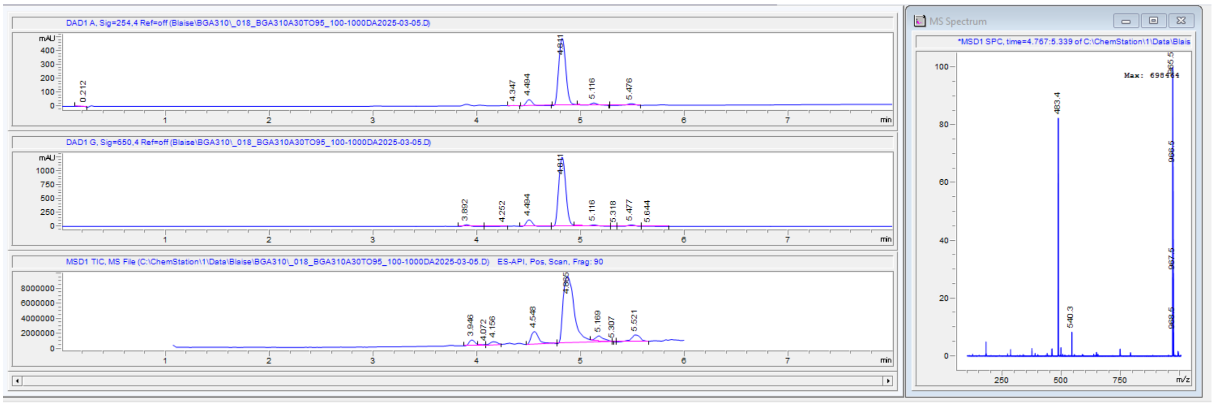

HRMS of **6**

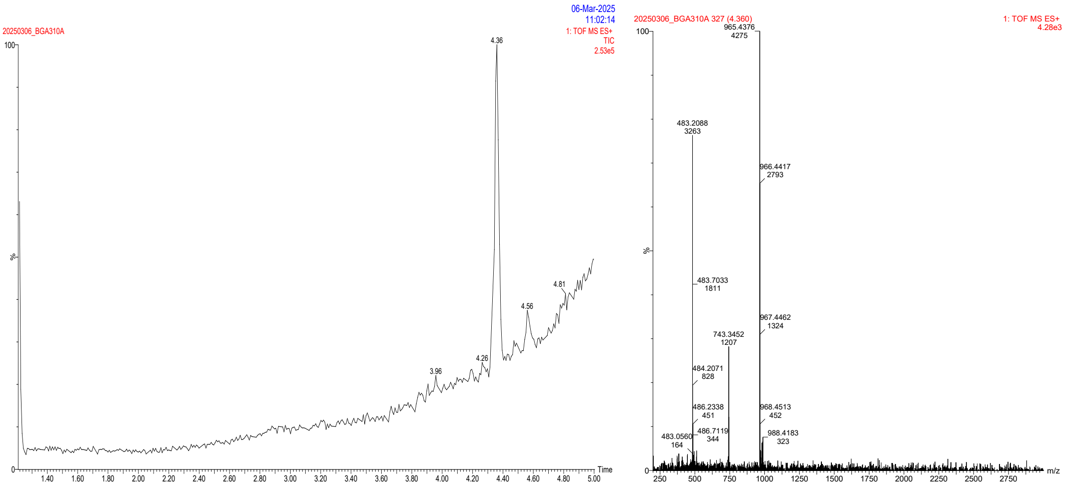

^{1}H NMR (600 MHz, MeOD-d4) of **7**

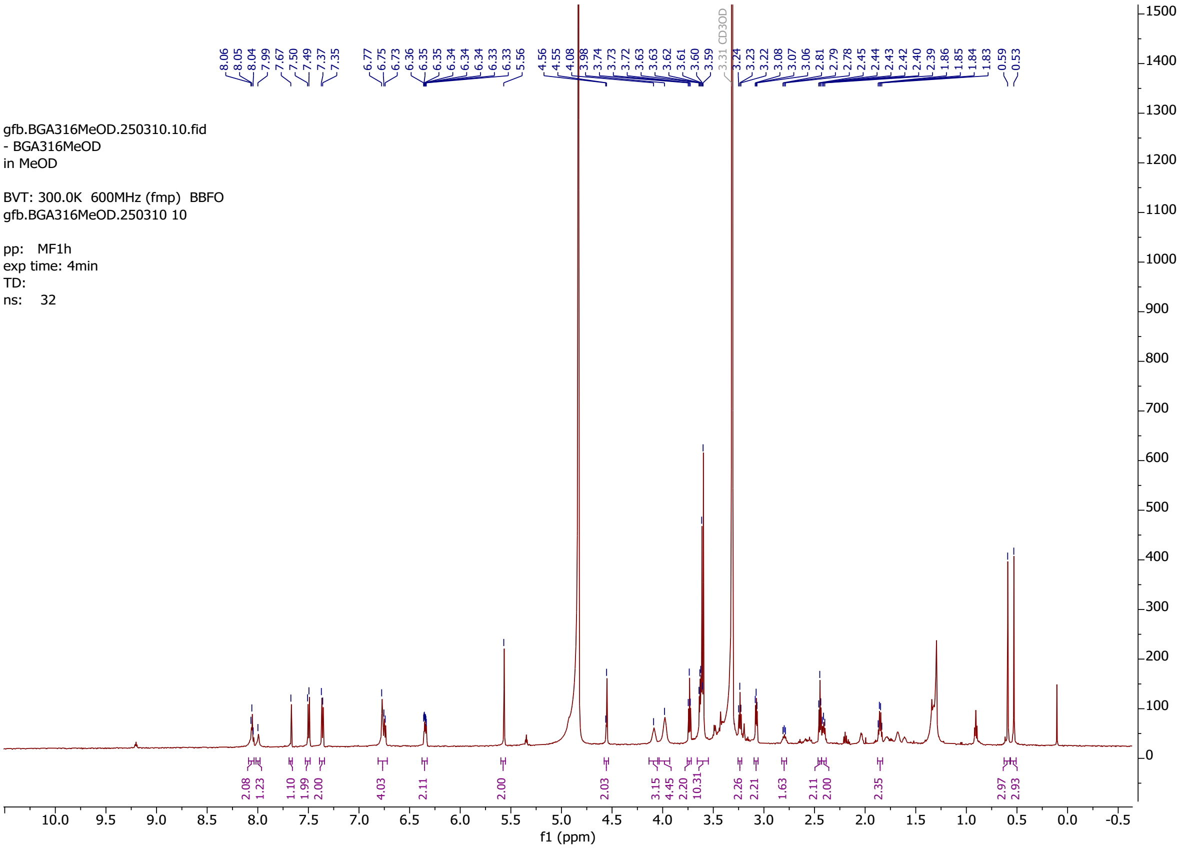

^{19}F NMR (564MHz, MeOD-d4) of **7**

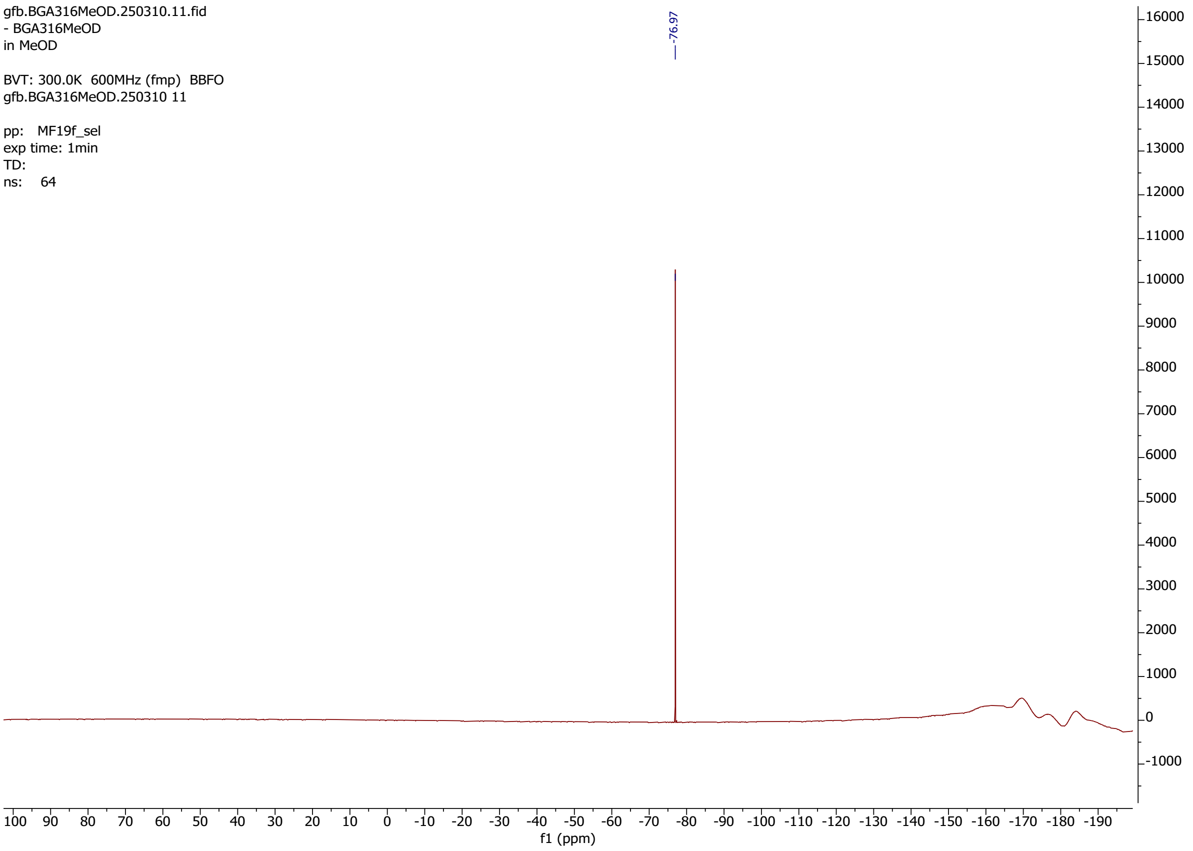

LC-MS of 7

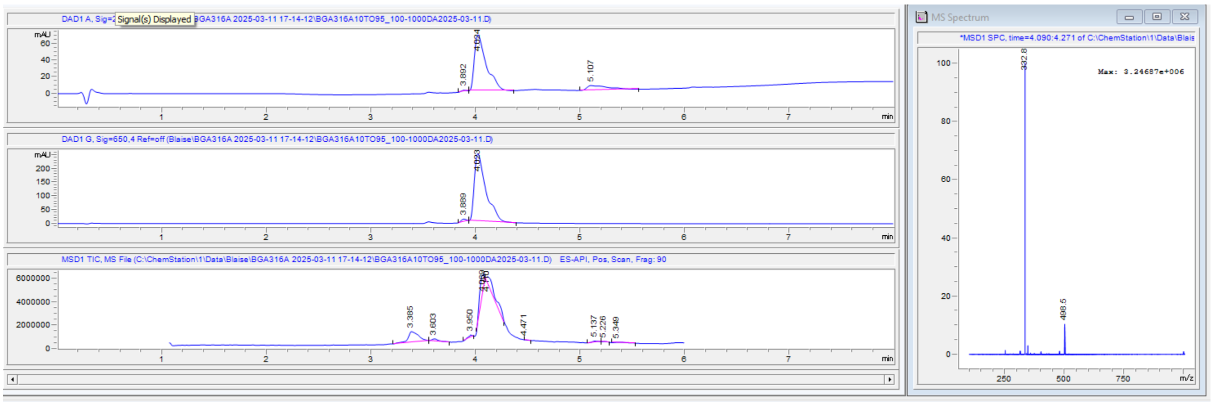

HRMS of 7

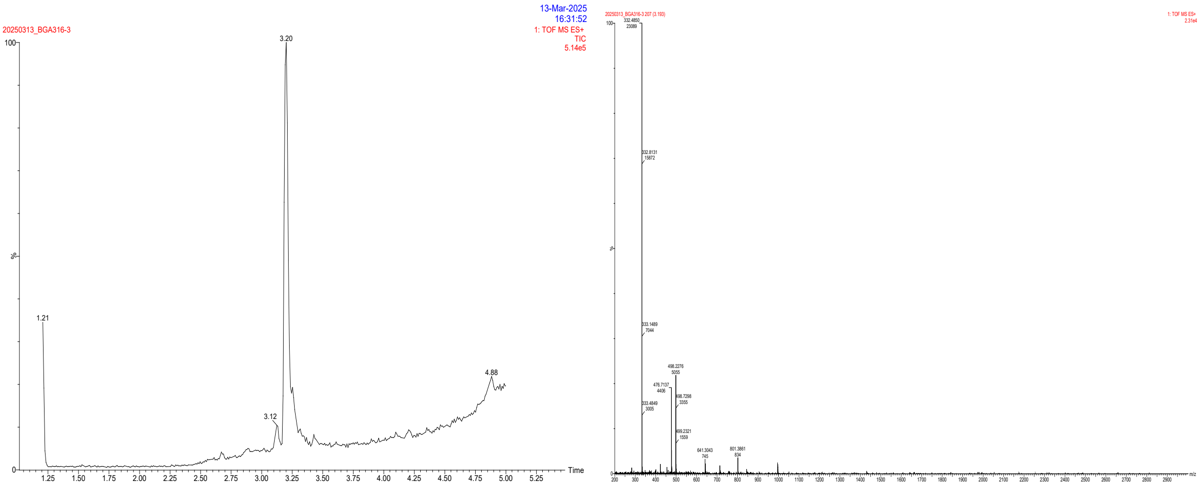

^{1}H NMR (600 MHz, MeOD-d4) of **8**

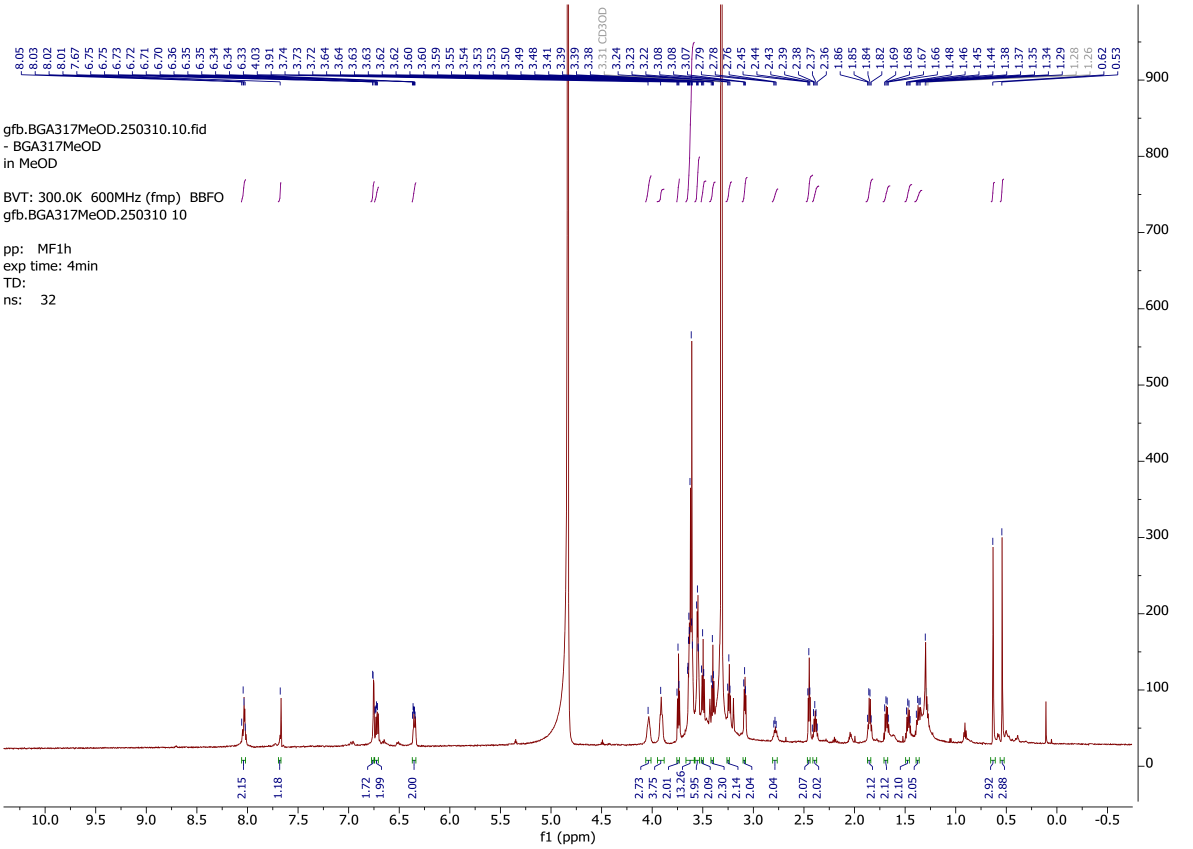

^{19}F NMR (564MHz, MeOD-d4) of **8**

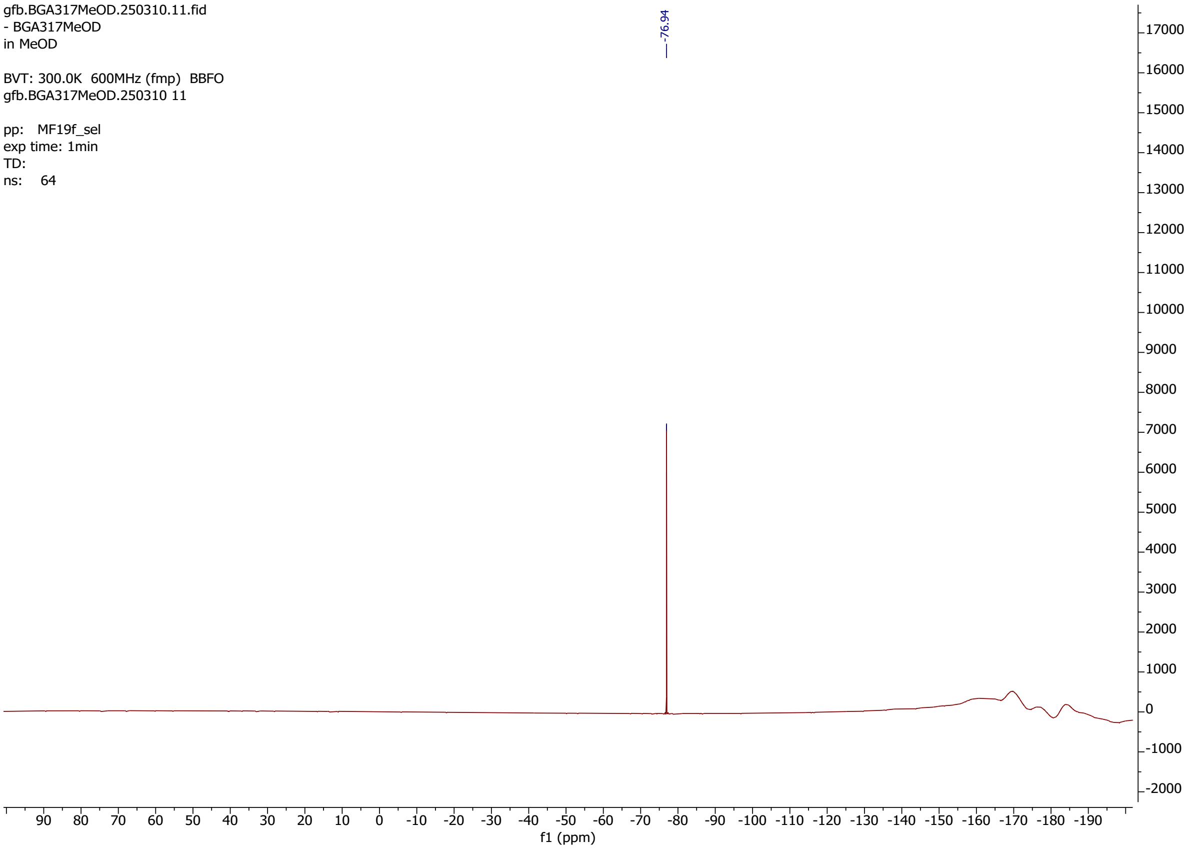

LC-MS of **8**

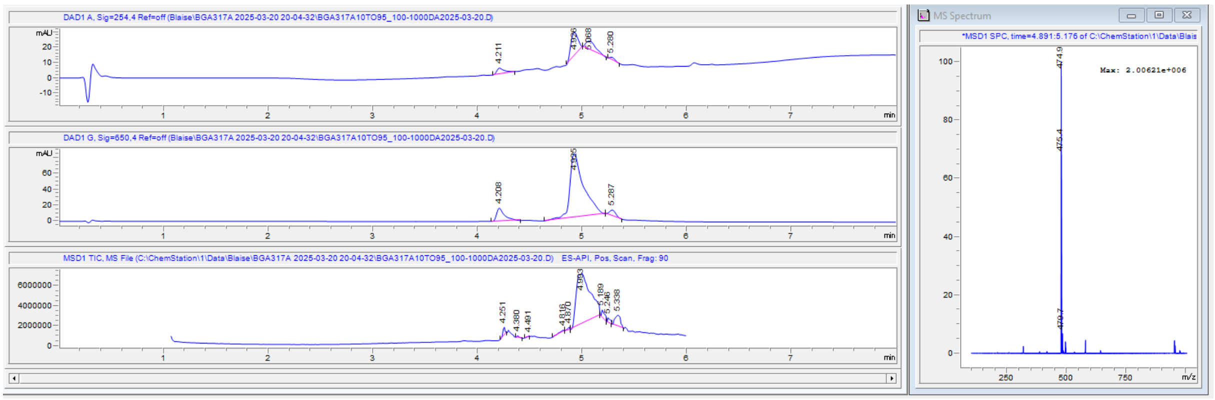

HRMS of **8**

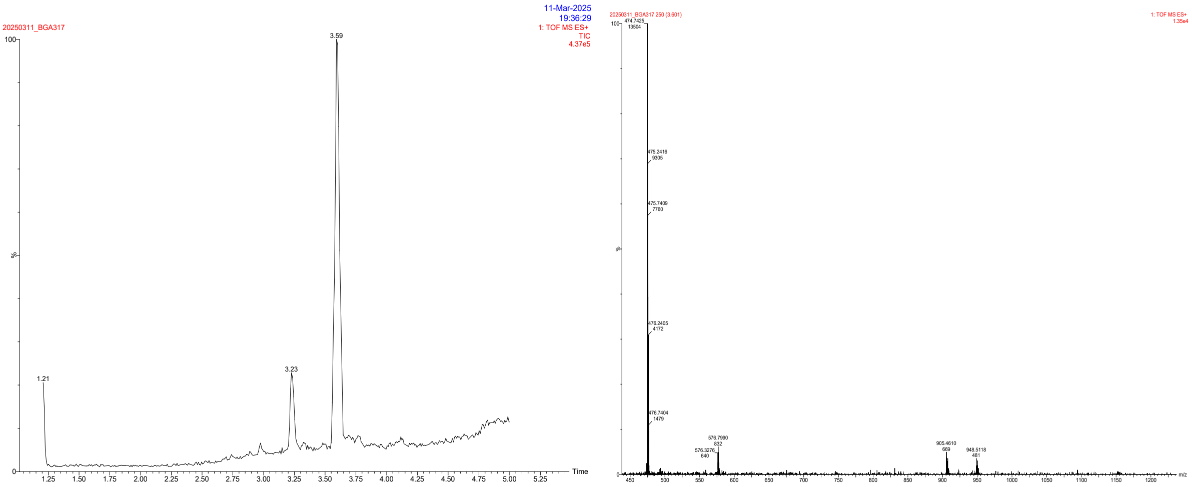

^{1}H NMR (600 MHz, MeOD-d4) of **MT36**

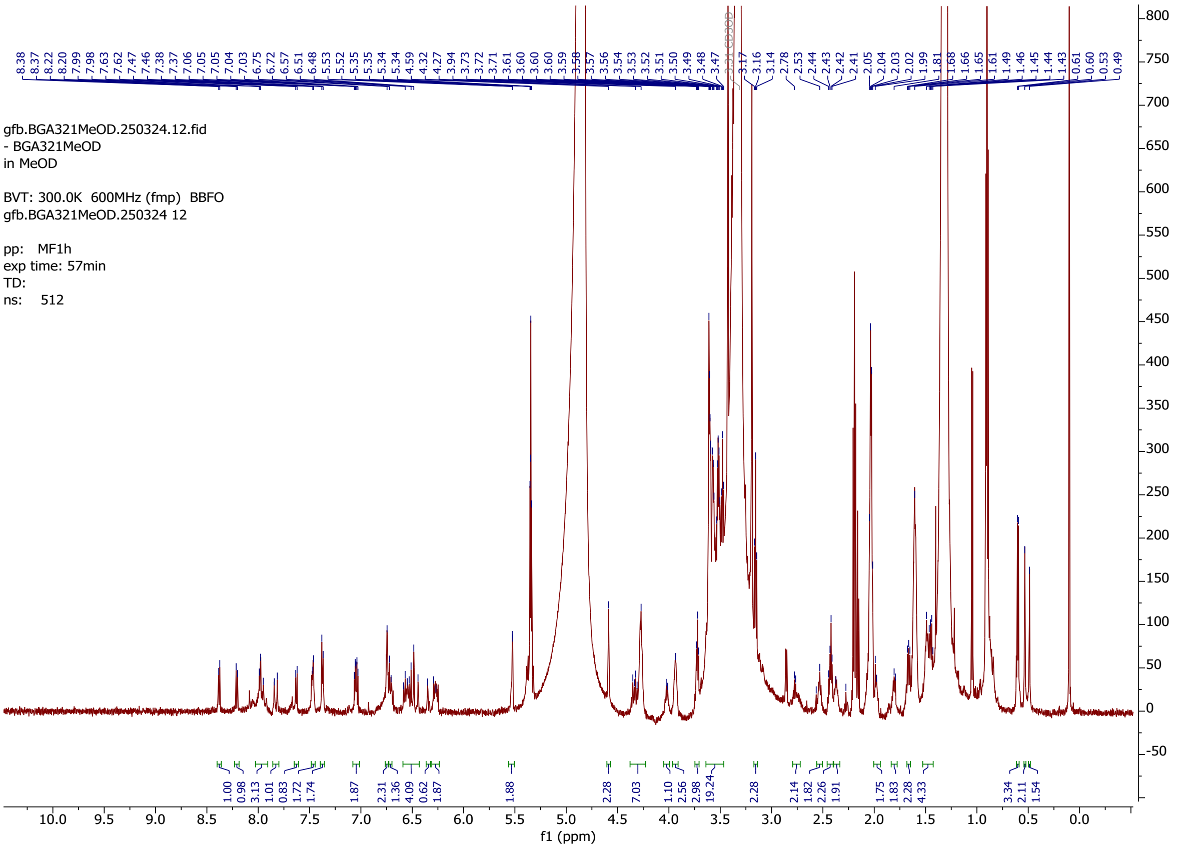

^{19}F NMR (564MHz, MeOD-d4) of **MT36**

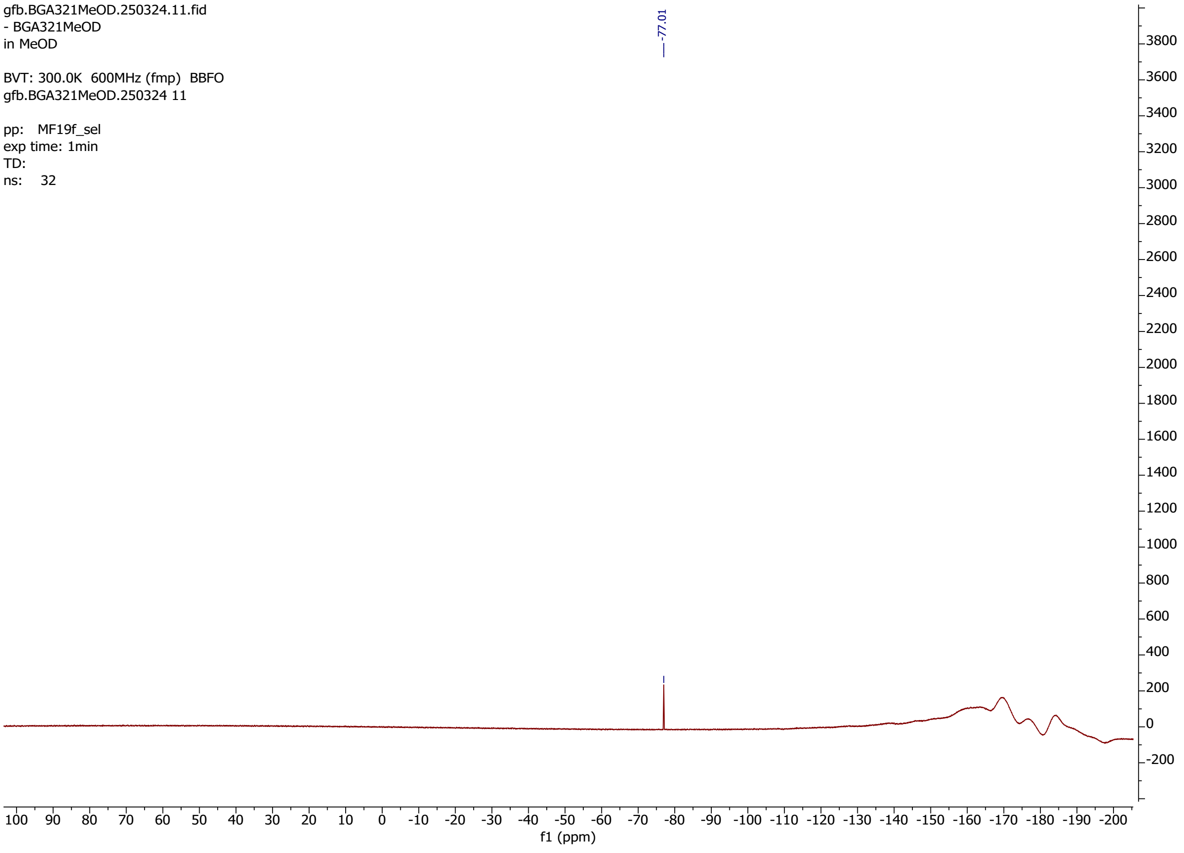

LC-MS of **MT36**

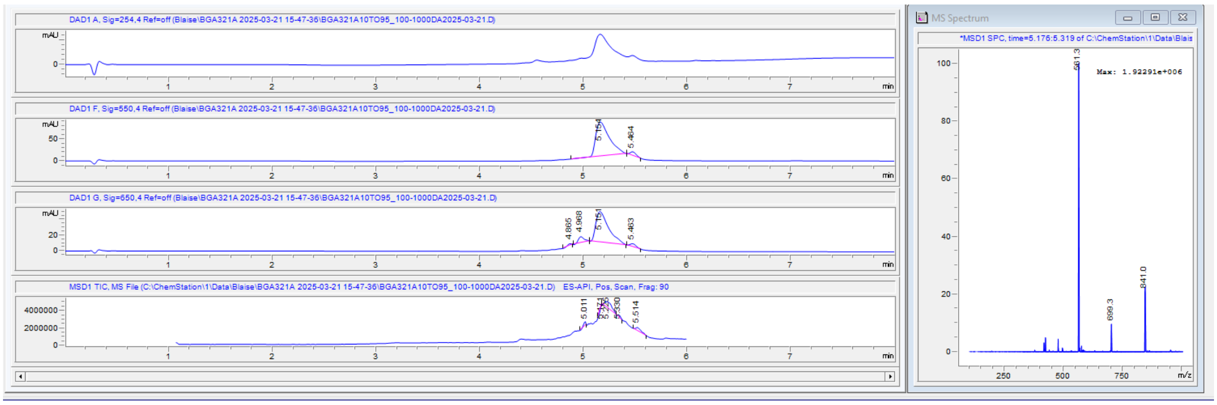

HRMS of **MT36**

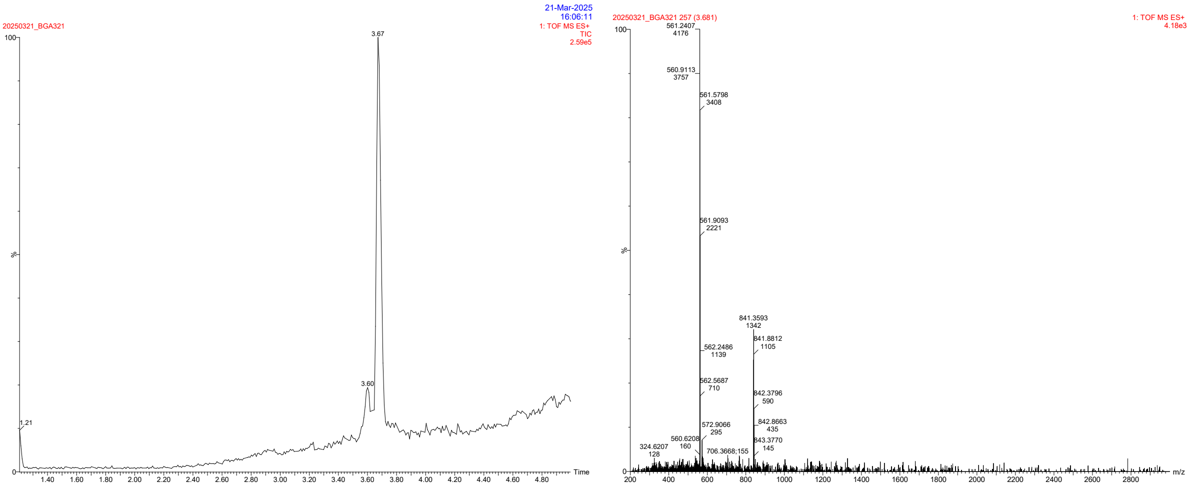

^{1}H NMR (600 MHz, MeOD-d4) of MT37

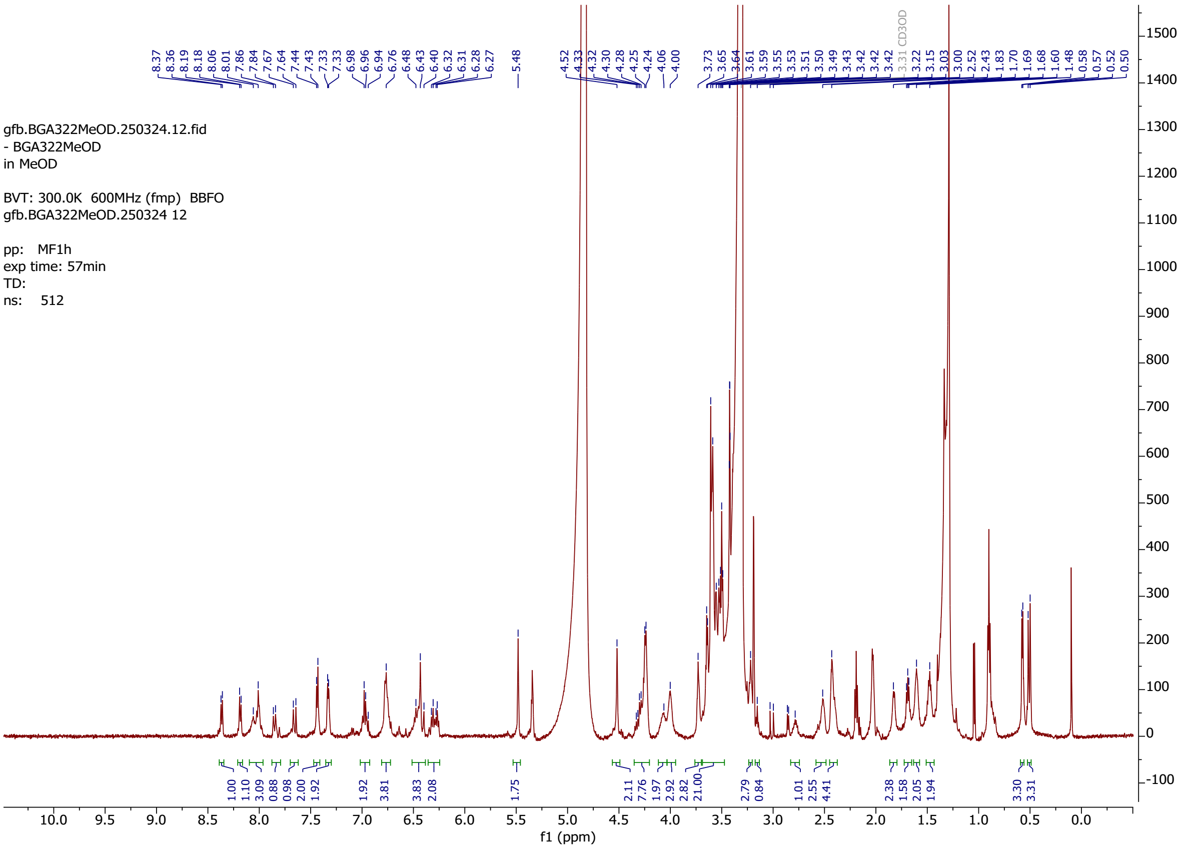

^{19}F NMR (564MHz, MeOD-d4) of MT37

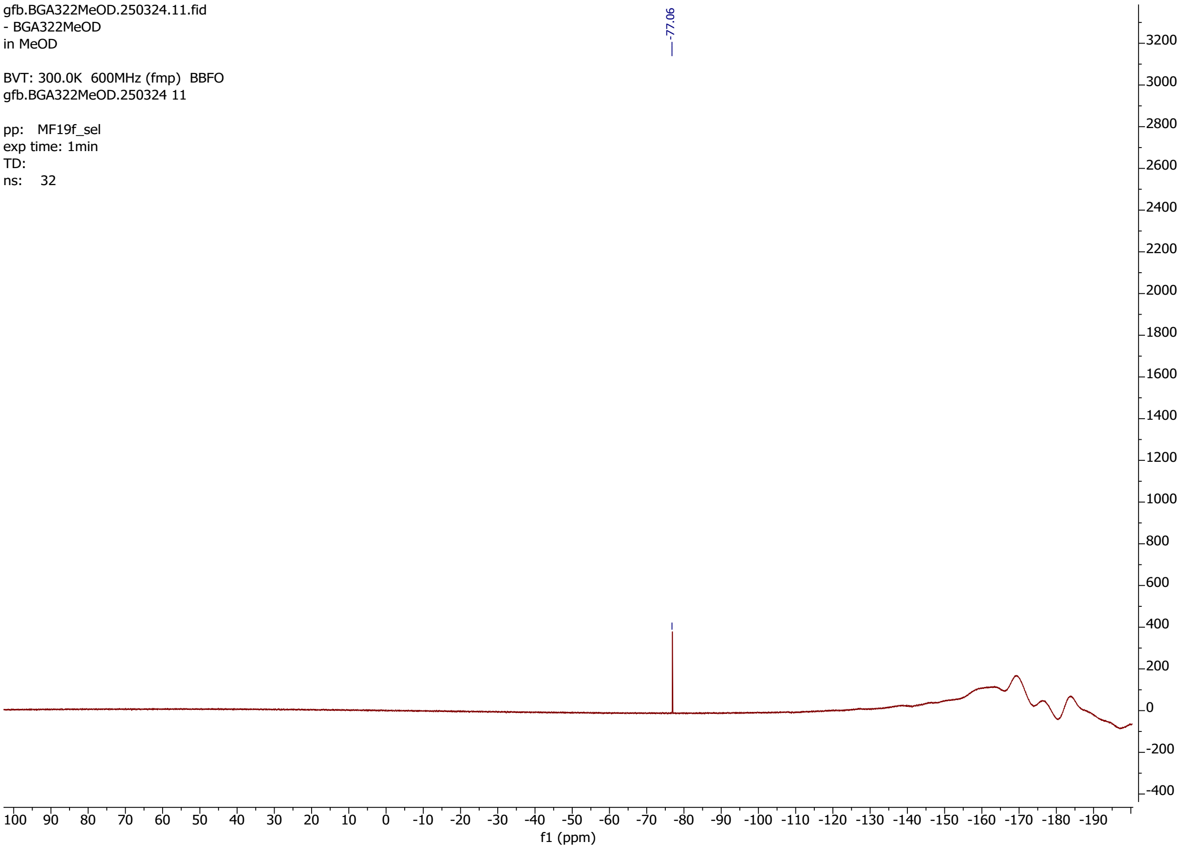

LC-MS of **MT37**

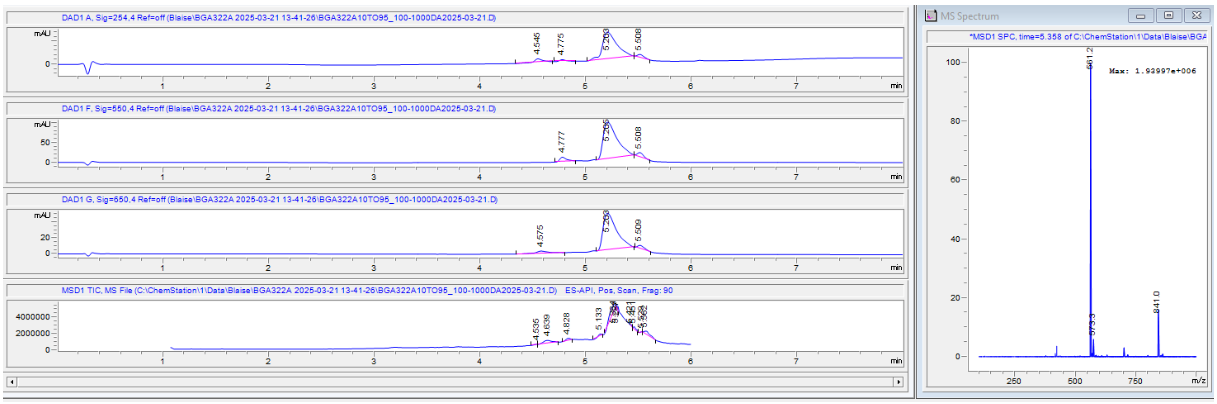

HRMS of **MT37**

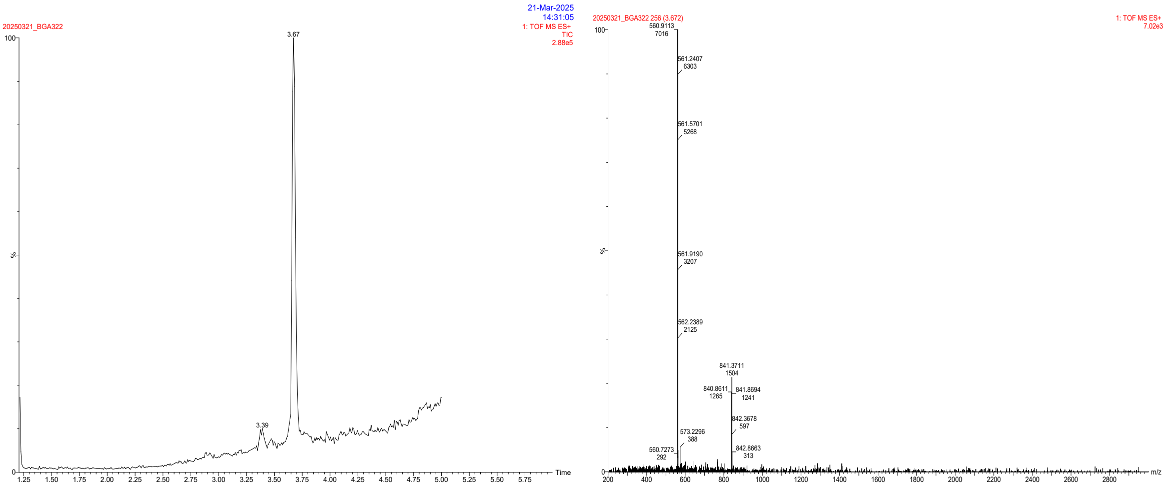

Methods

LC-MS

UPLC-UV/Vis for purity assessment was performed on an Agilent 1260 Infinity II LC System equipped with Agilent SB-C18 column (1.8 µm, 2.1 × 50 mm). Buffer A: 0.1% FA in H_2O Buffer B: 0.1% FA acetonitrile. The typical gradient was from 5% B for 1.0 min → gradient to 95% B over 5 min → 95% B for 1.0 min with 0.6 mL/min flow or from 30% B for 1.0 min → gradient to 95% B over 5 min. Retention times (t_R) are given in minutes (min). Chromatograms were imported into Graphpad Prism 9.3 and purity was determined by calculating AUC ratios.

High resolution mass spectrometry

For HRMS, samples were analyzed on Orbitrap Fusion mass spectrometer (Thermo Fisher Scientific). MS scans were acquired in a range of 350 to 1500 m/z. MS1 scans were acquired in the Orbitrap with a mass resolution of 120,000 with an AGC target value of 4e5 and 50 ms injection time. MS2 scans were acquired in the ion trap with an AGC target value of 1e4 and 35 ms injection time. Precursor ions with charge states 2-4 were isolated with an isolation window of 1.6 m/z and 40 sec dynamic exclusion. Precursor ions were fragmented using higher-energy collisional dissociation (HCD) with 30% normalized collision energy. The monoisotopic masses of the molecules were obtained by deconvoluting the mass spectrometry raw data using xtract algorithm that is built into FreeStyle software version 1.6 (Thermo Fisher scientific).

Protein mass spectrometry

Labelling substrates were dissolved in DMSO to a concentration of 1 mM and diluted in activity buffer (containing: 50 mM NaCl, 50 mM HEPES, pH = 7.3 + 4 µg/mL BSA) to 20 µM. Protein was diluted in activity buffer to a concentration of 2 µM. 25 µL of each protein and labelling agent were combined in a mass spec vial and allowed to incubate at r.t. for 1 h, before full protein mass was in a QTof MS acquired. In case for non-labelling control, 25 µL of activity buffer was mixed with 25 µL of protein.

Semi-preparative / preparative RP-HPLC

Preparative or semi-preparative HPLC was performed on an Agilent 1260 Infinity II LC System equipped with columns as followed: preparative column – Reprospher 100 C18 columns (10 µm: 50 x 30 mm at 20 mL/min flow rate; semi-preparative column – 5 µm: 250 x 10 mm at 4 mL/min flow rate. Eluents A (0.1% TFA in H2O) and B (0.1% TFA in MeCN) were applied as a linear gradient. Peak detection was performed at maximal absorbance wavelength (at 220, 254, 500, 550, 615 or 750 nm).

Cell culture

COS-7 and HEK293T cells were obtained from the German Collection of Microorganisms and Cell Cultures (DSMZ) and cultured in Dulbecco's Modified Eagle's Medium (DMEM) supplemented with 10% FCS, 2 mM L-glutamine and penicillin (100 units/mL) / streptomycin (100 µg/mL) in a humidified atmosphere at 37 °C and 5% CO2 (v/v). COS-7 and HEK293T cells were maintained in T175 flasks and split 1:5 or 1:10, depending on given confluence.

Dimerization studies for Western blot analysis

For dimerization cell studies, 150.000 HEK cells/ well were seeded in 1 mL DMEM full medium in a 12 well plate. On the following day, cells were transfected with desired constructs (500 ng DNA per well) using Lipofectamine2000 or PEI according to the manufacturer's instructions. 24 hours post transfection cells were washed once with PBS and starved for 3-4 hours in 0.5 mL starvation medium. Following starvation, the medium was aspirated, and cells were stimulated if not stated otherwise with 0.3 nM BMP9, 0.2 nM TGFβ1, 3.7 nM BDNF respectively or 0.5/1 µM of respective BG-PEG_2-HTL, BG-PEG_6-HTL, BG-PEG_{12}-HTL, MT36, MT37 dimerizer in 200 µL starvation medium per well for 30 minutes at 37°C. For pre-dimerization experiments, cells were treated with respective dimerizer molecules for 15 minutes at 4°C before addition of respective ligands in a total volume of 200 µL starvation medium per well for 30 minutes at 37°C. For Halo-tag blocking experiments, cells were treated with HaloTag® Alexa Fluor® 660 Ligand (Promega) before stimulation with the dimerizer molecules. Unstimulated control samples were incubated under equal conditions in 200 µL starvation medium. After stimulation cells were washed once with PBS and lysed with 150 µL Laemmli buffer per well. Cell lysate was stored at -20°C until further usage.

SDS-PAGE & Western-blotting

For sodium dodecyl sulfate polyacrylamide gel-electrophoresis (SDS-PAGE), respectively treated cells were lysed in 150 µL Laemmli buffer and frozen at -20 °C. To ensure a homogeneous loading, cell lysate was pulled through a 1 ml syringe and boiled for 10 min at 95°C before loading onto 10% polyacrylamide gels. After performed gel-electrophoresis, proteins were transferred onto Methanol-activated PVDF membranes by Western-blotting. Next, membranes were blocked for 1 hour in a solution containing 0.1% TBS-T and 3% w/v bovine serum albumin (BSA), then washed three times in 0.1% TBS-T and incubated with indicated primary antibodies overnight at 4°C. Primary antibodies: anti-GAPDH (Cell Signaling; #2118; monoclonal rabbit antibody), anti-Halo (ProMega; #G9211; monoclonal mouse antibody) anti-SNAP (NEB; P9310S; polyclonal rabbit antibody); P-p44/42 MAPK (CellSig; #4370; monoclonal rabbit antibody); p44/42 MAPK (CellSig; #9102; polyclonal rabbit antibody); pSMAD1/5/8 (CellSig; #41D10; monoclonal rabbit antibody); pSMAD2/3 (CellSig; #3108S; monoclonal rabbit antibody); total SMAD2/3 (CellSig; #8685; monoclonal rabbit antibody); total

SMAD1 (CellSig; #D59D7; monoclonal rabbit antibody) were used at a 1:1000 dilution in 3% w/v BSA/ TBS-T solution. For HRP-based detection, membranes were incubated with secondary goat-α-mouse or goat-α-rabbit IgG HRP conjugates (± 0.8 mg/ml, Dianova; #111-035-144, #115-035-068) for 1 hour at a dilution of 1:10000. Chemiluminescent reactions were processed using WesternBright Quantum HRP substrate (advansta) and documented on a FUSION FX7 digital imaging system.

Emission- and FRET-profiles of fluorescent dimerizer on purified proteins

100 µL of 400 nM Crosslinker/Dye + 100 µL of 2 µM Snap/Halo/Snap-Halo Protein diluted in activity buffer were transferred into Greiner black flat bottom 96 well plates and emission profiles were recorded on a TECAN INFINITE M PLEX plate reader. To obtain 'degree of dimerization' 100 µL of 500 nM SNAP and 100 nM MT36 + 0, 25, 50, 75 and 100 nM HTP was used. Raw emission intensity values were measured triplicates. Data normalization, integration and plotting was performed in GraphPad Prism 9.3. JF_{549}: λ_{Ex} = 530±10 nm; λ_{Em} = 550–800±20 nm; 25 flashes; 20 µs integration time; JF_{646}: λ_{Ex} = 630±10 nm; λ_{Em} = –800±20 nm; 25 flashes; 20 µs integration time or λ_{Ex} = 500±10 nm; λ_{Em} = 540–800±20 nm; 5 flashes; 20 µs integration time.

Emission- and FRET-profiles of fluorescent dimerizer on cells

For receptor proximity cell studies, 50.000 HEK cells/ well were seeded in 100 µL DMEM full medium in a black Nunc™ MicroWell™ 96-Well, Nunclon Delta-Treated, Flat-Bottom Microplate. On the following day, each well was transfected with a Halo and SNAP receptor pair (200 ng DNA per well) using Lipofectamine2000 according to the manufacturer's instructions. For one receptor pair condition 12 wells are needed in total, from which four wells are for the measurements of the Halo tagged receptor, four wells are for the measurements of the SNAP tagged receptor and four wells are to measure the receptor proximity. 24 h after transfection, medium was then aspirated following incubation with either HaloTag® Alexa Fluor® 488 Ligand (Promega) or SNAP-Surface® Alexa Fluor® 488 (NEB) (2 µM, in a total volume of 50 µL) at 4 °C for 10 minutes in the respective wells to block either the Halo or SNAP tagged receptor of each pair, while wells for measuring induced proximity were incubated with PBS. Finally, the block solution and PBS were removed and the MT36 (0.5 µM, in a total volume of 50 µL) was added for 15 minutes at 4 °C. After incubation cells were washed once with ice cold PBS and kept at 4°C till immediate fluorescent measurement with a TECAN Spark plate reader. Raw emission intensity values were measured as quadruplicates. Data normalization, integration and plotting was performed in GraphPad Prism 9.3. MT36: λ_{Ex} = 500±50 nm; λ_{Em} = 550–840±20 nm; step size 2; bandwidth 10 nm.

Confocal and lifetime microscopy

Confocal data of fixed COS-7 cells were acquired with the Expert Line STED Microscope from Abberior. 200.000 COS-7 cells / well were seeded in 1 mL DMEM full medium either on glass cover slips or directly in a 12 well plate. On the following day, cells were transfected with desired constructs (500 ng DNA per well) using Lipofectamine2000 according to the manufacturer's instructions. For experiments including IF stainings, cells were trypsinized 6 hours after transfection and approximately 50.000 cells were reseeded in Ibidi glass bottom 6 channel µ-slides. 24 hours post transfection cells were washed once with PBS and incubated with MT36/MT37 (0.5 µM) for 15 minutes on ice. Subsequently, the staining solution was aspirated, and cells were washed once with ice-cold PBS before fixation with 4 % PFA for 10 minutes at room temperature. To remove the PFA, cells were washed twice with PBS followed by quenching with 50 mM NH4Cl in PBS for 5 minutes at room temperature. Cells were then permeabilized with a 0.5% Triton X-100/PBS solution for 10 minutes and washed twice with PBS. For IF stainings, this was followed by incubating the cells with a 3% BSA blocking solution for 1 hour at room temperature and final addition of the primary antibody (Cell Signaling; Phospho-p44/42 MAPK (Erk1/2) (Thr202/Tyr204) antibody #9101, 1:250) at 4 °C overnight. The next day, cells were washed 3 times using PBS and incubated with the secondary antibody (Invitrogen; Alexa Fluor 488 goat anti-rabbit antibody #A11034; 1:400) for 1 hour at room temperature. To stain the nucleus, the cells were incubated with a DAPI-solution (1:1000 in PBS) for 15 minutes, then washed three times and finally mounted with Fluoromount G or liquid mounting medium (Ibidi). Finally, confocal images of COS7 cells expressing Halo and SNAP-constructs stained with DAPI, MT36/37 or JF_{549}-BG and JF_{646}-HTL with or without fluorescent antibody (Alexa Fluor 488), were acquired on a commercial expert line Abberior STED microscope, using the Imspector software from Abberior Instruments (Version 16.3) in line-scanning confocal mode. The microscope was equipped with 405 nm, 485 nm, 561 nm, 640 nm excitation lasers. The detection windows were set to 415 – 580 nm, 500 – 540 nm, 570 – 630 nm, 650 – 750 nm. For assessing the FRET channel, 561 nm excitation laser was used, and the emission detection window was set to 700 – 750 nm. The laser power was kept for DAPI at 2% and for all other channels at 10%. Each channel was acquired for cells expressing either Halo or SNAP-tagged receptors individually or together. Consecutive image analysis was performed with Fiji imageJ, where each cell was treated as an individual region of interest (ROI). The selection of the ROI (cell) was facilitated by using the wand tool on either red or infrared channel with the highest fluorescence intensity and setting the corresponding threshold to solely select the respective cell without background. Respective ROI selection was added to the ROI manager and then used on the different channels of the same cell to assess the raw integrated density (RawIntDen) and cell area. The RawIntDen describes the sum of all pixel intensity values within a selected region of Interest. Normalization of the

RawIntDen to the cell area allows then for comparison between different cells/conditions. The resulting normalized mean fluorescence intensity per cell of the FRET channel was then used to display the distribution of single cell FRET emission values of the different bound states (Halo, SNAP or double bound) of MT36 and JF549/JF646 across the whole data set (10 cells per condition, with n=3 independent experiments – 30 cells in total per condition). Further, data was averaged for 10 cells per independent experiment and displayed as mean FRET intensities of each n. The fold induction (F.I.) was calculated for FRET & pERK1/2 by dividing the respective mean SNAP-only or SNAP/Halo condition intensity value by the mean Halo-only receptor value of each n. Finally, a line scan for the representative FRET images of TrkB and mGluR2 conditions was extracted.

For fluorescence lifetime imaging, drops of 20 µl of 0.1 µM SNAP:JF_{549} or 1 µM SNAP:MT36:Halo in PBS where spotted in u-Slide 8 Well Glass Bottom (Ibidi #80877). Fluorescence lifetime was measured on a Leica SP8 TCS STED FALCON (Leica Microsystems) equipped with a pulsed white-light excitation laser (80 MHz repetition rate, NKT Photonics), a 100× objective (HC PL APO CS2 100×/1.40 NA oil), a temperature controlled chamber at room temperature, operated by LAS X, excited at 561 nm and the lifetime of the collected em from λ = 576-620 nm in triplicates with multiple ROIs. A Hybrid detector produces FLIM images of 512 × 512 pxl with 113 nm per pxl after 10 frame repetitions. Solution of 0.1 µM Fluorescein was used as a reference, excited at 488 nm and the lifetime of the collected em from λ = 503-540 nm was 3.8 ns. Fluorescence lifetime decay curves from selected regions were fitted with one exponential function and the lifetime is reported for each region. The average FRET efficiency was calculated according to Supplementary Figure 6, and yielded 23%. While this is lower than an expected E_{FRET} = >99% according to the Förster equation for distance <3 nm, this may have multiple reasons:

1. The orientation factor κ^2 is unknown, which is affecting E_{FRET}
2. To obtain reasonable fluorescent signal, the concentration of SNAP:MT36:Halo was 10-fold higher than SNAP:JF_{549}, as well as laser intensity was also 10-fold higher. This speaks highly of a way dimmer signal, caused by a more efficient energy transfer.
3. The fluorogenicity of JF_{646} is unknown, and since this is dependent on the microenvironment, it will affect E_{FRET} if in the closed state.
4. The decay curves were fitted mono-exponentially with the inbuilt Leica software. When fitting with a bi-exponential, one decay rate was 0.2 ns. However, for better comparison, we stuck to solely mono-exponential fitting.

Statistical analysis and graphical design

All statistical tests were performed using GraphPad Prism version 9.3 software and are listed in the figure legends. Normal distribution of data sets was tested with one-way ANOVA and Tukey’s multiple comparisons test or two-way ANOVA and Šídák's multiple comparisons test. For all experiments statistical significance was assigned, with an alpha-level of $p < 0.05$. Figures were assembled using Biorender and Adobe® Photoshop (Adobe Systems, San José, USA).
